# Effects of Changes in Food Supply at the Time of Sex Differentiation on the Gonadal Transcriptome of Juvenile Fish. Implications for Natural and Farmed Populations

**DOI:** 10.1371/journal.pone.0111304

**Published:** 2014-10-23

**Authors:** Noelia Díaz, Laia Ribas, Francesc Piferrer

**Affiliations:** Institut de Ciències del Mar, Consejo Superior de Investigaciones Científicas (CSIC), Barcelona, Spain; University of Hyderabad, India

## Abstract

**Background:**

Food supply is a major factor influencing growth rates in animals. This has important implications for both natural and farmed fish populations, since food restriction may difficult reproduction. However, a study on the effects of food supply on the development of juvenile gonads has never been transcriptionally described in fish.

**Methods and Findings:**

This study investigated the consequences of growth on gonadal transcriptome of European sea bass in: 1) 4-month-old sexually undifferentiated fish, comparing the gonads of fish with the highest vs. the lowest growth, to explore a possible link between transcriptome and future sex, and 2) testis from 11-month-old juveniles where growth had been manipulated through changes in food supply. The four groups used were: i) sustained fast growth, ii) sustained slow growth, iii) accelerated growth, iv) decelerated growth. The transcriptome of undifferentiated gonads was not drastically affected by initial natural differences in growth. Further, changes in the expression of genes associated with protein turnover were seen, favoring catabolism in slow-growing fish and anabolism in fast-growing fish. Moreover, while fast-growing fish took energy from glucose, as deduced from the pathways affected and the analysis of protein-protein interactions examined, in slow-growing fish lipid metabolism and gluconeogenesis was favored. Interestingly, the highest transcriptomic differences were found when forcing initially fast-growing fish to decelerate their growth, while accelerating growth of initially slow-growing fish resulted in full transcriptomic convergence with sustained fast-growing fish.

**Conclusions:**

Food availability during sex differentiation shapes the juvenile testis transcriptome, as evidenced by adaptations to different energy balances. Remarkably, this occurs in absence of major histological changes in the testis. Thus, fish are able to recover transcriptionally their testes if they are provided with enough food supply during sex differentiation; however, an initial fast growth does not represent any advantage in terms of transcriptional fitness if later food becomes scarce.

## Introduction

Food availability and energetic demands fluctuate in most habitats. Animals are capable of sensing their inner energy levels and the external energy availability and thus act accordingly by long-term investments in processes like growth, immune functions or reproduction when food availability is not a problem, or by ensuring survival when food is scarce [1]. In fish, there is a tight relationship between food availability and reproduction [1], [2] since it can alter the timing and duration of spawning, fecundity and egg size [3], [4], or the timing of the reproductive cycles [5]. Favorable feeding conditions produce early maturation of individuals [6] while a decrease in food availability causes a decrease in energy transfer to the gonads [7], but this relationship may present important differences between species since fish constitute a vast phylogenetic group with different behaviors and reproduction types [8].

The European sea bass (*Dicentrarchus labrax*) is a gonochoristic species with a polygenic sex determining system [9] presenting a long sexually undifferentiated process with sexual dimorphism at the time of sex differentiation onset (∼150 days post hatch, dph, for females and ∼180 dph for males) [10]–[13]. However, this dimorphism is more related to the attained length than to age [14]. The relationship between growth and sex differentiation has been previously studied in sea bass [9], [12]–[19]. There is a relationship between body weight and sex since not only sea bass females are larger than males, but also both males and females in batches with higher percent females were bigger than males and females of batches with a lower female percent [9]. Further, early size-gradings of the population (at 66 and 123–143 dph, [13]; at 70 dph, [15]; or at 82 dph, [17]) selecting for the largest fish resulted in ∼90% of females, but the opposite, i.e., selecting for the smallest fish, produced only ∼65% males at one year of age, meaning that while the largest fish are essentially all females, among the smallest fish there are both males and females [13].

Recently, two experiments on growth rate alteration by manipulating food supply during the sex differentiation period were conducted with the European sea bass in our laboratory [19]. The first experiment showed that transiently but severely reducing food supply starting towards the end, middle or even at the beginning of the sex differentiation period – and thus negatively affecting growth – did not affect resulting sex ratios, indicating that gender was already fixed before the above mentioned period started [19]. The second experiment involved four groups of fish, which, through controlling food supply, were made to experience different growth rates during the sex differentiation period. Two groups, one fast-growing and the other slow-growing, originated from the fast-growing fish at 127 dph. The other two groups, also one fast-growing and the other slow-growing, originated from the slow-growing fish at 127 dph. In this case, there were differences in the final sex ratio of the population as fast-growing-derived groups presented more number of females (∼40%) than the slow-growing-derived groups (∼10%). Thus, the differences in the final sex ratio were not related to the growth rate during the sex differentiation but to whether fish derived from the fast- or slow-growing fish at 127 dph. These results confirmed the results of the first experiment and indicated that before the first signs of sex differentiation appear the relationship between growth and sex is already established, confirming that in the European sea bass larger sizes are associated with female development [13], [19].

Partition of consumed energy into growth, energy storage and gonads according to temporal food availability, metabolic demands and reproductive needs have been studied since a long time ago [20]. Recently, with the advent of new technologies, the underlying mechanisms including associated changes in global gene expression can be investigated. However, transcriptomic analyses in fish have traditionally addressed nutrition and reproduction topics separately. Hence, while growth studies have put efforts towards the effects of diet substitutions [21]–[24], stocking density and food ration [25]–[27] and comparing domesticated vs. transgenic fish [28], on the other hand, reproduction-related transcriptome analyses have focused on describing changes associated with gonad maturation and differences between sexes [29]–[32], environmental effects [33] or hormonal treatment effects [34], [35].

However, a study directly analyzing the effects of food supply on reproduction and, particularly, on the development of juvenile gonads has never been described in fish. In mitten crab (*Eriocheir sinensis*) during early development, when crabs store significant amounts of energy in the hepatopancreas, Jiang and collaborators [36] found four genes in the hepatopancreas and 13 genes in testis related to nutritional control, and three genes in the hepatopancreas and eight in the testes related to regulation of reproduction. Among the former, arginine kinase, zinc-finger proteins or leptin were upregulated in the hepatopancreas transcriptome as a sign of energy storage for further energy-demand of the reproductive processes. Genes involved in the regulation of reproduction, such as cyclins, kinetochore spindle formation or the heat shock protein 70, were upregulated in testis and promoted an increase in cell division during spermatogenesis. In rats, dietary energy intake changes (restrictions and excesses) but also food availability had profound effects in gonads from both sexes at different levels (biochemical, endocrine, behavioral and genetic) [37]. Moreover, a transcriptomic analysis of the gonads of these rats facing diet restriction or excess showed how females were more affected by ration changes than males. Males were also better adapted to an intermittent fasting by increasing the probability of an eventual fertilization, while females were able to sense the food restriction and behaved as sub-fertile females [38].

The present study is based on samples collected in experiment 2 of Díaz et al. [19] described above and had two objectives: 1) to analyze the transcriptional differences in sexually differentiating European sea bass gonads from the naturally fastest growing vs. the slowest growing fish at 127 dph, i.e., before the first histological signs of sex differentiation at 150 dph [13], but after the first signs of molecular sex differentiation at 120 dph [39], to explore a possible link of transcriptomic signatures with future sex; and 2) the consequences of food availability between 133–337 dph (juvenile growth) on the subsequent testes transcriptome by analyzing the effects of growth acceleration and deceleration.

## Materials and Methods

### Animals and rearing conditions

As stated above, the fish that were transcriptomically analyzed in this study are the same fish described in Experiment 2 of Díaz et al. [19]. Briefly, European sea bass eggs obtained from a commercial hatchery were collected at one day post fertilization (dpf) on March 2009, transported to our experimental aquarium facilities and hatched following established procedures for this species [40] with minor changes, as previously described [18], [19]. Fish were reared under natural conditions of photoperiod, pH (∼7.9), salinity (∼37.8 ppt), oxygen saturation (85–100%) and with a water renewal rate of 30% vol·h^−1^. In order to avoid temperature influences on the sex ratio, the thermal regime used and previously described [19] included egg spawning at 13–14°C and larval rearing at 16±1°C until 60 dpf. Then, temperature was increased to 21°C at a rate of 0.5–1°C·day^−1^ and maintained until the first fall, when it was let to follow the natural temperature. The rearing density was kept low to avoid any possible distorting effect on sex ratios (details in [19]). Fish were manually fed three times a day with artemia AF, then artemia EG enriched with amino acid (INVE Aquaculture, Belgium) and dry food (ProAqua, S.A., Spain) of the appropriate pellet size as fish grew. Unless otherwise stated, juveniles and adults were fed *ad libitum* two times a day.

Fish were treated in agreement with the European Convention for the Protection of Animals used for Experimental and Scientific Purposes (ETS Nu 123, 01/01/91). Our facilities are approved for animal experimentation by the Ministry of Agriculture and Fisheries (certificate number 08039-46-A) in accordance of Spanish law (Real Decreto 223 of March 1988) and the experimental protocol was approved by the Spanish National Research Council (CSIC) Ethics Committee within project AGL2010-15939. Animals were sacrificed by an overdose of 2-phenoxyethanol (2PE) followed by severing of the spinal cord.

### Experimental design

**Figure 1:**
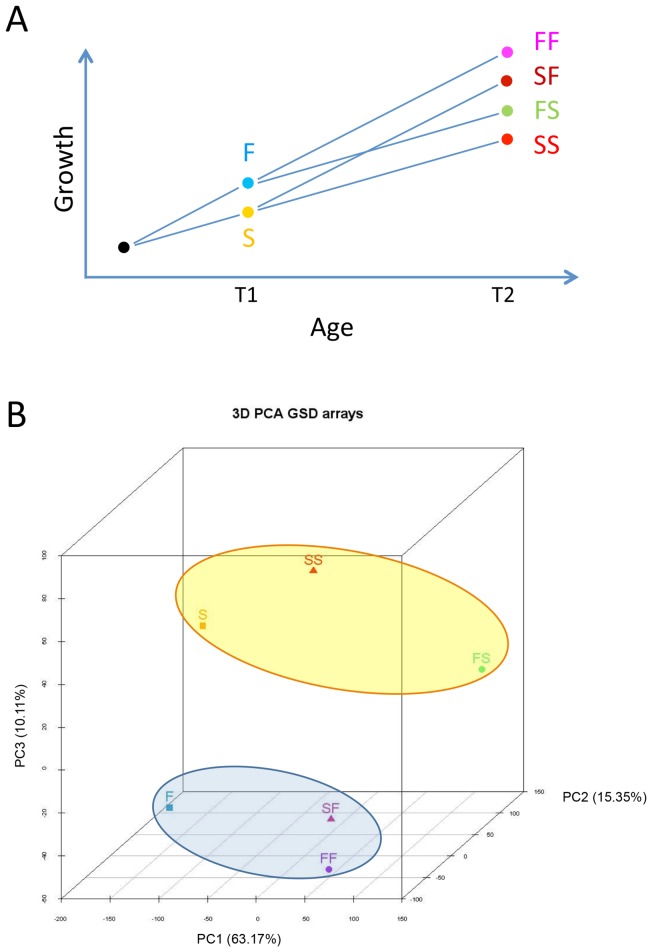
Experimental design and overall transcriptomic results. A) Experimental design involving European sea bass subjected to food restriction at different times during the sex differentiation period. B) Principal component analysis representation of transcriptomic results of the six groups. Time 1 fast- (F) and slow- (S) growing groups, and time 2 F- (FF and FS) and S- (SF and SS) derived groups.

Details of the experimental design can also be found in Díaz et al. [19]. Briefly, fish were individually size-graded at 127 dph, i.e., at ∼4 cm standard length (SL), before the histological process of sex differentiation started (∼8 cm SL), and separated into two groups according to the SL they had attained and comprising the two extremes of the normal curve distribution: a fast growth group (group F), with a mean size of 5.0 cm SL (range 4.2–6.4 cm), and a slow growth group (group S), with a mean size of 3.5 cm SL (range 2.6–3.7 cm). After checking that fish of each group was of the desired size, then at 133 dph (time 1, T1, i.e., when fish were 4 months old) each group was further subdivided into two tanks (n = 79 fish per tank). On one hand, the F group was subdivided into two groups with initial similar mean SL and BW: the fast-fast group (FF), in which growth rates from that moment onwards were as before, and the fast-slow group (FS), in which the growth rate was reduced to match what had been the growth rate of group S until then. On the other hand, the S group was also subdivided into two groups with initial similar mean SL and BW: the slow-fast group (SF), in which the growth rate was increased to match what had been the growth rate of group F until then, and the slow-slow group (SS), in which the growth rates from that moment onwards were as before (see Fig. 1A for a diagram of the experimental design). Food supply changed as follows: prior to T1, all fish were fed *ad libitum* three times a day. Then, groups FF and SF (accelerated growth) were fed *ad libitum* four times a day, with an amount of food per day equivalent of 3–6% of their mean BW. On the other hand, groups FS and SS (decelerated growth) were feed only two times a day with an amount of food per day equivalent of 1.5–3% of their mean BW. The growth rate of all groups was carefully monitored by periodic samplings and the amount of food adjusted if necessary. Animals were sacrificed when they were 337 dph juveniles (T2, i.e., when fish were 11 months old) (range of fish per tank at that moment: 52–70). There was no mortality associated to treatments.

### Samplings

Details on the follow-up of growth, including sexual growth dimorphism, gonadosomatic, hepatosomatic and carcass indices, as well as sex ratio and the degree of gonadal development of these fish have been previously described [19]. Fish used for objective 1 were sexually undifferentiated. For objective 2, when possible, sex was visually determined and confirmed histologically if necessary [19]. Only males were considered for objective 2 since the goal was to study the relationship between growth during sex differentiation on the subsequent juvenile testis transcriptome. Histological results indicated that testis contained no spermatozoa. We did not do a similar study on females because some of the resulting populations were highly male-biased (∼90% males) and thus we did not have enough individual females in all groups. The number of fish used for each group and the biometry is shown in Table S1. Here, we focus only on the RNA extraction for transcriptomic analysis of gene expression and for microarray validation by qRT-PCR.

### RNA extraction and cDNA synthesis

Total RNA was extracted from sexually undifferentiated gonads (mean SL ∼4 cm) at 133 dph (T1) and sexually differentiated juvenile testis at 337 dph (T2).

A classical chloroform-isopropanol-ethanol RNA extraction protocol after a Trizol (Live Technologies, Scotland, UK) homogenization was used. RNA quality and concentration were measured by a ND-1000 spectrophotometer (NanoDrop Technologies) and checked on a 1% agarose/formaldehyde gel. RNA integrity was measured by a Bioanalyzer 2100 (RNA 6000 Nano LabChip kit Agilent, Spain). Samples with a 100–200 ng/µl RNA concentration and RIN>7 were used for microarray hybridizations.

In parallel, 200 ng of total RNA were treated by *E. coli* DNAse H and retrotranscribed (100 ng) to cDNA using SuperScript III RNase Transcriptase (Invitrogen, Spain) and Random hexamer (Invitrogen, Spain) following manufacturer's instructions.

### Microarray. Experimental design

Hybridizations were performed at the Universitat Autònoma de Barcelona (UAB, Spain). The experiment included, on one hand, the comparison of 15 undifferentiated gonads from two groups (seven individual gonads from the F group against eight individual gonads from the S group) at 133 dph (T1), to explore transcriptomic differences between two groups with different growth rates since they were selected from the opposite extremes of the normal distribution curve. On the other hand, individual testes from each one of the four groups (groups FF, SF, FS and SS) differentially fed and sampled at 337 dph (T2), were analyzed to investigate the growth acceleration or growth deceleration effects on their transcriptome. Thus, 35 microarrays (one per fish) were used to analyze the gonadal transcriptome of the six groups considered in this study. To avoid batch effects samples were evenly distributed among the slides.

### Microarray. RNA sample preparation and array hybridization

RNA labelling, hybridizations, and scanning were performed according to the manufacturer's instructions. Briefly, total RNA (100 ng) was amplified and Cy3-labeled with Agilent's One-Color Microarray-Based Gene Expression Analysis (Low Input Quick Amp Labelling kit) along with Agilent's One-Color RNA SpikeIn Kit. Then cRNA was purified with RNeasy mini spin columns (Qiagen), quantified with the Nanodrop ND–1000 and verified using the Bioanalyzer 2100. Each sample (1.65 µg) was hybridized to the *Dicentrarchus labrax* array (Agilent ID 023790) at 65°C for 17 h using Agilent's GE Hybridization Kit. Washes were conducted as recommended by the manufacturer using Agilent's Gene Expression Wash Pack with stabilization and drying solution. Arrays were scanned with Agilent Technologies Scanner, model G2505B. Spot intensities and other quality control features were extracted with Agilent's Feature Extraction software version 10.4.0.0. The experiment has been submitted to Gene Expression Omnibus (GEO)-NCBI database (GSE54362) and the platform that validates the microarray has the accession number (GPL13443).

### Quantitative real time PCR (qRT-PCR)

Microarray validations were carried out by qRT-PCR analysis. Two genes from each one of the six possible microarray comparisons (see Table S2) were used for qRT-PCR validation, including one up- and one downregulated gene. Primers were designed using Primer 3 Plus (http://www.bioinformatics.nl/cgi-bin/primer3plus/primer3plus.cgi) against the annotated gene sequences directly from the European sea bass genome (Tine et al., 2014, unpublished), always trying to design the primers between exons to avoid DNA contamination (Table S2). Primer amplification efficiencies were tested by linear regression analysis from a cDNA dilution series and by running a melting curve (95°C for 15 s, 60°C for 15 s and 95°C for 15 s). Efficiency (*E* = 10^(−1/slope)^, with values between 1.80 and 2.20), standard curves ranging from –2.9 to –3.9 and linear correlations (*R^2^*) higher than 0.92 were recorded (Table S2). cDNA was diluted 1∶10 for all the target genes and 1∶500 for the endogenous control (the housekeeping gene *r18S*).

qRT-PCR was analyzed by an ABI 7900HT (Applied Biosystems) under standard cycling conditions. Briefly, an initial UDG decontamination cycle (50°C for 2 min), an activation step (95°C for 10 min) was followed by 40 cycles of denaturation (95°C for 15 s) and one annealing/extension step (60°C for 1 min). A final dissociation step was also added (95°C for 15 s and 60°C for another 15 s). Each sample was run in triplicate in 384-well plates in a final 10 µl volume (2 µl of 5x PyroTaq EvaGreen qPCR Mix Plus (ROX) from Cultek Molecular Bioline, 4 µl distilled water, 2 µl primer mix at a 10 µM concentration and 2 µl of cDNA). Negative controls were run per duplicate and *r18S* was used to calculate intra- and inter-assay variations. SDS 2.3 software (Applied Biosystems) was used to collect raw data and RQ Manager 1.2 (Applied Biosystems) was used to calculate gene expression. qRT-PCR data was analyzed by adjusting for *E* and normalizing to the *r18S* reference gene [41].

### Statistical analysis of data. Microarray raw data normalization

Feature Extraction output data was corrected for background using normexp method [42] and was quantile normalized [43]. Reliable probes showed raw foreground intensity at least two times higher than the respective background intensity and were not saturated nor flagged by the Feature Extraction software. Our sea bass custom-made microarray presents most of the probes (64.7%) in duplicate but also with more than three identical probes for some genes. Median intensities per gene were calculated and a probe was considered reliable when at least half of its replicates were reliable as defined above. An empirical Bayes approach on linear models (Limma) [44] was used to perform a differential expression analysis. A False Discovery Rate (FDR) method was used to correct for multiple testing. Differentially expressed (DE) genes were filtered by fixing an absolute fold change (FC) of 1.5 and an adjusted *P*-value <0.01. MA data analyses were performed with the Bioconductor project (http://www.bioconductor.org/) in the R statistical environment (http://cran.rproject.org/) [45].

### Statistical analysis of data. qRT-PCR statistics

Quantitative qRT-PCR statistical analysis was performed using 2DCt from the processed data [41]. 2DCt results were then checked for normality, homoscedasticity of variance and a one-way ANOVA test was used to assess differences between treatments using SPSS v.19 software.

### Gene annotation and enrichment analysis

Gene data (names, abbreviations, synonyms and functions) were determined using Genecards (http://www.genecards.org/) and Uniprot (http://www.uniprot.org/). The web based tool AMIGO (http://amigo.geneontology.org/cgi-bin/amigo/go.cgi) [46] was used to look for the sequences of the DE genes found at the MA. After obtaining these sequences, Blast2GO software (http://www.blast2go.com) [47] was used to enrich GO term annotation and to analyze the subsequent altered KEGG pathways (http://www.genome.jp/kegg/), which were also further explored by DAVID (http://david.abcc.ncifcrf.gov/; [48], [49]). Completing the analysis, Blast2GO with Fisher's Exact Test with Multiple Testing Correction of the False Discovery Rate [50] was used to analyze our DE genes using the custom-made microarray as background.

Protein names from the DE genes were then uploaded to the web-based tool STRING v9.1 (http://string-db.org/) [51] to analyze physical and functional protein interactions. Furthermore, an FDR test was applied to determine if the protein list was enriched (higher values meaning higher significances). A Mean Linkage Clustering (MLC or UPGMA), a simple agglomerative hierarchical clustering included in STRING was performed to group proteins. This method clusters proteins based on pairwise similarities in relevant descriptor variables.

## Results

### Overall assessment of transcriptomic results

Visualization of the spatial distribution of the microarray data of the six studied groups along the three major axis of the PCA is shown in Figure 1B. Component 1 contributed to 63.17% of the variation while the first three components together explained 88.63% of the variation. Two clusters could be observed, one containing group F and the F-derived groups with an accelerated growth (groups FF and SF), and the other formed by group S and the S-derived groups with growth deceleration (groups SS and FS).

**Figure 2:**
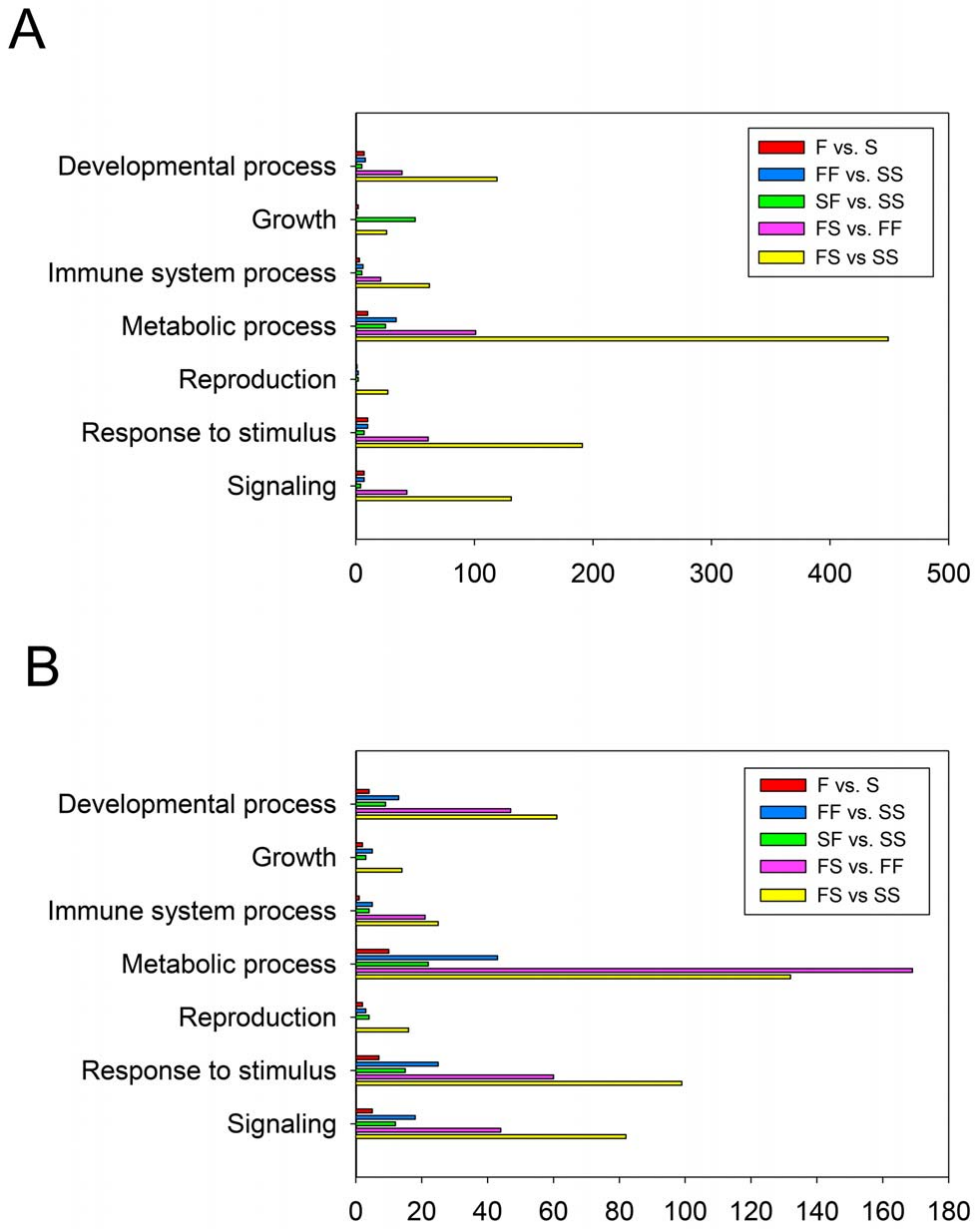
The seven selected GO subcategories for the Biological process (BP) category. A) Upregulated GO-terms and B) downregulated GO terms in the different studied comparisons.

The number of DE genes found in the only possible comparison at T1 as well as in the six possible comparisons between the four groups at T2 is shown in Table 1. The comparison with larger number of genes was FS vs. SS, while the FF vs. SF comparison gave no DE genes. From each one of the comparisons with DE genes, the most upregulated and the most downregulated genes (a total of twelve) were selected for a qRT-PCR validation (see details and quality control data of the designed primers in Table S2). All genes tested showed the same fold change tendency, thus validating the microarray results (Table 2). Among the tested genes four of them (*cct6a*, *rps15*, *fabp3* and *rpl9*) showed statistical differences (*P*<0.05) when analyzed by qRT-PCR. In the comparisons containing DE genes, analysis of the associated GO terms related to biological processes (BP), molecular function (MF) and cell component (CC) provided further information on the molecular signatures of each treatment (Table S3). Seven selected BP subcategories based on prior knowledge that they take place in the gonads are shown in Figure 2. Metabolic process, response to stimulus and signaling were, in that order, the most represented subcategories. Regarding the MF and CC subcategories, no clear differences were seen among the different comparisons. The most represented MF subcategories among the comparisons were binding and catalytic activities.

**Table 1:** Differentially expressed genes in the different comparisons.

Group comparisons	Total # of genes	# Upregulated genes			# Downregulated genes		
		Total	Real	NA	Total	Real	NA
F vs. S	76	41	20	11	35	20	6
FF vs. SS	155	71	43	9	114	70	30
FS vs. FF	1092	662	316	47	431	153	111
SF vs. SS	94	42	26	1	53	37	9
SF vs. FS	938	507	184	162	604	303	40
FF vs. SF	0	0	0	0	0	0	0
FS vs. SS	2014	1452	717	108	562	261	202

NA, non annotated genes.

**Table 2:** Microarray validation by qRT-PCR.

Comparison	Gene	Microarray FC	Microarray adj. *P*-value	qRT-PCR FC	qRT-PCR SEM	qRT-PCR Student *t*-test
F vs. S	*flna*	2.85	0.004	1.801	0.509	0.332
	*tspan1*	−5.90	0.007	−2.47	0.185	0.356
FF vs. SS	*cct6a*	2.33	0.001	2.98	0.551	0.009
	*rps15*	−13.28	0.000	−1.46	0.407	0.001
FS vs. FF	*ggps1*	11.58	0.006	22.63	16.673	0.284
	*fabp3*	−15.34	0.007	−7.51	0.125	0.001
SF vs. SS	*rpl9*	2.61	0.001	18.79	7.023	0.047
	*pcca*	−14.03	0.000	−42.13	0.009	0.970
SF vs. FS	*lpl*	13.93	0.006	2.23	0.440	0.364
	*tspan13*	−10.27	0.006	−240.52	0.002	0.204
FS vs. SS	*ca1*	36.70	0.004	2.49	0.591	0.631
	*agpat5*	−13.38	0.000	−2.38	0.292	0.849

### Transcriptome of sexually undifferentiated gonads of initial fast-growing vs. initial slow-growing fish (group F vs. group S comparison)

**Figure 3:**
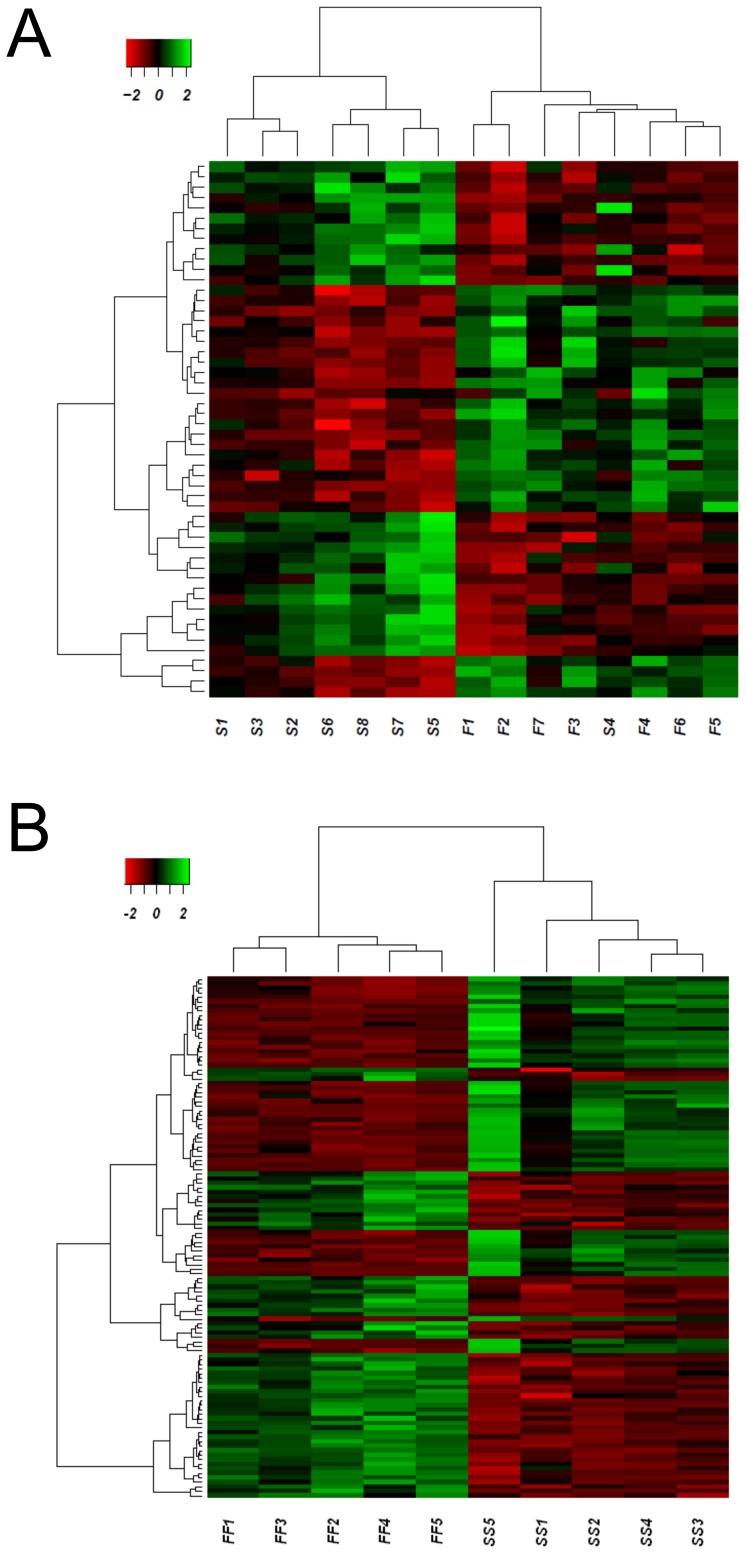
Individual heatmap representation of the transcriptome analysis. A) undifferentiated gonads of F versus S growing fish. B) differentiated testis of sustained fast (FF) or slow (SS) growing fish. Only DE genes are represented in the figure. High to low expression is shown by a degradation color from green to red, respectively. The scale bar shows Z-score values for the heatmap.

All fish from the F group clustered together and all but one fish from the S group did the same as shown in the heatmap (Figure 3A). Of the total 40 DE genes, among the 20 upregulated there were genes related to transcription, immune response or cytoskeleton structure, whereas among the 20 downregulated ones there were genes mainly related to mitochondrial functions (Table S4).

Further analyzing the BP subcategories for the up- and downregulated GO terms (Figure 2A and 2B, respectively) showed how the number of GO terms for all the subcategories was always low when compared with T2 group comparisons. Tyrosine-protein kinase gene, a gene involved in male gonad development, was upregulated in the F group. In general, genes related to the response to stimulus and metabolic processes were downregulated. This applied also to genes related to growth such as growth hormone (*gh*) and adrenomedullin, which is related to male gonad development and response to stimulus.

DAVID analysis of DE genes showed two gene clusters within the upregulated genes related to cytoskeleton organization and lumen (enrichment scores 2.6 and 1.14, respectively), and five clusters within the downregulated genes mainly related to mitochondrion, binding, membrane structure and ion binding (enrichment scores 3.36, 1.56, 1.47, 0.31 and 0.22, respectively). KEGG pathway analysis of DE genes showed nine affected pathways: three were upregulated and included T-cell receptor signaling and linoleic acid metabolism, and six were downregulated and mainly related to the metabolism of xenobiotics and amino acid degradation (Table S5).

Only seven interactions were found among the proteins coded by these DE genes; however, they were enriched in interactions (*P*<0.001). Proteins from the upregulated DE genes were required for the 60S ribosomal subunit biogenesis and mRNA stability and included proteins such as Ilf3, Nop56, Nop58 and Noc2l, with combined scores of protein-protein interactions ranging from 0.573 to 0.924 (a value of 1 represents the highest possible relationship). Nevertheless, when analyzing the proteins from the downregulated DE genes, three clusters of relationships were observed and related to: 1) respiratory electron transport (Uqcrc2 and Etfa; combined score: 0.969), 2) amino acid degradation (Bckdha and Ivd; combined score: 0.915), and 3) glutathione-mediated detoxification pathway (Gstk1 and Ggh; combined score: 0.899).

### Transcriptome of juvenile testes of sustained fast-growing vs. sustained slow-growing fish (group FF vs. group SS comparison)

There were 113 DE genes when comparing the testis of FF and SS groups (43 up- and 70 downregulated genes; see Table S6 for a detailed list). A heatmap visualization of the data (Figure 3B) clearly separated individuals according to their group of origin.

The three most regulated GO terms in the BP category were related to metabolic processes and response to stimulus, followed by developmental process in the upregulated GO terms, and related to signaling for the downregulated GO terms (Figure 2A and 2B, respectively).

DAVID analysis of the DE genes yielded seven up- and 20 downregulated gene clusters mainly related to the Rps and Rpl ribosomal protein families. KEGG analysis showed twelve altered pathways: three that were upregulated in the FF fish and showed an opposite behavior to what had been observed for the F vs. S comparison (for example, the drug metabolism and the xenobiotics and glutathione metabolisms). There were also nine downregulated pathways related to accelerated growth and metabolism (see Table S7 for a detailed list of the pathways). Although low representation of sequences was found for each pathway, after a FDR test, ribosome was the most enriched pathway among both the up- and downregulated pathways, while proteasome was also highly enriched among the downregulated DE genes.

A Fisher's Exact Test with Multiple Testing Correction of FDR analysis of the most specific terms showed that eight biological processes, three molecular functions and three cell components GO terms were over-represented when using the whole microarray as a background (see Table S8 for further details). Most of the GO terms were related to the ribosome structure and the translation process.

**Figure 4:**
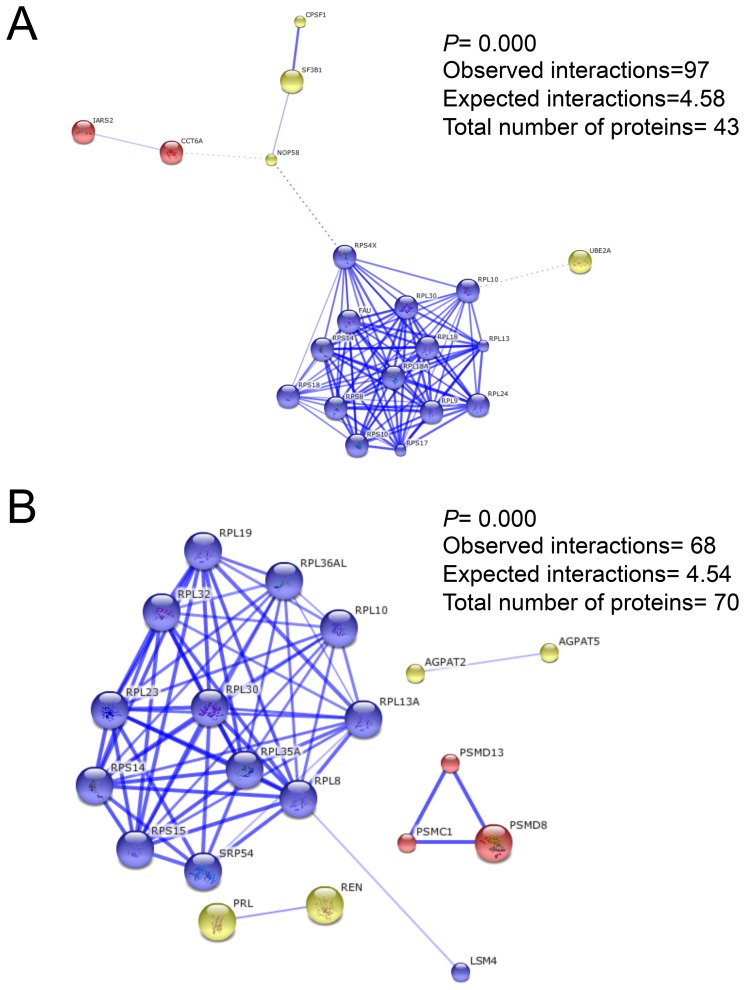
Protein-protein predicted confidence interactions for the FF vs. SS group comparison. A) The 43 proteins from the upregulated DE genes. B) The 70 proteins from the downregulated DE genes.

Protein-protein interaction analysis showed that upregulated proteins clustered in three different groups, where the largest one was related to the Rps (six different Rps) and Rpl (seven different Rpl) ribosomal protein families. These groups of proteins are found at the small and large ribosomal subunits, respectively (combined scores ranging from 0.401 to 0.999; Figure 4A). The other two clusters were conformed by the Iars2 and Cct6a proteins, which are related to translation and folding, as well as the 60S ribosomal subunit biogenesis-related proteins (Ube2a, Nop58, Sf3b1 and Cpsf1). On the other hand, downregulated protein interactions clustered in four groups, being the largest formed by the Rpl protein family (nine different Rpl proteins), but also forming part of the small ribosomal subunit and of the proteasome accessory complex (Figure 4B). The other three clusters were conformed by: 1) Agpat2 and Agpat5, which are involved in phospholipid metabolism; 2) Psmd13, Psmd8 and Psmc1, which are involved in ubiquitinated protein degradation; and 3) Prl and Ren, which are mainly involved in growth regulation and apoptosis.

### The effects of accelerating growth: Transcriptome of juvenile testes of growth-accelerated fish vs. sustained slow-growing fish (group SF vs. group SS comparison)

**Figure 5:**
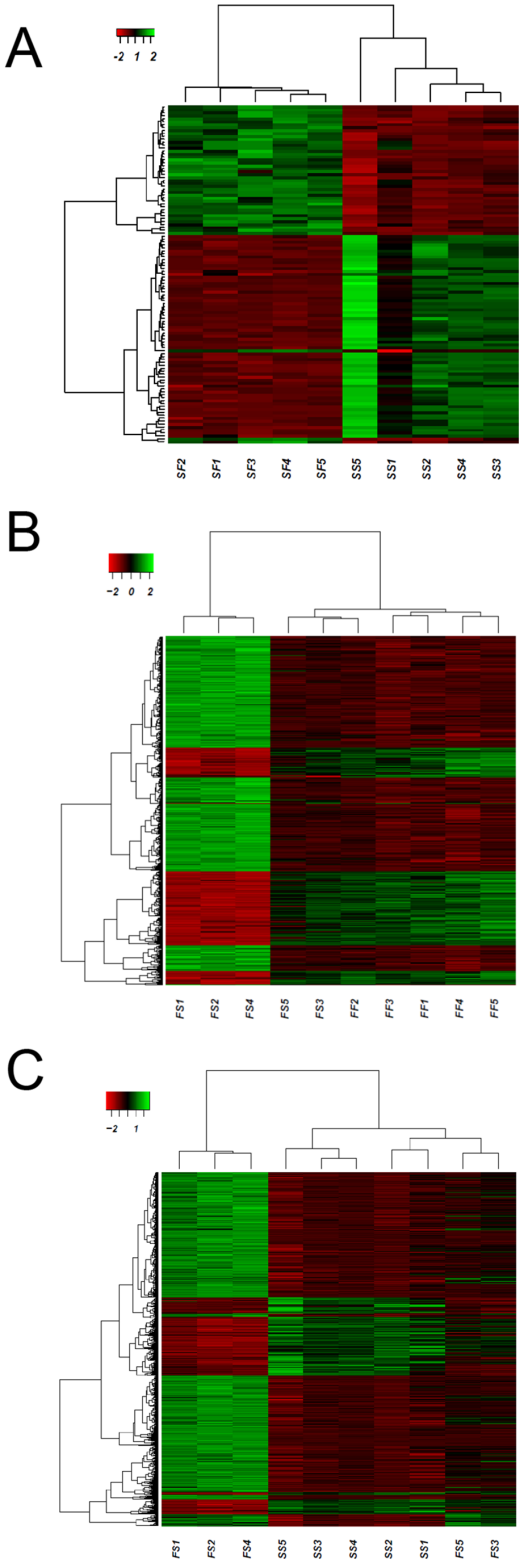
Individual heatmap representation of the transcriptome analysis of European sea bass one-year old testis. A) SF vs. SS comparison. B) FS vs. FF comparison. C) FS vs. SS comparison. Only DE genes are represented in the figure. High to low expression is shown by a degradation color from green to red, respectively. The scale bar shows Z-score values for the heatmap.

Despite significant differences (*P*<0.01) in SL and BW in favor of fish from group SF when compared to fish of the SS group (Table S1), the two groups had a similar sex ratio with a clear male bias (90.6 and 92.2% males, respectively; reported in [19]). However, the transcriptional comparison of the SF vs. the SS group had a low or moderate number of DE genes in the testis transcriptome. A heatmap analysis (Figure 5A) visually representing the 63 DE genes, 26 up- and 37 downregulated genes (see Table S9 for further details), showed that these two groups clustered separately.

BP subcategories were analyzed for the up- and downregulated GO terms (Figure 2A and 2B, respectively). Metabolic process GO terms were the most upregulated and contained five genes that were mainly related to amino acid metabolism (*ren, psme1, psmc1, trdmt1* and *agpat5*). However, renin and prolactin (*prl*), genes involved in positive regulation of growth, male gonad development, response to hormone stimulus and signaling (hormone-mediated or through G-protein coupled receptors) were downregulated.

DAVID analysis of the data with the highest stringency showed seven clusters for the upregulated genes (enrichment score of 23.68 to 0.42), being protein biosynthesis and translational elongation the most enriched ones. Among the downregulated genes, four clusters (enrichment scores from 1.48 to 0.06) were present and mostly related to peptidase activity. KEGG analysis showed 16 pathways altered due to the growth acceleration (three up- and twelve downregulated KEGG pathways; Table S10) that were mostly related to amino acids, glutathione and lipid metabolism. Fisher's Exact Test with Multiple Testing Correction of FDR of the most specific terms showed eight biological processes, three molecular functions and two cellular components that were over-represented when compared against our microarray background and were mainly related to ribosome structure and translational elongation and termination (Table S11).

Protein-protein interaction analysis with an MLC clustering method showed proteins from the upregulated DE genes clustering together and enriched in interactions (*P*<0.001). These proteins were either ribosomal (Rpl and Rps ribosomal protein families) or ribosome associated proteins (e.g., Ef1a1, Ubc or Fau). On the contrary, proteins from the downregulated DE genes were not enriched in interactions (*P* = 0.067) since just one interaction was present between Prl and Cort proteins (data not shown), which are known to be involved in growth control and signaling.

### The effects of accelerating growth: Transcriptome of juvenile testes of growth-accelerated fish vs. sustained fast-growing fish (group SF vs. group FF comparison)

Transcriptional analysis of the SF vs. FF group returned zero DE (Table 1) even when we looked for genes with a lower *P*-value (0.05) and lower FC (1.2). The two groups had different sex ratios (90.6% and 67.6% males, respectively) due to their initially different growth rates before size-grading, and FF fish were bigger in BW but not in SL at the time of sampling. However, from a transcriptional point of view, they had no differences, indicating a full recovery from the early naturally slow growth rates.

### The effects of decelerating growth: Transcriptome of juvenile testes of growth-decelerated fish vs. sustained fast-growing fish (group FS vs. group FF comparison)

Fish that experienced the same initial fast-growing rate also had a similar sex ratio (67.6% and 61.4% males, respectively) when compared to the S-derived groups (*P*<0.001), which were highly male-biased (>90%). However, when comparing growth between decelerated fish (FS) vs. sustained fast-growing fish (FF) there were differences in the final growth due to the different feeding regimes (FF>FS in SL and BW) during the sex differentiation period.

Differences at the transcriptomic level were found (469 DE genes: 316 up- and 153 downregulated genes; Table S12). A heatmap visualization of the data (Figure 5B), showed that two FS individuals (FS3 and FS5) shared a transcriptomic pattern with those of the FF group.

The three most regulated GO terms in the BP category were related to metabolic processes, response to stimulus and developmental process in the upregulated GO terms while signaling was for the downregulated subcategory (Figure 2A and 2B, respectively).

DAVID analysis showed 37 clusters from the upregulated genes (enrichment scores from 3.66 to 0.07) and functions were mainly related to proteolysis, regulation of ubiquitin, proteasome and protein modifications processes. On the contrary, downregulated genes (37 clusters; enrichment score from 1.82 to 0.0) had functions mostly related to biosynthesis of phospho- and glycerolipids, anabolic processes and RNA processing and splicing. These DE genes were part of 56 affected pathways (41 upregulated and 15 downregulated; Table S13). Upregulated pathways were the most altered ones after filtering for high stringency and were related to pyrimidine metabolism (*P*<0.001), RNA polymerase (*P*<0.05), oxidative phosphorylation (*P*<0.05), terpenoid backbone biosynthesis (*P*<0.05), epithelial cell signaling (*P*<0.05), purine metabolism (*P*<0.05), glutathione metabolism (*P*<0.05), glycosylphophatidylinositol (GPI)-anchor biosynthesis (*P*<0.05). With this high stringency filtering criteria, only proteasome (*P*<0.001) and ubiquitin mediated proteolysis (*P*<0.05) appeared as being affected among the downregulated pathways.

The Fisher's Exact Test with Multiple Testing Correction of FDR of the most specific terms showed twelve biological processes, eight molecular functions and three cellular components that were over-represented when compared against our microarray as a background and were related to mitochondria and transport activity, while receptor activity was found under-represented (Table S14).

The protein-protein interaction analysis showed that proteins corresponding to both DE up- (four different clusters; Figure S1) and downregulated (ten different clusters; Figure S2) genes were enriched in interactions (*P*<0.001). Upregulated protein clusters were conformed by: 1) proteasome-related proteins (e.g., Psma, Cct6a, Skp1 or Ube2v2), 2) signaling and cholesterol storage-proteins (e.g., Dmd, Mtor or Lpl), 3) transcription regulator proteins (e.g., Max, Pdcd10 or Itgb4), and 4) mitochondrial membrane respiratory chain (e.g., Mt-co1, Mt-nd1 or Mt-nd4). Downregulated proteins clustered in ten different groups but containing a few proteins, with the most enriched ones playing a role in: 1) signaling and protein degradation (e.g., Mapk14, Htra2), 2) translation initiation and protein folding (e.g., Eif4g1, Pdfn1) or 3) transcription (e.g., Polr2h, Gtf3a).

### The effects of decelerating growth: Transcriptome of juvenile testes of growth-decelerated fish vs. sustained slow-growing fish (group FS vs. group SS comparison)

The analysis of testes from fish that suffered from a growth deceleration during the sex differentiating period (FS) compared to fish with a sustained slow growth (SS group) showed differences (*P*<0.001) in the final sex ratio (61.4% and 92.2%, respectively) and in the final growth (FS>SS for both SL and BW). These results were further corroborated by the large transcriptomic differences found (978 DE genes: 717 up and 261 downregulated genes; Table S15). Heatmap visualization of data (Figure 5C) showed that two fish from the FS group (FS3 and FS5) clustered with the SS fish.

Analysis of the BP subcategories from the up- and downregulated GO terms (Figure 2A and 2B, respectively) showed how decelerating growth caused the highest changes in all subcategories. None of the genes from these GO terms of the FS vs. SS comparison coincided with those of the other decelerating comparison (FS vs. FF).

Further analysis of the data revealed that these 978 DE genes classified in 71 altered KEGG pathways (62 up- and nine downregulated), and were mostly related to RNA translation and elongation (Table S16). Moreover, clustering analysis of these genes with the highest stringency yielded 82 clusters for the up- and 48 clusters for the downregulated genes. The most enriched upregulated gene clusters were mainly related to the peroxisome, RNA splicing or nucleotide biosynthetic process, while the most enriched downregulated clusters were mostly related to protein catabolism processes, DNA modifications such as methylation, and response to nutrients.

A Fisher's Exact Test with multiple corrections for FDR of the most specific terms gave two BP, one MF and three CC GO terms that were over-represented when comparing to our custom microarray. These GO terms were mainly related to the ribosome structure and translation (Table S17). On the other hand, there was one GO term under-represented and related to the regulation of the immune system.

The highest representation of protein-protein interactions for this comparison (FS vs. SS) showed after a MLC clustering an enrichment in interactions (*P*<0.001), and presented six clusters for the upregulated proteins (Figure S3) related to: 1) ribosomal protein families (Rpl and Rps), 2) post-replication repair of damaged DNA and proteasome (e.g., Rad18 and Psm6, respectively), 3) response to stress (e.g., Tp53, Apex1, Ing1), 4) 60S ribosomal biogenesis and mRNA synthesis (e.g., Nop58, Nop16, Polr2f) and 5) respiratory chain (e.g., Ndufa1, Nduf53, Atp5g1). The twelve clusters for the downregulated proteins (Figure S4) were mainly related to: 1) regulation of metabolic pathways and chromosome stability (e.g., Csnk2b, Mapre1, Tubgcp2), 2) translation initiation (e.g., Eif4a2, Eif4e, Eif3d) and 3) regulation of cell responses (e.g., Prl, Irf1, Wipi1).

**Figure 6:**
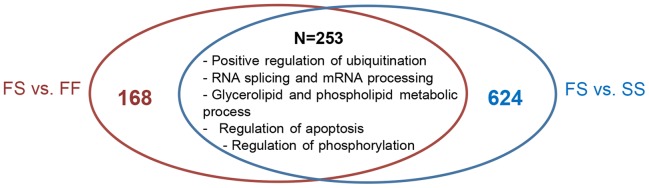
Venn diagram analysis of the DE genes by comparing (FS vs. FF) vs. (FS vs. SS). N represents the total number of common genes and the main categories in which genes are clustered.

The comparison between fast- and slow-growing groups vs. the FS group, a group that shows high transcriptomic activity but still some transcriptomic similarities with both groups (FF and SS), showed 253 shared DE genes (Figure 6). These were mainly grouped by five main functions: positive regulation of ubiquitination, RNA splicing and mRNA processing, regulation of apoptosis, glycerolipid and phospholipid metabolic process, and regulation of phosphorylation. Among these common 253 DE genes, there were just five genes that showed an opposite pattern of expression: *atf4, prelid1, rps17, psma6* and *dmd*, which were more expressed in the F-derived groups (FF>FS>SS) and mainly related to the proteasome complex and ribosomal structure. Renin (*ren*) and prolactin (*prl*), two genes involved in the positive regulation of growth, growth hormone and G-protein coupled receptor signaling pathways as well as in male gonad development were downregulated in both comparisons (FS vs. SS and FF; Figure 6) with a low expression of these genes in the initial fast-growing groups. This inhibition was also observed in fish with forced accelerated growth when compared to the slow-growing fish (SF vs. SS). These results indicated that these pathways are inhibited when the food availability is altered. Regarding the signaling function, apart from *ren* and *prl*, there were four coincident genes with the same pattern of expression in both comparisons (FS vs. SS and FF). Two of them, *atf4* and *ppkp2*, were upregulated in the FS group and involved in unfolded protein response and cell-cell signaling, while the other two, *errb* and *fkbp14*, were downregulated in the FS group and involved in steroid hormone- mediated signaling pathway and also in unfolded protein response.

## Discussion

The relationship between growth and sex has long been known for the European sea bass at the time of gonadal differentiation, where the largest fish are essentially all females whereas both sexes are found among the smallest fish, although males predominate. Early size-grading experiments (between 66–143 dph) have confirmed this [13], [15], [17] by obtaining ∼90% females among the largest selected fish. Moreover, in a previous study we found that altering growth rates during the sex differentiation period in both size-graded and non-size-graded populations did not alter the sex ratios [19]. Here, it is presented for the first time a microarray analysis of undifferentiated gonads from 4-month-old sea bass with opposite growing rates just after size-grading (T1), and on differentiated testis (11-month-old juveniles, T2). To the best of our knowledge, the question of whether naturally occurring differences in somatic growth are somehow translated in observed transcriptomic differences in the gonads during sex differentiation has never been explored in fish. Nevertheless, what could be called a related type of work was performed in mitten crabs [36], where it was separately analyzed the relationship between nutrition and reproduction, by examining the hepatopancreas and testes transcriptomes, respectively. Interestingly, and regardless the differences among experimental designs and the model organisms used, some traits found in the study with crabs, as the differential expression of some heat shock proteins, cell death suppressors, RNA-dependent DNA polymerases or controllers of splicing, were also found in our study. Similarly to what has been previously reported in fish liver and muscle transcriptomes [22], [24], [26], [52], it is then clear that the juvenile testis is also affected by changes in food supply. Interestingly, there are then common transcriptomic responses with the above mentioned tissues, but not with the brain transcriptomic responses [52].

### Fast growing vs. slow growing fish before the onset of SD

Differences between naturally fast- vs. slow-growing (F vs. S) European sea bass of the same family early in development (T1) were not reflected in major transcriptomic changes in their undifferentiated gonads since only 40 DE genes were found. Translation was an active process in F fish gonads since genes coding for ribosomal structure, protein translation and folding were highly expressed. Immune response (positive regulation of apoptosis) and reproduction-related pathways were upregulated, although the only gene directly related to reproduction, the tyrosine-protein kinase-like (*ptk*), which is associated with male gonad development function, is also present in other biological functions like cytoskeleton reorganization or cell proliferation. In contrast, gonads from fish that showed the slowest growth before size-grading (group S) were undergoing catabolism processes and protein recycling, since pathways related to negative regulation of growth, protein and amino acid catabolism, protein modification and fatty acid biosynthesis were highly expressed. The lack of abundance in reproduction-related genes among the DE genes present in the F vs. S comparison may be because the custom-made microarray used in this study was more enriched for immunology and growth-related terms rather than for reproduction related terms as it was based on the availability of the public sequences at the time. However, as can be seen in the results of this study, essentially most of the important genes related to sex differentiation found in this and other piscine species are present in this array, which ensures that it fulfills the requirements for such a type of study.

These results indicate that large intrafamily differences in somatic growth within a group of 4-month-old European sea bass due to natural variation are not necessarily translated in a large number of DE genes in their sexually undifferentiated gonads. This is relevant because occurs despite the fact that the groups made selecting the largest fish contain more future females than the groups made selecting the smallest fish, as shown before [13], [15], [17], and as evidenced by actual subsequent differences in the sex ratios of these groups, since the number of females in the F-derived groups was ∼40% while in the S-derived groups it was only ∼10% [19]. Thus, there is indeed a clear relationship between growth at the beginning of sex differentiation at 4 months and future sex ratio. Since prior to 4 months growth was not manipulated, growth and future sex can be related, but not food supply and future sex at 4 months. What is also not possible is to relate a given transcriptomic profile at the time of sex differentiation with future sex on an individual fish basis; that is impossible because in order to analyze the transcriptome the fish must be sacrificed. Changes at 4 months probably precede subsequent changes that would account for the differences in sex ratio between the F- and S-derived groups observed when juveniles.

### Effects of unrestricted growth on the juvenile testis transcriptome

Food intake is one of the main factors influencing growth rates in aquatic production [25] and food restriction is directly associated with reduced growth rates in fish including the European sea bass [19]. The lack of transcriptomic differences between the SF and FF groups highlights the balance between protein synthesis and degradation, i.e., protein turnover, as one of the most important active processes in the gonads. Protein turnover relies on proteins mainly obtained from the diet, since high protein contributions from diet or low protein turnover (catabolism) are translated into higher growth rates [53]. In fact, we found lower expression of genes related to catabolism in the accelerated growth group (SF) when compared to the group with sustained growth (FF). The genes related to protein turnover, together with genes involved in the immune system, were also downregulated in the juvenile testis, as it had been found before in the liver of Atlantic salmon (*Salmon salar*) fed with a supplemental diet [26], and it was suggested that this is so because they are involved in the regulation of the decrease in whole body metabolic demands, resulting in less energy wastage and an enhancement in growth performance [24].

Groups with unrestricted growth during the sex differentiation period (FF and SF) showed in common an increased expression of genes related to high protein translation and folding, mainly of proteins related to ribosome structure. This, together with the lack of histological differences between groups, shows how gonads from slow-growing fish can still recover after a period of slow growth if food supply is not a limiting factor thereafter. This is remarkable since it shows the plasticity of the gonads during the sex differentiation period to environmental effects, since fish present a capacity of exploiting a situation of food abundance and recover from initial slow growth.

### Effects of restricted food supply on the juvenile testis transcriptome

As also found in the Atlantic salmon liver [26] and white muscle transcriptome [24], [54] after food deprivation, protein synthesis and degradation decreased in European sea bass juvenile testes, since both processes are very demanding in terms of energy requirements [53]. It is known that defective or damaged proteins (proteolysis process) are constantly degraded by the proteasome following two main pathways: lysosome or ubiquitin-proteasome pathways [55], [56]. Our results show that in European sea bass gonads proteolysis was mainly achieved by the ubiquitin-proteasome pathway rather than the lysosome pathway, as described before in rainbow trout and gilthead sea bream, *Sparus aurata* [22], [54], [57], since we observed a larger representation of genes involved in the ubiquitin-proteasome pathway. The ubiquitin-proteasome pathway, mainly responsible for protein degradation, was downregulated in the group FS when compared to the group FF and contrasting with the SF and SS groups. Also, food supply restriction was accompanied by a downregulation of genes related to protein synthesis and degradation, and with the immune system, in agreement with previous observations made in the Atlantic salmon liver [26] and in white muscle [24] transcriptomes after food deprivation, although some genes of the complement system [58] were still upregulated. Moreover, a decrease in the lipoprotein levels, in transcription, in tRNA synthesis, and in protein synthesis and elongation in group FS, as a consequence of food deprivation, was coincident with previous results in fasted cod (*Gadus morhua*) as an energy-conserving mechanism [59], and is a common and strong downregulated response of the energy-generating processes in the adipose tissue [60]. This may be because during food deprivation hormonal signals such as growth hormone or insulin levels are translating food restriction signals into a protein turnover change by decreasing the protein synthesis and increasing catabolism [61] to save energy [1], [62].

Several studies in fish have analyzed the responses to starvation by measuring catch-up growth [63] or analyzing the effects on the muscle and liver transcriptomes [22], [64] but, to our knowledge, this is the first time that a similar study is performed in fish gonads. Two individuals from group FS (FS3 and FS5) clustered with individuals from the FF and SS groups, showing transcriptome similarities. This suggest that these FS fish still conserved some traits of the pre-T1 period but also traits related to the T1–T2 period when food supply was reduced, reflecting the existence of an adaptation to sudden feeding changes [1]. Most of these changes were related to protein turnover. This may be due to the adaptive capacity of fish to sense environmental fluctuations that in turn drive changes in their metabolism, since protein turnover is a highly energy-demanding process [24], [53].

The comparison of groups FS vs. FF or SS evidenced some common features of expression where processes such as apoptosis, ubiquitin catabolism, peroxisome, kinase activity or regulation of cellular growth were increased. On the other hand, processes such as proteolysis, regulation of protein modifications, RNA processing, regulation of transcription factors, chromatin assembly, response to nutrients or gamete generation and reproduction were decreased, opposite to what has been found for sea bream heart transcriptome, where transcription was enhanced and transcription inhibitors downregulated [22]. These observations indicate that FS fish had to cope with the dramatic reduction of food intake by saving energy at different levels [1], [19], [24], [26]. This is also supported by the fact that pathways related to metabolism such as lipid mobilization, or purine and pyrimidine metabolism were upregulated, as well as the pathways related to stress response such as the mTOR signaling pathway, which is involved in DNA damage and nutrient deprivation. In contrast, amino acid metabolism, xenobiotic removal or glucose metabolism were downregulated when comparing the FS to the FF and SS groups, showing how the naturally fast-growing fish fed with a non-restrictive diet and later subjected to food restriction did not obtain enough dietary energy and therefore had to start mobilizing lipids and activating gluconeogenesis. Moreover, in agreement with what has been found in both white and red muscle of gilthead sea bream under food restriction [22], mitochondria and ATP transport GO terms were enriched in juvenile testes, a fact that has been proposed as a link between food restriction and stress response mediated by cortisol [22].

No matter which one of the extreme groups the FS group was compared to, processes related to translation and protein regulation such as unfolded protein response, proteasome and postregulation of damaged DNA were highly active. Moreover, processes such as translation initiation, protein folding, transcription and cell-cell signaling were also taking place. Together, these results indicated that although food was scarce and growth was decelerated the transcriptional and the translational machineries of the testis were still active in the FS group. Furthermore, the steroid biosynthesis pathway was downregulated in the FS group when compared to the SF and SS groups, suggesting that the adaptation to the growth decrease could be affecting the energy dedicated to future gonad maturation, although without apparent major consequences, since there were no histological differences when fish were sampled. Reproduction-related processes such as steroid biosynthesis, steroid hormone-mediated signaling and cholesterol storage were affected by growth deceleration, since they were downregulated in the FS group when compared to FF group. However, the FS group showed an increase in GO terms related to spermatogenesis/male gamete generation. This suggests that the FS fish, although being the most different group from a transcriptomic point of view due to food restriction during the sex differentiation period, were still allocating some of the energy in preparation for gonad maturation, which in farmed European sea bass takes place during the second year of life.

## Conclusions

To the best of our knowledge, this is the first study evaluating the effects of different growth rates on gonadal development in fish with a transcriptomic approach.

The transcriptome of sexually-undifferentiated gonads was not drastically affected by initial natural differences in growth rates (fish from the opposite sides of the normal distribution curve). In addition, regardless the maturation status of the gonad (T1 and T2), as it has also been shown previously for liver and muscle, the slow-growing fish transcriptome showed an altered protein turnover with a higher catabolism, represented by a reduction in transcription and translation, a decreased immunological response, and a metabolism based on lipids and gluconeogenesis. On the other hand, the transcriptome of fast-growing fish reflects an enhancement of anabolic processes such as transcription, translation, protein synthesis and elongation and a metabolism based on glucose.

In differentiated juvenile gonads, the highest effects on the testis transcriptome were observed when forcing a naturally fast-growing fish to decelerate its growth through food restriction, since those fish showed high transcriptomic differences when compared to the sustained fast-growing fish and even more differences when compared to the fish with sustained slow-grow. These results suggest that food availability during the sex differentiation period is indeed able to modulate the testis transcriptome.

Interestingly, individuals with an initial slow grow but later with an accelerated growth due to increased food supply during sex differentiation showed a recovered transcriptome. These results suggest that fish are able to recover transcriptionally their testes if they are provided with enough food supply during the sex differentiation period. Nevertheless, the opposite is not true, since a natural initial fast growth does not ensure any advantage in terms of transcriptional fitness if later food becomes scarce. These results have implications for natural fish populations subjected to fluctuating food supply in a scenario of global change, as well as for populations or a part thereof of farmed fish under suboptimal feeding regimes, since they provide information on the possible consequences that these situations may have for the reproductive physiology of fish.

## Supporting Information

Figure S1 **Protein-protein predicted confidence interactions for the FS vs. FS group comparison.** The interactions of 266 proteins from the upregulated DE genes are shown. The expected and observed interactions are shown with the significance level.
(TIF)

Figure S2 **Protein-protein predicted confidence interactions for the FS vs. FF group comparison.** The interactions of 129 proteins from the downregulated DE genes are shown. The expected and observed interactions are shown with the significance level.
(TIF)

Figure S3 **Protein-protein predicted confidence interactions for the FS vs. SS group comparison.** The interactions of 602 proteins from the upregulated DE genes are shown. The expected and observed interactions are shown with the significance level.
(TIF)

Figure S4 **Protein-protein predicted confidence interactions for the FS vs. SS group comparison.** The interactions of 206 proteins from the downregulated DE genes are shown. The expected and observed interactions are shown with the significance level.
(TIF)

Table S1 Biometric data of the individuals used for the transcriptomic analysis.
(DOCX)

Table S2 Quantitative RT-PCR primer characteristics.
(DOCX)

Table S3 List of the number of GO terms found for each category for all the comparisons studied.
(DOCX)

Table S4 DE gene list for the F vs. S group comparison.
(DOCX)

Table S5 Affected KEGG pathways in the F vs. S group comparison.
(DOCX)

Table S6 DE gene list for the FF vs. SS group comparison.
(DOCX)

Table S7 Affected KEGG pathways in the FF vs. SS group comparison.
(DOCX)

Table S8 Two-tails Fisher's exact test with Multiple Testing Corrections of FDR results for the FF vs. SS group comparison.
(DOCX)

Table S9 DE gene list for the SF vs. SS group comparison.
(DOCX)

Table S10 Affected KEGG pathways in the SF vs. SS group comparison.
(DOCX)

Table S11 Two-tails Fisher's exact test with Multiple Testing Corrections of FDR results for the SF vs. SS group comparison.
(DOCX)

Table S12 DE gene list for the FS vs FF group comparison.
(DOCX)

Table S13 Affected KEGG pathways in the FS vs. FF group comparison.
(DOCX)

Table S14 Two-tails Fisher's exact test with Multiple Testing Corrections for FDR results for the FS vs. FF group comparison.
(DOCX)

Table S15 DE gene list for the FS vs. SS group comparison.
(DOCX)

Table S16 Affected KEGG pathways in the FS vs. SS group comparison.
(DOCX)

Table S17 Two-tails Fisher's exact test with Multiple Testing Correction for FDR results for the FS vs. SS group comparison.
(DOCX)
