# Supplementary figures and images for: Effects of Changes in Food Supply at the Time of Sex Differentiation on the Gonadal Transcriptome of Juvenile Fish. Implications for Natural and Farmed Populations
Source: PLoS One. 2014 Oct 23;9(10):e111304. doi: 10.1371/journal.pone.0111304 (PMC4207807; doi:10.1371/journal.pone.0111304)

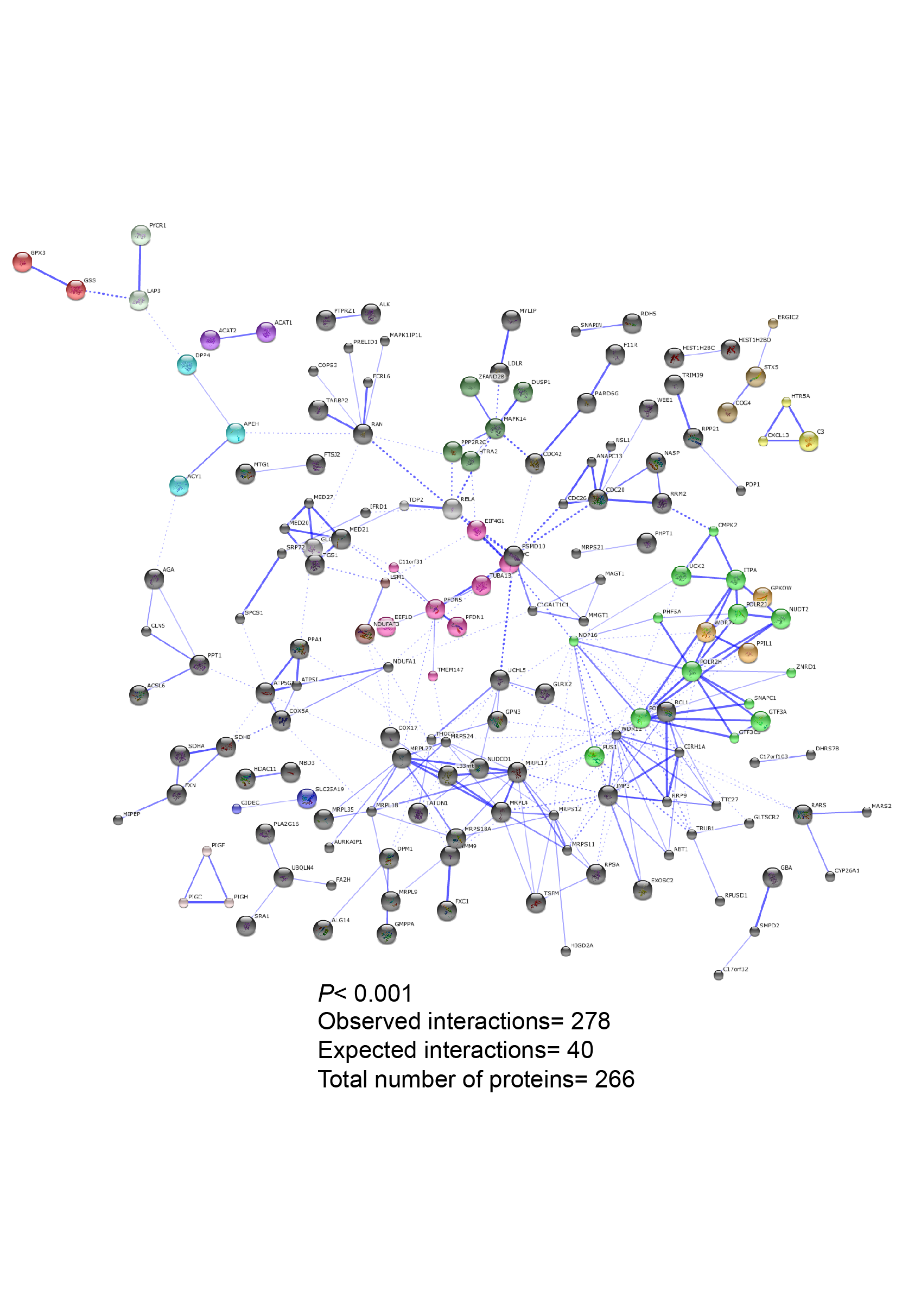

Supplement: Figure S1 — Protein-protein predicted confidence interactions for the FS vs. FS group comparison. The interactions of 266 proteins from the upregulated DE genes are shown. The expected and observed interactions are shown with the significance level. (TIF) [file pone.0111304.s001.tif]

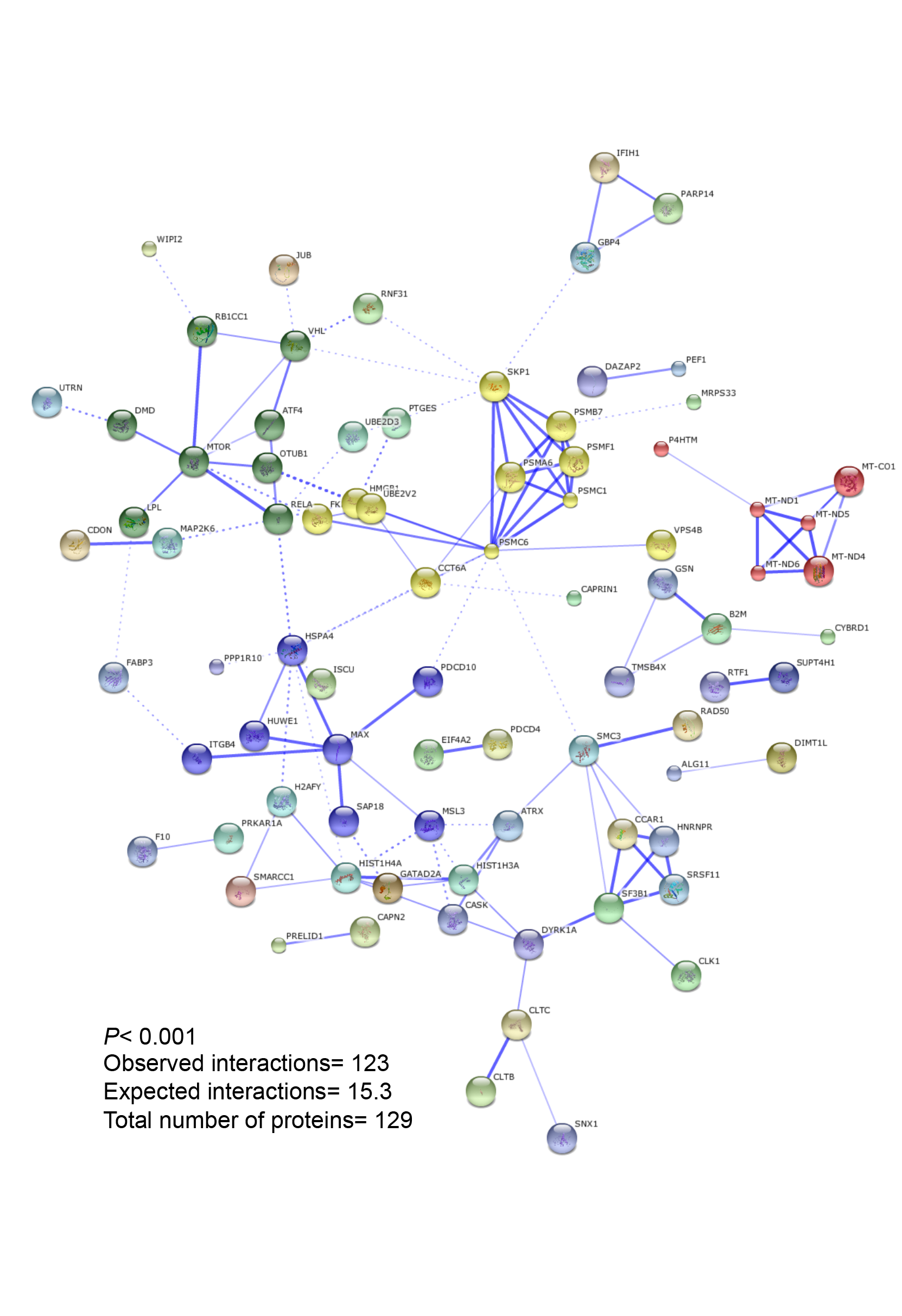

Supplement: Figure S2 — Protein-protein predicted confidence interactions for the FS vs. FF group comparison. The interactions of 129 proteins from the downregulated DE genes are shown. The expected and observed interactions are shown with the significance level. (TIF) [file pone.0111304.s002.tif]

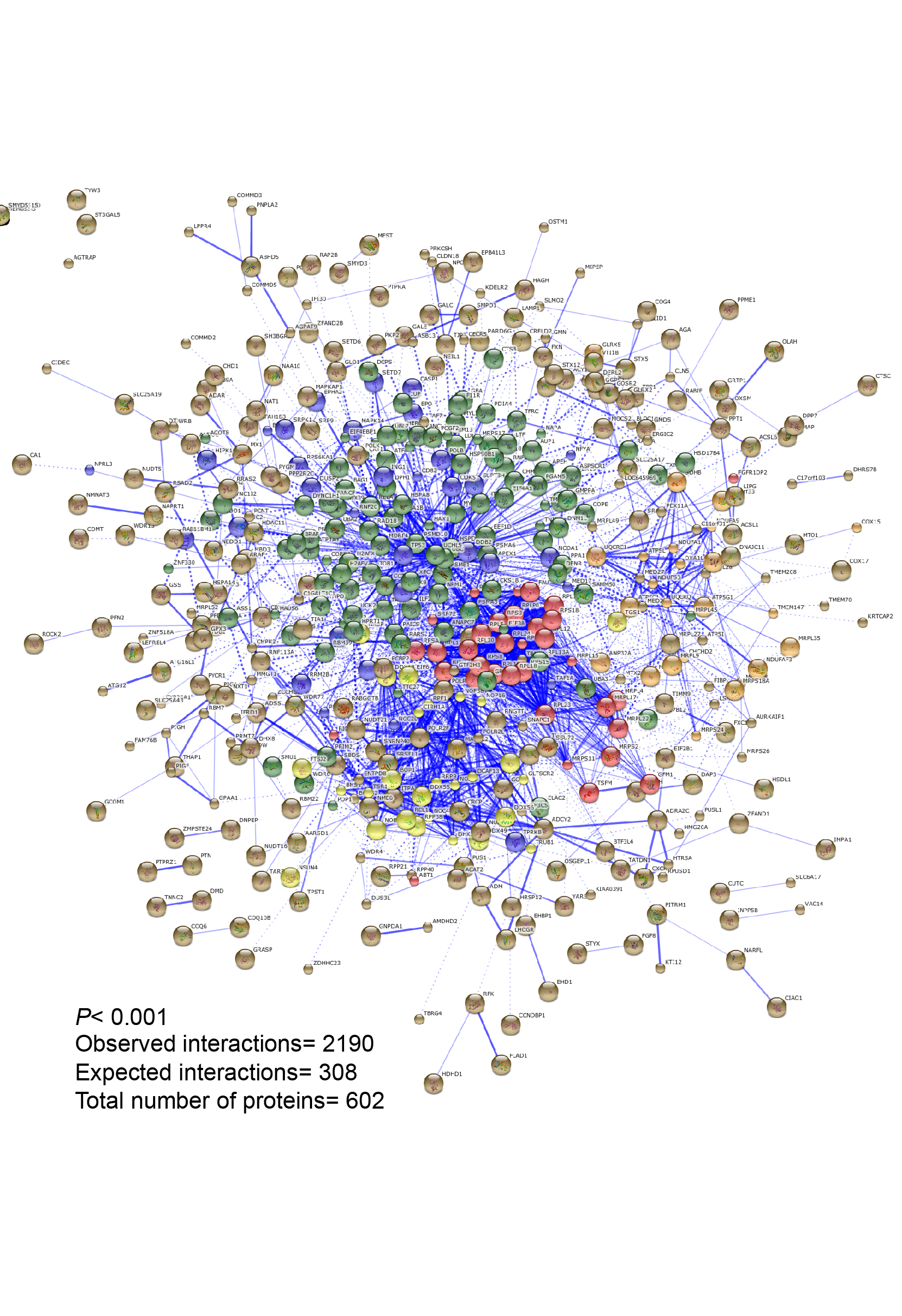

Supplement: Figure S3 — Protein-protein predicted confidence interactions for the FS vs. SS group comparison. The interactions of 602 proteins from the upregulated DE genes are shown. The expected and observed interactions are shown with the significance level. (TIF) [file pone.0111304.s003.tif]

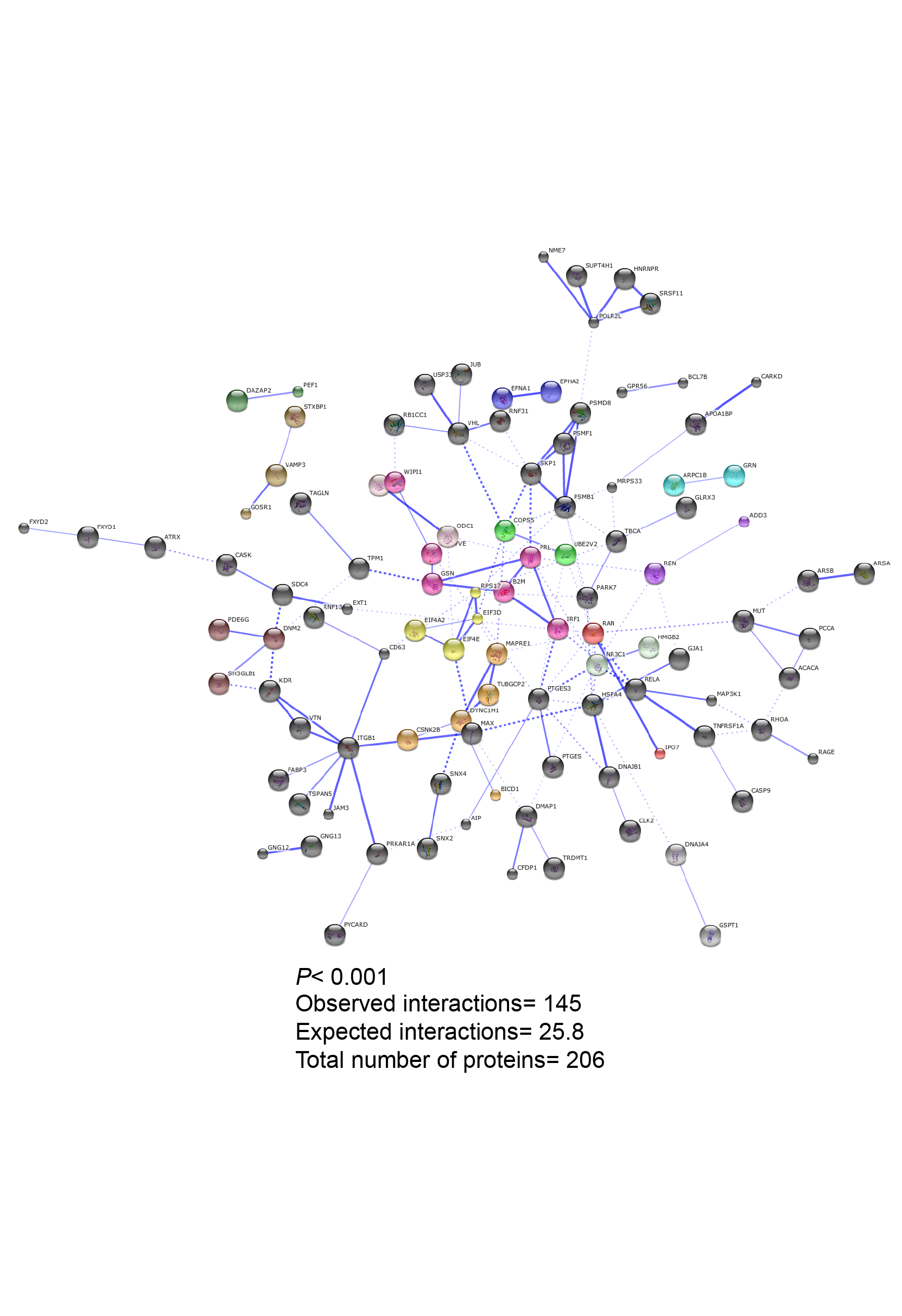

Supplement: Figure S4 — Protein-protein predicted confidence interactions for the FS vs. SS group comparison. The interactions of 206 proteins from the downregulated DE genes are shown. The expected and observed interactions are shown with the significance level. (TIF) [file pone.0111304.s004.tif]
