# Supplementary material for: Effects of Changes in Food Supply at the Time of Sex Differentiation on the Gonadal Transcriptome of Juvenile Fish. Implications for Natural and Farmed Populations
Source: PLoS One. 2014 Oct 23;9(10):e111304. doi: 10.1371/journal.pone.0111304 (PMC4207807; doi:10.1371/journal.pone.0111304)
Supplement: Table S1 — Biometric data of the individuals used for the transcriptomic analysis. (DOCX) [file pone.0111304.s005.docx]

Supplementary Table 1. Biometric data of the individuals used for the transcriptomic analysis

|  | N | SL (cm) | BW (g) |
| --- | --- | --- | --- |
| *Time 1* |  |  |  |
| Group F | 7 | 4.9±0.218** | 1.93±0.279 |
| Group S | 8 | 3.6±0.043 | 0.70±0.029 |
|  |  |  |  |
| *Time 2* |  |  |  |
| Group FF | 5 | 15.06±0.730^a^ | 60.20±4.609^a^ |
| Group SF | 5 | 13.56±0.256^ab^ | 45.07±2.134^b^ |
| Group FS | 5 | 14.14±0.564^a^ | 40.24±2.370^b^ |
| Group SS | 5 | 12.26±0.244^b^ | 28.07±1.105^c^ |

*Statistical differences (P<0.01) between Time 1 groups after runing a t-test.

Different letters mark statistical differences among Time 2 groups by an ANOVA analysis. Note that Group S BW did not follow a normal distribution and had to be log transformed prior to statistical analysis.
