# Supplementary material for: Effects of Changes in Food Supply at the Time of Sex Differentiation on the Gonadal Transcriptome of Juvenile Fish. Implications for Natural and Farmed Populations
Source: PLoS One. 2014 Oct 23;9(10):e111304. doi: 10.1371/journal.pone.0111304 (PMC4207807; doi:10.1371/journal.pone.0111304)
Supplement: Table S2 — Quantitative RT-PCR primer characteristics. (DOCX) [file pone.0111304.s006.docx]

Supplementary Table 2. Quantitative RT-PCR primer characteristics

| Comparison | Genes | Gene abbrev. | Primer name | Primer sequence (5’→3’) | Efficiency (E) | R^2^ | Fold change | adjusted *P*-value |
| --- | --- | --- | --- | --- | --- | --- | --- | --- |
|  |  |  |  |  |  |  |  |  |
| F vs. S | filamin-A | *Flna* | flna-Fw | GTGCTCCAGGTCTGTGTCCT | 1.96 | 0.96 | 2.849 | 0.004 |
|  |  |  | flna-Rev | TTGGGGTCAACGATCTCTTC |  |  |  |  |
|  | tetraspanin 1 | *tspan1* | tspan1-Fw | CAAATCCTGGAGACCCTCAA | 1.8 | 0.96 | -5.899 | 0.007 |
|  |  |  | tspan1-Rev | GTCCAGGTGGCAGTACAGGT |  |  |  |  |
|  |  |  |  |  |  |  |  |  |
| FF vs. SS | T-complex protein 1 subunit zeta | *cct6a* | cct6a-Fw | TTACTTCGCTGGTTGTGCTG | 2.14 | 0.97 | 2.335 | 0.001 |
|  |  |  | cct6a-Rev | CGGTCCCAGGTTACTTTTCA |  |  |  |  |
|  | 40S ribosomal protein S15 | *rps15* | rps15-Fw | TGGCGGATACCGAGATTAAG | 2,00 | 0.99 | -13.276 | 0.000 |
|  |  |  | rps15-Rev | CTGCATCAGCTGCTCATAGG |  |  |  |  |
|  |  |  |  |  |  |  |  |  |
| FSvs. FF | fatty acid-binding protein, heart | *fabp3* | fabp3-Fw | CCACCACCATCATCTCAGTG | 2.03 | 0.97 | 15.341 | 0.007 |
|  |  |  | fabp3-Rev | CGTCAAACTCCTCTCCAAGC |  |  |  |  |
|  | geranylgeranyl pyrophosphate synthase | *ggps1* | ggps1-Fw | TCAAACAGCTCAGCCAAATG | 2.03 | 0.97 | -11.577 | 0.006 |
|  |  |  | ggps1-Rev | TGTTTTAGCGGAGTGTGCAG |  |  |  |  |
|  |  |  |  |  |  |  |  |  |
| SF vs. SS | ribosomal protein L9 | *rpl9* | rpl19-Fw | CGTCCTTCCTTCCTCCTTTC | 2.2 | 0.92 | 2.606 | 0.001 |
|  |  |  | rpl19-Rev | CACAATACTGTCCGCACCTG |  |  |  |  |
|  | propionyl-CoA carboxylase alpha chain, mitochondrial | *Pcca* | pcca-Fw | CACACAGGCTCTCTCCATCA | 1.96 | 0.98 | -14.035 | 2.452 |
|  |  |  | pcca-Rev | TGGTGTTGTAGACGGTGGAA |  |  |  |  |
|  |  |  |  |  |  |  |  |  |
| FS vs. SS | carbonic anhydrase | *ca1* | ca1-Fw | TGCCATAGTTGCTAACGCAC | 1.97 | 0.98 | 36.701 | 0.004 |
|  |  |  | ca1-Rev | CTCATGGGACAGCCCTAACA |  |  |  |  |
|  | 1-acyl-sn-glycerol-3-phosphate acyltransferase epsilon | *agpat5* | agpat5-Fw | GCTGACTGGATCATTGCTGA | 1.95 | 0.97 | -13.376 | 7.796 |
|  |  |  | agpat5-Rev | TCCTCCGTGCTGAGAGAAAT |  |  |  |  |
