# Supplementary material for: Effects of Changes in Food Supply at the Time of Sex Differentiation on the Gonadal Transcriptome of Juvenile Fish. Implications for Natural and Farmed Populations
Source: PLoS One. 2014 Oct 23;9(10):e111304. doi: 10.1371/journal.pone.0111304 (PMC4207807; doi:10.1371/journal.pone.0111304)
Supplement: Table S3 — List of the number of GO terms found for each category for all the comparisons studied. (DOCX) [file pone.0111304.s007.docx]

Supplementary Table 3. List of number of GO terms found for each category for all the comparisons studied.

|  | F vs. S | FF vs. SS | SF vs. SS | FS vs. FF | FS vs. SS |
| --- | --- | --- | --- | --- | --- |
| *Upregulated GO terms* |  |  |  |  |  |
| **Biological process** |  |  |  |  |  |
| Single-organism process | 12 | 27 | 22 | 76 | 367 |
| Signaling | 7 | 7 | 4 | 43 | 131 |
| Rhythmic process | 0 | 0 | 0 | 0 | 0 |
| Response to stimulus | 10 | 10 | 7 | 61 | 191 |
| Reproduction | 1 | 2 | 2 | 0 | 27 |
| Multi-organism process | 2 | 9 | 14 | 19 | 67 |
| Multicellular organismal process | 6 | 9 | 6 | 45 | 143 |
| Metabolic process | 10 | 34 | 25 | 101 | 449 |
| Locomotion | 3 | 3 | 2 | 0 | 25 |
| Localization | 7 | 15 | 15 | 32 | 178 |
| Immune system process | 3 | 6 | 5 | 21 | 62 |
| Growth | 2 | 1 | 50 | 0 | 26 |
| Developmental process | 7 | 8 | 5 | 39 | 119 |
| Cellular process | 16 | 35 | 26 | 103 | 507 |
| Cellular component organization or biogenesis | 9 | 15 | 17 | 40 | 202 |
| Cell killing | 0 | 0 | 0 | 0 | 0 |
| Biological regulation | 12 | 19 | 14 | 70 | 287 |
| Biological adhesion | 1 | 2 | 1 | 0 | 15 |
| Cellular component | 0 | 0 | 0 | 0 | 0 |
| **Molecular funcion** |  |  |  |  |  |
| Transporter activity | 1 | 2 | 1 | 0 | 32 |
| Translation regulator activity | 0 | 2 | 2 | 0 | 0 |
| Structural molecule activity | 2 | 14 | 17 | 0 | 55 |
| Receptor activity | 0 | 0 | 1 | 0 | 14 |
| Protein binding transcription factor activity | 1 | 0 | 0 | 0 | 20 |
| Nucleic acid binding transcription factor activity | 0 | 3 | 1 | 11 | 15 |
| Molecular transducer activity | 2 | 1 | 1 | 0 | 15 |
| Enzyme regulator activity | 0 | 2 | 1 | 0 | 23 |
| Electron carrier activity | 0 | 0 | 0 | 0 | 0 |
| Chemoattractant activity | 0 | 0 | 0 | 0 | 0 |
| Channel regulator activity | 0 | 1 | 1 | 0 | 0 |
| Catalytic activity | 4 | 11 | 8 | 60 | 283 |
| Binding | 15 | 25 | 18 | 107 | 453 |
| Antioxidant activity | 0 | 0 | 0 | 0 | 0 |
| **Cellular component** |  |  |  |  |  |
| Virion | 0 | 0 | 0 | 0 | 0 |
| Synapse | 1 | 0 | 0 | 0 | 0 |
| Organelle | 15 | 29 | 25 | 86 | 450 |
| Nucleoid | 0 | 1 | 0 | 0 | 0 |
| Membrane-enclosed lumen | 5 | 12 | 6 | 40 | 173 |
| Membrane | 8 | 8 | 7 | 42 | 211 |
| Macromolecular complex | 10 | 24 | 23 | 55 | 253 |
| Extracellular region | 3 | 0 | 1 | 16 | 33 |
| Extracellular matrix | 0 | 0 | 1 | 0 | 0 |
| Cell junction | 0 | 2 | 1 | 0 | 20 |
| Cell | 16 | 34 | 26 | 103 | 518 |
| *Downregulated GO terms* |  |  |  |  |  |
| **Biological process** |  |  |  |  |  |
| Single-organism process | 8 | 42 | 18 | 136 | 140 |
| Signaling | 5 | 18 | 12 | 44 | 82 |
| Rhythmic process | 0 | 2 | 2 | 0 | 0 |
| Response to stimulus | 7 | 25 | 15 | 60 | 99 |
| Reproduction | 2 | 3 | 4 | 0 | 16 |
| Multi-organism process | 2 | 12 | 5 | 20 | 21 |
| Multicellular organismal process | 4 | 20 | 10 | 53 | 81 |
| Metabolic process | 10 | 43 | 22 | 169 | 132 |
| Locomotion | 1 | 4 | 2 | 0 | 19 |
| Localization | 6 | 21 | 6 | 54 | 75 |
| Immune system process | 1 | 5 | 4 | 21 | 25 |
| Growth | 2 | 5 | 3 | 0 | 14 |
| Developmental process | 4 | 13 | 9 | 47 | 61 |
| Cellular process | 10 | 52 | 26 | 194 | 168 |
| Cellular component organization or biogenesis | 3 | 21 | 11 | 80 | 73 |
| Cell killing | 0 | 1 | 1 | 0 | 0 |
| Biological regulation | 6 | 29 | 17 | 101 | 124 |
| Biological adhesion | 1 | 7 | 4 | 0 | 21 |
| **Molecular function** |  |  |  |  |  |
| Transporter activity | 2 | 8 | 2 | 14 | 19 |
| Structural molecule activity | 1 | 9 | 0 | 15 | 11 |
| Receptor regulator activity | 0 | 0 | 0 | 0 | 0 |
| Receptor activity | 0 | 4 | 1 | 0 | 19 |
| Protein binding transcription factor activity | 0 | 3 | 1 | 11 | 0 |
| Nucleic acid binding transcription factor activity | 1 | 4 | 2 | 0 | 12 |
| Molecular transducer activity | 1 | 4 | 0 | 0 | 20 |
| Metallochaperone activity | 0 | 0 | 0 | 0 | 0 |
| Enzyme regulator activity | 0 | 2 | 1 | 0 | 12 |
| Electron carrier activity | 1 | 1 | 0 | 0 | 0 |
| Chemoattractant activity | 0 | 0 | 0 | 0 | 0 |
| Channel activity | 0 | 0 | 0 | 0 | 0 |
| Catalytic activity | 5 | 26 | 15 | 110 | 85 |
| Binding | 12 | 45 | 26 | 170 | 158 |
| Antioxidant activity | 0 | 0 | 0 | 0 | 0 |
| **Cellular component** |  |  |  |  |  |
| Synapse | 0 | 0 | 1 | 0 | 0 |
| Organelle | 10 | 37 | 20 | 169 | 126 |
| Membrane-enclosed lumen | 3 | 12 | 10 | 76 | 46 |
| Membrane | 9 | 17 | 11 | 93 | 99 |
| Macromolecular complex | 3 | 24 | 9 | 100 | 87 |
| Extracellular region | 2 | 7 | 5 | 15 | 25 |
| Extracellular matrix | 0 | 2 | 1 | 0 | 0 |
| Cell junction | 0 | 2 | 0 | 0 | 13 |
| Cell | 14 | 50 | 25 | 198 | 166 |
