# Supplementary material for: Effects of Changes in Food Supply at the Time of Sex Differentiation on the Gonadal Transcriptome of Juvenile Fish. Implications for Natural and Farmed Populations
Source: PLoS One. 2014 Oct 23;9(10):e111304. doi: 10.1371/journal.pone.0111304 (PMC4207807; doi:10.1371/journal.pone.0111304)
Supplement: Table S4 — DE gene list for the F vs. S group comparison. (DOCX) [file pone.0111304.s008.docx]

Supplementary Table 4. Differentially expressed gene list. Fast versus Slow comparison

| Description | Gene symbol | Fold change | Adjusted *P*-value |
| --- | --- | --- | --- |
| protein kinase ORF73 | *orf73* | 2.859 | 0.007 |
| filamin-A | *flna* | 2.849 | 0.004 |
| arachidonate 12-lipoxygenase | *alox12* | 2.407 | 0.002 |
| Gelsolin | *gsn* | 2.192 | 0.010 |
| hypothetical YFW family protein 1 | *yfw1* | 2.106 | 0.003 |
| clathrin heavy chain | *chcA* | 2.051 | 0.006 |
| mesoderm development candidate 1 | *mesdc1* | 1.874 | 0.002 |
| nucleolar protein 56 | *nop56* | 1.830 | 0.009 |
| interleukin enhancer-binding factor 3 | *ilf3* | 1.798 | 0.002 |
| nucleolar protein 58 | *nop58* | 1.788 | 0.006 |
| chemokine CXC-like protein | *cxc* | 1.784 | 0.006 |
| tyrosine-protein kinase Blk | *blk* | 1.756 | 0.002 |
| pre-mRNA processing factor 4 homolog | *prpf4* | 1.716 | 0.009 |
| protein-tyrosine sulfotransferase 1 | *tpst1* | 1.656 | 0.002 |
| hypoxia-inducible factor | *hif* | 1.640 | 0.007 |
| band 4.1-like protein 1 | *epb41l1* | 1.614 | 0.007 |
| nucleolar complex associated 2 homolog | *noc2l* | 1.597 | 0.008 |
| apoptosis-stimulating protein of p53 protein 2 | *tp53bp2* | 1.594 | 0.008 |
| transcription initiation factor TFIID subunit 3 | *taf3* | 1.519 | 0.008 |
| formin-like protein 1 | *fmnl1* | 1.518 | 0.008 |
| high affinity copper uptake protein 1 | *slc31a1* | -1.512 | 0.007 |
| transmembrane protein 150A | *tmem150a* | -1.555 | 0.008 |
| unknown protein |  | -1.588 | 0.008 |
| isoavaleryl-CoA dehydrogenase, mitochondrial | *ivd* | -1.589 | 0.007 |
| oxysterol-binding protein 1 | *osbp* | -1.627 | 0.007 |
| unknown protein |  | -1.806 | 0.008 |
| iron-sulfur cluster assembly enzyme ISCU, mit. | *iscu* | -1.816 | 0.008 |
| golgi pH regulator B | *gpr89b* | -1.829 | 0.008 |
| 2-oxoisovalerate dehydrogenase subunit alpha, mit. | *bckdha* | -1.873 | 0.008 |
| adrenomedullin-1 | *adm1* | -1.891 | 0.008 |
| Somatotropin | *gh* | -1.965 | 0.007 |
| CD151 antigen | *cd151* | -1.982 | 0.004 |
| calcipressin-1 | *rcan1* | -1.986 | 0.006 |
| glutathione S-transferase kappa 1 | *gstk1* | -2.002 | 0.007 |
| cytochrome b-c1 complex subunit 2, mitochondrial | *uqcrc2* | -2.042 | 0.008 |
| electron transfer flavoprotein subunit alpha, mit. | *etfa* | -2.076 | 0.008 |
| 3-hydroxybutyrate dehydrogenase type 2 | *bdh2* | -2.097 | 0.008 |
| unknown protein |  | -2.148 | 0.008 |
| RNA, 28S Ribosomal 1 | *rna28s1* | -2.165 | 0.004 |
| tetraspanin 1 | *tspan1* | -5.899 | 0.007 |
