# Supplementary material for: Effects of Changes in Food Supply at the Time of Sex Differentiation on the Gonadal Transcriptome of Juvenile Fish. Implications for Natural and Farmed Populations
Source: PLoS One. 2014 Oct 23;9(10):e111304. doi: 10.1371/journal.pone.0111304 (PMC4207807; doi:10.1371/journal.pone.0111304)
Supplement: Table S5 — Affected KEGG pathways in the F vs. S group comparison. (DOCX) [file pone.0111304.s009.docx]

Supplementary Table 5. Affected KEGG pathways for F vs. S comparison

| Pathways | # Sequences | # Enzymes | up/down |
| --- | --- | --- | --- |
| Alpha-Linolenic acid metabolism | 1 | 1 | down |
| Butanoate metabolism | 1 | 1 | up |
| Drug metabolism-cytochrome P450 | 1 | 1 | up |
| Glutathione metabolism | 1 | 2 | up |
| Linoleic acid metabolism | 1 | 1 | down |
| Metabolism of xenobiotics by cytochrome P450 | 1 | 1 | up |
| Synthesis and degradation of ketone bodies | 1 | 1 | up |
| T cell receptor signaling pathway | 1 | 1 | down |
| Valine, leucine and isoleucine degradation | 1 | 1 | up |
