# Supplementary material for: Effects of Changes in Food Supply at the Time of Sex Differentiation on the Gonadal Transcriptome of Juvenile Fish. Implications for Natural and Farmed Populations
Source: PLoS One. 2014 Oct 23;9(10):e111304. doi: 10.1371/journal.pone.0111304 (PMC4207807; doi:10.1371/journal.pone.0111304)
Supplement: Table S6 — DE gene list for the FF vs. SS group comparison. (DOCX) [file pone.0111304.s010.docx]

Supplementary Table 6. A detailed list of the DE genes for the FF versus SS comparison

| Description | Gene symbol | Fold change | Adjusted *P*-value |
| --- | --- | --- | --- |
| T-complex protein 1 subunit zeta | *cct6a* | 2.335 | 0.001 |
| basic leucine zipper and W2 domain-containing protein 2 | *bzw2* | 2.291 | 0.000 |
| 60S ribosomal protein L9 | *rpl9* | 2.275 | 0.006 |
| myelin-associated neurite-outgrowth inhibitor | *fam168b* | 2.262 | 0.003 |
| cyclic AMP-dependent transcription factor ATF4 | *atf4* | 2.170 | 0.006 |
| 60S ribosomal protein L18 | *rpl18* | 2.165 | 0.000 |
| splicing factor 3B subunit 1 | *sf3b1* | 2.143 | 0.005 |
| ubiquitin-conjugating enzyme E2 A | *ube2a* | 2.138 | 0.005 |
| voltage-dependent anion-selective channel protein 2 | *vdac2* | 2.121 | 0.001 |
| type II inositol 1,4,5-trisphosphate 5-phosphatase | *inpp5b* | 2.115 | 0.010 |
| 60S ribosomal protein L13 | *rpl13* | 2.104 | 0.000 |
| ATP synthase F(0) complex subunit C1, mitochondrial | *atp5g1* | 2.006 | 0.003 |
| Glutathione S-transferase Mu 1 | *gstm1* | 1.995 | 0.002 |
| 60S ribosomal protein L10 | *rpl10* | 1.984 | 0.003 |
| 40S ribosomal protein S17 | *rps17* | 1.967 | 0.000 |
| 40S ribosomal protein S30 | *rps30* | 1.941 | 0.002 |
| chaperonin containing TCP1, subunit *6A* (Zeta 1) | *cct6a* | 1.924 | 0.001 |
| nucleolar protein 58 | *nop58* | 1.917 | 0.009 |
| cleavage and polyadenylation specificity factor subunit 1 | *cpsf1* | 1.906 | 0.005 |
| thioredoxin, mitochondrial | *txn2* | 1.876 | 0.006 |
| 60S ribosomal protein L30 | *rpl30* | 1.874 | 0.000 |
| reverse transcriptase-like protein | *rtl* | 1.867 | 0.000 |
| 40S ribosomal protein S18 | *rps18* | 1.809 | 0.000 |
| 40S ribosomal protein S30 | *rps30* | 1.797 | 0.007 |
| polycomb protein SCMH1 | *scmh1* | 1.760 | 0.010 |
| cold-inducible RNA-binding protein | *cirbp* | 1.714 | 0.004 |
| 60S ribosomal protein L24 | *rpl24* | 1.695 | 0.004 |
| proteasome activator complex subunit 1 | *psme1* | 1.685 | 0.004 |
| presequence protease, mitochondrial | *pitrm1* | 1.682 | 0.006 |
| 40S ribosomal protein S14 | *rps14* | 1.682 | 0.000 |
| 40S ribosomal protein S8 | *rps8* | 1.679 | 0.000 |
| thyroid receptor-interacting protein 1 | *trip6* | 1.646 | 0.009 |
| transcription termination factor, mitochondrial | *mterf* | 1.637 | 0.001 |
| 40S ribosomal protein S4 | *rps4* | 1.624 | 0.001 |
| CG13731 | *cg13731-PA* | 1.618 | 0.009 |
| lanC-like protein 1 | *lancl1* | 1.607 | 0.010 |
| 40S ribosomal protein S10 | *rps10* | 1.591 | 0.001 |
| plakophilin-2 | *ppkp2* | 1.590 | 0.006 |
| phosphatidylserine synthase 1 | *ptdss1* | 1.565 | 0.003 |
| grb2-associated-binding protein 1 | *gab1* | 1.559 | 0.007 |
| golgin subfamily A member 5 | *golga5* | 1.545 | 0.009 |
| 60S ribosomal protein L18a | *rpl18a* | 1.524 | 0.004 |
| VHSV-induced protein | *ftr53* | 1.509 | 0.006 |
| isoleucine-tRNA ligase | *iars2* | 1.505 | 0.006 |
| 40S ribosomal protein S15 | *rps15* | -13.276 | 0.000 |
| 1-acyl-sn-glycerol-3-phosphate acyltransferase beta | *agpat2* | -12.126 | 0.000 |
| 7,8-dihydro-8-oxoguanine triphosphatase | *nudt1* | -9.391 | 0.000 |
| excitatory amino acid transporter 3 | *slc1a1* | -6.884 | 0.000 |
| carboxypeptidase N catalytic chain | *cpn1* | -6.731 | 0.000 |
| ubiquitin carboxyl-terminal hydrolase 33 | *usp33* | -6.600 | 0.003 |
| 26S proteasome non-ATPase regulatory subunit 13 | *psmd13* | -6.101 | 0.000 |
| platelet glycoprotein IX | *gp9* | -5.319 | 0.000 |
| histone chaperone ASF1A - Protein HIRA | *asf1a-hira* | -4.966 | 0.001 |
| E3 ubiquitin-protein ligase Midline-1 | *mid1* | -4.922 | 0.000 |
| 26S proteasome non-ATPase regulatory subunit 8 | *psmd8* | -4.654 | 0.000 |
| cystatin-A | *csta* | -4.464 | 0.000 |
| gamma-aminobutyric acid receptor subunit rho-2 | *gabrr2* | -4.359 | 0.000 |
| aquaporin-8 | *aqp8* | -3.972 | 0.000 |
| leucine-rich repeat-containing protein 40 | *lrrc40* | -3.884 | 0.008 |
| 60S ribosomal protein L35A | *rpl35a* | -3.684 | 0.000 |
| tetratricopeptide repeat protein 39C | *ttc39c* | -3.064 | 0.010 |
| protease serine 1 | *prss1* | -2.929 | 0.000 |
| protein FAM49B | *fam49b* | -2.843 | 0.001 |
| eukaryotic peptide chain release factor GTP-binding subunit ERF3A | *gspt1* | -2.838 | 0.000 |
| 60S ribosomal protein L32 | *rpl32* | -2.795 | 0.003 |
| propionyl-CoA carboxylase alpha chain, mitochondrial | *pcca* | -2.787 | 0.003 |
| peptidyl-prolyl cis-trans isomerase FKBP14 | *fkbp14* | -2.786 | 0.004 |
| 60S ribosomal protein L10 | *rpl10* | -2.751 | 0.007 |
| plexin-C1 | *plxnc1* | -2.632 | 0.000 |
| glutamate receptor 3 | *gria3* | -2.527 | 0.000 |
| CTP synthase 1 | *ctps1* | -2.492 | 0.008 |
| unhealthy ribosome biogenesis protein 2 homolog | *urb2* | -2.428 | 0.000 |
| 26S protease regulatory subunit 4 | *psmc1* | -2.332 | 0.003 |
| V-type proton ATPase 116 kDa subunit a isoform 1 | *atp6v* | -2.304 | 0.000 |
| armadillo repeat-containing protein 1 | *armc1* | -2.245 | 0.001 |
| 40S ribosomal protein S14 | *rps14* | -2.236 | 0.005 |
| U6 snRNA-associated Sm-like protein LSm4 | *lsm4* | -2.176 | 0.001 |
| 60S ribosomal protein L8 | *rpl8* | -2.155 | 0.000 |
| coatomer subunit epsilon | *cope* | -2.122 | 0.002 |
| E3 ubiquitin-protein ligase TRIM39 | *trim39* | -2.081 | 0.007 |
| ephrin-A1 | *efna1* | -2.063 | 0.007 |
| protein EURL homolog | *eurl* | -2.033 | 0.000 |
| type II inositol 1,4,5-trisphosphate 5-phosphatase | *inpp5a* | -2.031 | 0.000 |
| upstream-binding protein 1 | *ubp1* | -2.028 | 0.001 |
| steroid hormone receptor ERR2 | *esrrb* | -1.956 | 0.001 |
| 60S ribosomal protein L36A-like protein | *rpl36al* | -1.901 | 0.000 |
| 1-phosphatidylinositol 3-phosphate 5-kinase | *pip5k3* | -1.767 | 0.004 |
| 60S ribosomal protein L13A | *rpl13a* | -1.730 | 0.002 |
| bestrophin-2 | *best2* | -1.717 | 0.000 |
| ubiquitin-like modifier-activating enzyme ATG7 | *atg7* | -1.699 | 0.003 |
| peptide BmKa1 | *ka1* | -1.684 | 0.005 |
| GPI-linked NAD(P)(+)-arginine ADP-ribosyltransferase 1 | *art1* | -1.633 | 0.006 |
| butyrophilin-like protein 1 | *btnl1* | -1.612 | 0.003 |
| 60S ribosomal protein L30 | *rpl30* | -1.487 | 0.004 |
| protein-arginine deiminase type-2 | *padi2* | -1.450 | 0.005 |
| cyclic AMP-dependent transcription factor ATF-3 | *atf3* | -1.431 | 0.003 |
| asparagine--tRNA ligase, cytoplasmic | *nars* | -1.412 | 0.010 |
| tRNA (cytosine(38)-C(5))-methyltransferase | *trdmt1* | -1.389 | 0.007 |
| reverse transcriptase | *reverse transcriptase* | 1.360 | 0.008 |
| short/branched chain specific acyl-CoA dehydrogenase, mitochondrial | *acadsb* | 1.363 | 0.004 |
| craniofacial development protein 1 | *cfdp1* | 1.366 | 0.010 |
| sister chromatid cohesion protein DCC1 | *dscc1* | 1.367 | 0.002 |
| signal recognition particle 54 kDa protein | *srp54* | 1.384 | 0.008 |
| SET and MYND domain-containing protein 5 | *smyd5* | 1.400 | 0.001 |
| Renin | *ren* | 1.456 | 0.001 |
| 60S ribosomal protein L19 | *rpl19* | 1.462 | 0.006 |
| serine/threonine-protein kinase B-raf | *braf* | 1.478 | 0.007 |
| 1-acyl-sn-glycerol-3-phosphate acyltransferase epsilon | *agpat5* | 1.480 | 0.003 |
| shaw type potassium channel Kv3.3 | *kcnc3* | 1.484 | 0.002 |
| transposable element Tc3 transposase | *tc3a* | 1.485 | 0.003 |
| Prolactin | *prl* | 1.491 | 0.006 |
| 60S ribosomal protein L23 | *rpl23* | 1.492 | 0.006 |
| syntaxin interacting protein 1 | *sip1* | 1.497 | 0.003 |
