# Supplementary material for: Effects of Changes in Food Supply at the Time of Sex Differentiation on the Gonadal Transcriptome of Juvenile Fish. Implications for Natural and Farmed Populations
Source: PLoS One. 2014 Oct 23;9(10):e111304. doi: 10.1371/journal.pone.0111304 (PMC4207807; doi:10.1371/journal.pone.0111304)
Supplement: Table S7 — Affected KEGG pathways in the FF vs. SS group comparison. (DOCX) [file pone.0111304.s011.docx]

Supplementary Table 7. Affected KEGG pathways for FF vs. SS comparison

| Pathways | # Sequences | # Enzymes | up/down |
| --- | --- | --- | --- |
| Cysteine and methionin metabolism | 1 | 1 | down |
| Drug metabolism-cytochrom P450 | 1 | 1 | up |
| Fatty acid biosynthesis | 1 | 1 | down |
| Glutathione metabolism | 1 | 1 | up |
| Glyoxylate and dicarboxylate metabolism | 1 | 1 | down |
| Inositol phosphate metabolism | 1 | 1 | down |
| Metabolism of xenobiotics by cytochrome P450 | 1 | 1 | up |
| Phosphatidylinositol signaling system | 1 | 1 | down |
| Propanoate metabolism | 1 | 1 | down |
| Purine metabolism | 1 | 1 | down |
| Pyrimidine metabolism | 1 | 1 | down |
| Valine, leucine and isoleucine degradation | 1 | 1 | down |
