# Supplementary material for: Effects of Changes in Food Supply at the Time of Sex Differentiation on the Gonadal Transcriptome of Juvenile Fish. Implications for Natural and Farmed Populations
Source: PLoS One. 2014 Oct 23;9(10):e111304. doi: 10.1371/journal.pone.0111304 (PMC4207807; doi:10.1371/journal.pone.0111304)
Supplement: Table S8 — Two-tails Fisher's exact test with Multiple Testing Corrections of FDR results for the FF vs. SS group comparison. (DOCX) [file pone.0111304.s012.docx]

Supplementary Table 8. Two-tails Fisher’s exact test with multiple testing corrections of FDR results for the FF versus SS comparison

| GO Term | Name | Type | FDR | single test *P*-value | # in test group | # in reference group | over/  under |
| --- | --- | --- | --- | --- | --- | --- | --- |
|  |  |  |  |  |  |  |  |
| [GO:0003735](FisherInfo:GO:0003735) | structural constituent of ribosome | MF | 6,40E-13 | 5,80E-17 | 22 | 154 | over |
| [GO:0006415](FisherInfo:GO:0006415) | translational termination | BP | 1,80E-09 | 1,30E-12 | 12 | 43 | over |
| [GO:0006614](FisherInfo:GO:0006614) | SRP-dependent cotranslational protein targeting to membrane | BP | 1,90E-08 | 2,20E-11 | 12 | 57 | over |
| [GO:0000184](FisherInfo:GO:0000184) | nuclear-transcribed mRNA catabolic process, nonsense-mediated decay | BP | 2,30E-08 | 3,20E-11 | 12 | 59 | over |
| [GO:0006414](FisherInfo:GO:0006414) | translational elongation | BP | 2,10E-07 | 5,00E-10 | 12 | 77 | over |
| [GO:0019083](FisherInfo:GO:0019083) | viral transcription | BP | 2,10E-07 | 5,10E-10 | 11 | 59 | over |
| [GO:0022627](FisherInfo:GO:0022627) | cytosolic small ribosomal subunit | CC | 1,70E-06 | 4,60E-09 | 8 | 26 | over |
| [GO:0022625](FisherInfo:GO:0022625) | cytosolic large ribosomal subunit | CC | 1,10E-05 | 3,30E-08 | 8 | 35 | over |
| [GO:0006413](FisherInfo:GO:0006413) | translational initiation | BP | 1,60E-04 | 6,00E-07 | 11 | 125 | over |
| [GO:0000028](FisherInfo:GO:0000028) | ribosomal small subunit assembly | BP | 5,80E-03 | 3,30E-05 | 3 | 3 | over |
| [GO:0030490](FisherInfo:GO:0030490) | maturation of SSU-rRNA | BP | 1,40E-02 | 9,10E-05 | 3 | 5 | over |
| [GO:0005234](FisherInfo:GO:0005234) | extracellular-glutamate-gated ion channel activity | MF | 2,20E-02 | 1,40E-04 | 2 | 0 | over |
| [GO:0022624](FisherInfo:GO:0022624) | proteasome accessory complex | CC | 2,80E-02 | 1,90E-04 | 3 | 7 | over |
| [GO:0003723](FisherInfo:GO:0003723) | RNA binding | MF | 4,80E-02 | 3,40E-04 | 16 | 494 | over |
