# Supplementary material for: Effects of Changes in Food Supply at the Time of Sex Differentiation on the Gonadal Transcriptome of Juvenile Fish. Implications for Natural and Farmed Populations
Source: PLoS One. 2014 Oct 23;9(10):e111304. doi: 10.1371/journal.pone.0111304 (PMC4207807; doi:10.1371/journal.pone.0111304)
Supplement: Table S9 — DE gene list for the SF vs. SS group comparison. (DOCX) [file pone.0111304.s013.docx]

Supplementary Table 9. DE genes from SF versus SS comparison

| Description | Gene symbol | Fold change | Adjusted *P*-value |
| --- | --- | --- | --- |
| ribosomal protein L9 | *rpl9* | 2.606 | 0.001 |
| ribosomal protein L10 | *rpl10* | 2.362 | 0.000 |
| cold-inducible RNA-binding protein | *cirbp* | 2.283 | 0.005 |
| glutathione S-transferase Mu 1 | *gstm1* | 2.188 | 0.000 |
| ribosomal protein L13 | *rpl13* | 2.075 | 0.000 |
| ATP synthase F(0) complex subunit B1, mitochondrial | *atp5f1* | 2.063 | 0.003 |
| ribosomal protein S3 | *rps3* | 2.031 | 0.001 |
| ribosomal protein L18 | *rpl18* | 1.975 | 0.001 |
| ribosomal protein L24 | *rpl24* | 1.904 | 0.001 |
| ribosomal protein S17 | *rps17* | 1.867 | 0.000 |
| Polyubiquitin-C | *ubc* | 1.842 | 0.004 |
| T-complex protein 1 subunit eta | *cct7* | 1.772 | 0.003 |
| ribosomal protein S18 | *rps18* | 1.756 | 0.001 |
| ribosomal protein S8 | *rps8* | 1.728 | 0.000 |
| ribosomal protein S30 | *rps30* | 1.692 | 0.010 |
| ribosomal protein S14 | *rps14* | 1.690 | 0.001 |
| 40S ribosomal proteinS16 | *rps16* | 1.675 | 0.000 |
| plakophilin-2 | *pkp2* | 1.668 | 0.003 |
| proteasome activator complex subunit 1 | *psme1* | 1.663 | 0.008 |
| LanC-like protein 1 | *lancl1* | 1.651 | 0.009 |
| ribosomal protein L30 | *rpl30* | 1.650 | 0.007 |
| ribosomal protein S4 | *rps4* | 1.598 | 0.001 |
| elongation factor 1-alpha 1 | *eef1a1* | 1.559 | 0.007 |
| ribosomal protein L19 | *rpl19* | 1.545 | 0.003 |
| ribosomal protein L13a | *rpl13a* | 1.528 | 0.000 |
| phosphatidylserine synthase 1 | *ptdss1* | 1.519 | 0.008 |
| Propionyl-CoA carboxylase alpha chain, mitochondrial | *pcca* | 14.035 | 0.000 |
| 1-acyl-sn-glycerol-3-phosphate acyltransferase epsilon | *agpat5* | 13.439 | 0.000 |
| histone chaperone ASF1A/Protein HIRA | *asf1a-hira* | -6.974 | 0.000 |
| Prolactin | *prl* | -6.604 | 0.000 |
| 1-phosphatidylinositol 3-phosphate 5-kinase | *pip5k3* | -6.406 | 0.000 |
| short/branched chain specific acyl-CoA dehydrogenase, mitochondrial | *acadsb* | -6.317 | 0.000 |
| peptide BmKa1 | *ka1* | -6.247 | 0.000 |
| potassium voltage-gated channel subfamily C member 3 | *kcnc3* | -5.400 | 0.000 |
| E3 ubiquitin-protein ligase Midline-1 | *mid1* | -5.066 | 0.001 |
| MKIAA*0133 protein* | *urb2* | -4.649 | 0.003 |
| GPI-linked NAD(P)(+)-arginine ADP-ribosyltransferase 1 | *art1* | -4.232 | 0.000 |
| peptidyl-prolyl cis-trans isomerase FKBP14 | *fkbp14* | -4.127 | 0.000 |
| butyrophilin-like protein 1 | *btnl1* | -3.557 | 0.000 |
| dual specificity protein phosphatase 7 | *dusp7* | -3.455 | 0.010 |
| Renin | *ren* | -2.829 | 0.002 |
| craniofacial development protein 1 | *cfdp1* | -2.805 | 0.004 |
| ionotropic glutamate recetor subunit*3 alpha* | *fglur3a* | -2.775 | 0.000 |
| leucine-rich repeat-containing protein 40 | *lrrc40* | -2.773 | 0.000 |
| 26S proteasome non-ATPase regulatory subunit 13 | *psmd13* | -2.535 | 0.002 |
| cyclic AMP-dependent transcription factor ATF-3 | *atf3* | -2.533 | 0.001 |
| carboxypeptidase N, polypeptide 1 | *cpn1* | -2.428 | 0.000 |
| plexin-C1 | *plxnc1* | -2.426 | 0.000 |
| tRNA (cytosine(38)-C(5))-methyltransferase | *trdmt1* | -2.374 | 0.000 |
| V-type proton ATPase catalytic subunit A | *atp6v* | -2.288 | 0.000 |
| tetratricopeptide repeat protein 39C | *ttc39c* | -2.249 | 0.001 |
| chromosome 21 open reading frame 7 | *c21orf7* | -2.111 | 0.000 |
| chromodomain-helicase-DNA-binding protein 1-like | *chd1l* | -2.105 | 0.008 |
| delta-1-pyrroline-5-carboxylate synthase | *aldh18a* | -2.087 | 0.003 |
| syntaxin-binding protein 1 | *stxbp1* | -2.084 | 0.000 |
| protein-argi*nine deiminasae type II-*like | *?* | -1.963 | 0.001 |
| serine protease HTRA1 | *htra1* | -1.913 | 0.001 |
| dTDP-D-glucose 4,6-dehydratase | *tgds* | -1.842 | 0.003 |
| Cystatin | *cst* | -1.750 | 0.000 |
| armadillo repeat-containing protein 1 | *armc1* | -1.639 | 0.008 |
| sister chromatid cohesion protein DCC1 | *dscc1* | -1.619 | 0.003 |
