# Supplementary material for: Effects of Changes in Food Supply at the Time of Sex Differentiation on the Gonadal Transcriptome of Juvenile Fish. Implications for Natural and Farmed Populations
Source: PLoS One. 2014 Oct 23;9(10):e111304. doi: 10.1371/journal.pone.0111304 (PMC4207807; doi:10.1371/journal.pone.0111304)
Supplement: Table S10 — Affected KEGG pathways in the SF vs. SS group comparison. (DOCX) [file pone.0111304.s014.docx]

Supplementary Table 10. KEGG pathways of SF versus SS comparison

| Pathways | # Sequences | # Enzymes | up/down |
| --- | --- | --- | --- |
| Arginine and proline metabolism | 1 | 2 | down |
| Biosynthesis of vancomycin group antibiotics | 1 | 1 | down |
| Cysteine and methionine metabolism | 1 | 1 | down |
| Drug metabolism-cytochrome P450 | 1 | 1 | up |
| Fatty acid biosynthesis | 1 | 1 | down |
| Glutathione metabolism | 1 | 1 | up |
| Glycerolipid metabolism | 1 | 1 | down |
| Glycerophospholipid metabolism | 1 | 1 | down |
| Glyoxylate and dicarboxylate metabolism | 1 | 1 | down |
| Metabolism of xenobiotics by cytochrome P450 | 1 | 1 | up |
| Polyketide sugar unit biosynthesis | 1 | 1 | down |
| Propanoate metabolism | 1 | 1 | down |
| Sphingolipid metabolism | 1 | 1 | down |
| Streptomycin biosynthesis | 1 | 1 | down |
| Valine, leucine and isoleucine degradation | 1 | 1 | down |
