# Supplementary material for: Effects of Changes in Food Supply at the Time of Sex Differentiation on the Gonadal Transcriptome of Juvenile Fish. Implications for Natural and Farmed Populations
Source: PLoS One. 2014 Oct 23;9(10):e111304. doi: 10.1371/journal.pone.0111304 (PMC4207807; doi:10.1371/journal.pone.0111304)
Supplement: Table S11 — Two-tails Fisher's exact test with Multiple Testing Corrections of FDR results for the SF vs. SS group comparison. (DOCX) [file pone.0111304.s015.docx]

Supplementary Table 11. Fisher’s Exact Test with Multiple Corrections for FDR for SF vs. SS comparison

| GO Term | Name | Type | FDR | single test *P*-value | # in test group | # in reference group | Over/ |
| --- | --- | --- | --- | --- | --- | --- | --- |
|  |  |  |  |  |  |  | Under |
| [GO:0003735](FisherInfo:GO:0003735) | structural constituent of ribosome | MF | 3,40E-08 | 6,20E-12 | 14 | 162 | over |
| [GO:0022627](FisherInfo:GO:0022627) | cytosolic small ribosomal subunit | CC | 3,40E-06 | 2,10E-09 | 7 | 27 | over |
| [GO:0006415](FisherInfo:GO:0006415) | translational termination | BP | 6,70E-05 | 7,30E-08 | 7 | 48 | over |
| [GO:0006414](FisherInfo:GO:0006414) | translational elongation | BP | 9,90E-05 | 1,30E-07 | 8 | 81 | over |
| [GO:0006614](FisherInfo:GO:0006614) | SRP-dependent cotranslational protein targeting to membrane | BP | 2,00E-04 | 3,60E-07 | 7 | 62 | over |
| [GO:0019083](FisherInfo:GO:0019083) | viral transcription | BP | 2,00E-04 | 4,00E-07 | 7 | 63 | over |
| [GO:0000184](FisherInfo:GO:0000184) | nuclear-transcribed mRNA catabolic process, nonsense-mediated decay | BP | 2,00E-04 | 4,40E-07 | 7 | 64 | over |
| [GO:0000028](FisherInfo:GO:0000028) | ribosomal small subunit assembly | BP | 2,20E-03 | 5,90E-06 | 3 | 3 | over |
| [GO:0030490](FisherInfo:GO:0030490) | maturation of SSU-rRNA | BP | 4,70E-03 | 1,60E-05 | 3 | 5 | over |
| [GO:0006413](FisherInfo:GO:0006413) | translational initiation | BP | 9,00E-03 | 3,40E-05 | 7 | 129 | over |
| [GO:0005234](FisherInfo:GO:0005234) | extracellular-glutamate-gated ion channel activity | MF | 1,10E-02 | 4,60E-05 | 2 | 0 | over |
| [GO:0004970](FisherInfo:GO:0004970) | ionotropic glutamate receptor activity | MF | 2,70E-02 | 1,40E-04 | 2 | 1 | over |
| [GO:0022625](FisherInfo:GO:0022625) | cytosolic large ribosomal subunit | CC | 3,80E-02 | 2,00E-04 | 4 | 39 | over |
