# Supplementary material for: Effects of Changes in Food Supply at the Time of Sex Differentiation on the Gonadal Transcriptome of Juvenile Fish. Implications for Natural and Farmed Populations
Source: PLoS One. 2014 Oct 23;9(10):e111304. doi: 10.1371/journal.pone.0111304 (PMC4207807; doi:10.1371/journal.pone.0111304)
Supplement: Table S12 — DE gene list for the FS vs FF group comparison. (DOCX) [file pone.0111304.s016.docx]

Supplementary Table 12. DE genes for comparison FS versus FF

| Description | Gene symbol | Fold change | Adjusted *P*-value |
| --- | --- | --- | --- |
| Fatty acid-binding protein, heart | *fabp3* | -15.341 | 0.007 |
| Lipoprotein lipase | *lpl* | -14.392 | 0.006 |
| Mullerian-inhibiting factor | *amh* | -12.314 | 0.007 |
| Integrin beta-4 | *itgb4* | -6.390 | 0.006 |
| Actin-related protein 2/3 complex subunit 1 | *arpc1* | -5.203 | 0.008 |
| 26S protease regulatory subunit 10B | *psmc6* | -4.905 | 0.007 |
| V-type proton ATPase subunit B1 | *vha-b1* | -4.861 | 0.007 |
| Keratin, type II cytoskeletal 1 | *krt1* | -4.822 | 0.006 |
| Collagen alpha-1(XVIII) chain | *col18a* | -4.333 | 0.008 |
| NADH-ubiquinone oxidoreductase chain 1 | *mt-nd1* | -4.319 | 0.006 |
| NADH-ubiquinone oxidoreductase chain 4 | *mt-nd4* | -4.187 | 0.006 |
| Cytochrome c oxidase subunit 1 | *mt-co1* | -4.009 | 0.007 |
| Lipopolysaccharide-induced tumor necrosis factor-alpha factor | *litaf* | -3.663 | 0.009 |
| Ornithine decarboxylase | *odc1* | -3.547 | 0.002 |
| T-lymphoma invasion and metastasis-inducing protein 1 | *tiam1* | -3.502 | 0.006 |
| Unknown | ? | -3.452 | 0.006 |
| S-phase kinase-associated protein 1 | *skp1* | -3.441 | 0.005 |
| Retinol-binding protein 1 | *rbp1* | -3.439 | 0.007 |
| Thymosin beta-4 | *tmsb4x* | -3.171 | 0.008 |
| Proteasome inhibitor PI31 subunit | *psmf1* | -3.162 | 0.006 |
| NADH-ubiquinone oxidoreductase chain 5 | *mt-nd5* | -3.161 | 0.008 |
| Core histone macro-H2A.1 | *h2afy* | -3.159 | 0.005 |
| DAZ-associated protein 2 | *dazap2* | -3.157 | 0.006 |
| Keratin, type II cytoskeletal 2 epidermal | *krt2* | -3.155 | 0.007 |
| Serine incorporator 2 | *serinc2* | -3.081 | 0.003 |
| Structural maintenance of chromosomes protein 3 | *smc3* | -3.018 | 0.005 |
| Beta-2-microglobulin | *b2m* | -3.007 | 0.007 |
| Cyclic AMP-dependent transcription factor ATF-4 | *atf4* | -3.006 | 0.005 |
| Clathrin heavy chain 1 | *cltc* | -2.995 | 0.008 |
| PRELI domain-containing protein 1, mitochondrial | *prelid1* | -2.958 | 0.006 |
| DNA-directed RNA polymerases I, II, and III subunit RPABC4 | *polr2k* | -2.899 | 0.006 |
| CUGBP Elav-like family member 1 | *celf1* | -2.889 | 0.004 |
| Cytochrome b reductase 1 | *cybrd1* | -2.874 | 0.004 |
| Fatty aldehyde dehydrogenase | *aldh3a2* | -2.870 | 0.009 |
| Prostaglandin E synthase | *ptges* | -2.869 | 0.006 |
| 40S ribosomal protein S17 | *rps17* | -2.807 | 0.006 |
| Poly [ADP-ribose] polymerase 14 | *parp14* | -2.770 | 0.007 |
| Gelsolin | *gsn* | -2.768 | 0.009 |
| Calpain-2 catalytic subunit | *capn2* | -2.730 | 0.006 |
| Transmembrane protein 206 | *tmem206* | -2.725 | 0.007 |
| Negative elongation factor E | *nelfe* | -2.723 | 0.005 |
| Unknown | ? | -2.718 | 0.007 |
| T-complex protein 1 subunit zeta | *cct6a* | -2.660 | 0.002 |
| cAMP-dependent protein kinase type I-alpha regulatory subunit | *prkar1a* | -2.631 | 0.006 |
| Large proline-rich protein BAG6 | *bag6* | -2.625 | 0.004 |
| Splicing factor 3B subunit 1 | *sf3b1* | -2.615 | 0.003 |
| Heat shock 70 kDa protein 4 | *hspa4* | -2.570 | 0.008 |
| Peflin | *pef1* | -2.570 | 0.006 |
| Sorting nexin-1 | *snx1* | -2.555 | 0.007 |
| Unknown | ? | -2.539 | 0.006 |
| Peripheral plasma membrane protein CASK | *cask* | -2.531 | 0.005 |
| Myelin-associated neurite-outgrowth inhibitor | *fam168b* | -2.496 | 0.003 |
| Unknown | ? | -2.481 | 0.006 |
| Iron-sulfur cluster assembly enzyme ISCU, mitochondrial | *iscu* | -2.471 | 0.007 |
| NADH-ubiquinone oxidoreductase chain 6 | *mt-nd6* | -2.467 | 0.008 |
| RB1-inducible coiled-coil protein 1 | *rb1cc1* | -2.463 | 0.007 |
| Interferon-induced helicase C domain-containing protein 1 | *ifih1* | -2.452 | 0.007 |
| NF-kappa-B inhibitor-like protein 1 | *nfkbil1* | -2.445 | 0.006 |
| Caprin-1 | *caprin1* | -2.432 | 0.009 |
| Programmed cell death protein 4 | *pdcd4* | -2.432 | 0.005 |
| Histone H4 | *hist1h4a* | -2.428 | 0.005 |
| Zinc finger protein 1 | *znf1* | -2.413 | 0.006 |
| Dual specificity tyrosine-phosphorylationregulated kinase 1A | *dyrk1a* | -2.403 | 0.008 |
| Calmodulin-sensitive adenylate cyclase | *cya* | -2.394 | 0.006 |
| DNA repair protein RAD50 | *rad50* | -2.384 | 0.005 |
| Histone H3.1 | *hist1h3a* | -2.378 | 0.007 |
| GTP-Binding Protein 4 | *gbp4* | -2.378 | 0.008 |
| Ubiquitin-conjugating enzyme E2 D3 | *ube2d3* | -2.356 | 0.007 |
| Protein tyrosine phosphatase type IVA 2 | *ptp4a2* | -2.355 | 0.005 |
| E3 ubiquitin-protein ligase RNF13 | *rnf13* | -2.321 | 0.006 |
| Prostaglandin reductase 1 | *ptgr1* | -2.316 | 0.006 |
| E3 ubiquitin-protein ligase UBR1 | *ubr1* | -2.294 | 0.009 |
| Clathrin light chain B | *cltb* | -2.235 | 0.006 |
| Unknown | unknown | -2.234 | 0.008 |
| LIM domain-containing protein ajuba | *ajuba* | -2.224 | 0.006 |
| Unknown | ? | -2.220 | 0.007 |
| Vesicle transport protein SEC20 | *bnip1* | -2.211 | 0.007 |
| Peroxiredoxin-2 | *prdx2* | -2.197 | 0.010 |
| Von Hippel-Lindau disease tumor suppressor | *vhl* | -2.193 | 0.010 |
| Basic leucine zipper and W2 domain-containing protein 2 | *bzw2* | -2.184 | 0.000 |
| Serine/threonine-protein phosphatase 1 regulatory subunit 10 | *ppp1r10* | -2.136 | 0.009 |
| Unknown | ? | -2.124 | 0.006 |
| Eukaryotic initiation factor 4A-II | *eif4a2* | -2.119 | 0.006 |
| UPF0600 protein C5orf51 homolog | *wu:fd42g01* | -2.092 | 0.006 |
| MOB kinase activator 3A | *mob3a* | -2.062 | 0.006 |
| Ketosamine-3-kinase | *fn3krp* | -2.049 | 0.008 |
| Programmed cell death protein 10 | *pdcd10* | -2.044 | 0.006 |
| Utrophin | *utrn* | -2.036 | 0.007 |
| Serine/arginine-rich splicing factor 11 | *srsf11* | -2.029 | 0.009 |
| Histone deacetylase complex subunit SAP18 | *sap18* | -2.016 | 0.006 |
| Proteasome subunit alpha type-6 | *psma6* | -2.015 | 0.008 |
| Leucine-rich repeat, immunoglobulin-like domain and transmembrane domain-containing protein 1 | *lrit1* | -2.003 | 0.006 |
| Mt-myomegalin | *myomegalin* | -2.002 | 0.009 |
| Putative uncharacterized protein | *b230110c* | -1.993 | 0.007 |
| Proteasome subunit beta type-7 | *psmb7* | -1.970 | 0.007 |
| Heterogeneous nuclear ribonucleoprotein R | *hnrnpr* | -1.969 | 0.010 |
| B-cell CLL/lymphoma 7 protein family member B | *bcl7b* | -1.966 | 0.010 |
| Y-box-binding protein 3 | *ybx3* | -1.961 | 0.006 |
| Vacuolar protein sorting-associated protein 4B | *vps4b* | -1.959 | 0.007 |
| Transcriptional regulator ATRX | *atrx* | -1.957 | 0.010 |
| Porimin | *porimin* | -1.951 | 0.006 |
| Dystrophin | *dmd* | -1.947 | 0.009 |
| 26S protease regulatory subunit 4 | *psmc1* | -1.945 | 0.007 |
| Serine/threonine-protein kinase mTOR | *mtor* | -1.944 | 0.006 |
| ATP synthase subunit alpha, mitochondrial | *atp5a* | -1.943 | 0.006 |
| SWI/SNF complex subunit SMARCC1 | *smarcc1* | -1.929 | 0.007 |
| Dual specificity protein kinase CLK1 | *clk1* | -1.929 | 0.006 |
| Transmembrane prolyl 4-hydroxylase | *p4htm* | -1.927 | 0.006 |
| Probable dimethyladenosine transferase | *dimt1* | -1.922 | 0.008 |
| High mobility group protein B1 | *hmgb1* | -1.914 | 0.005 |
| Transcriptional repressor p66-alpha | *gatad2a* | -1.912 | 0.007 |
| Coagulation factor X | *f10* | -1.911 | 0.007 |
| FBP32 | *fbp32* | -1.907 | 0.010 |
| Male-specific lethal 3 homolog | *msl3* | -1.898 | 0.009 |
| Peptidyl-prolyl cis-trans isomerase FKBP3 | *fkbp3* | -1.897 | 0.007 |
| Splicing factor, arginine/serine-rich 19 | *scaf1* | -1.895 | 0.002 |
| Protein yippee-like 5 | *ypel5* | -1.891 | 0.008 |
| ETS translocation variant 1 | *etv1* | -1.878 | 0.008 |
| 39S ribosomal protein L53, mitochondrial | *mrpl53* | -1.872 | 0.007 |
| Protein Red | *ik* | -1.863 | 0.007 |
| DCN1-like protein 1 | *dcun1d1* | -1.849 | 0.008 |
| Protein max | *max* | -1.831 | 0.006 |
| Tetraspanin-13 | *tspan13* | -1.830 | 0.010 |
| Ubiquitin-conjugating enzyme E2 variant 2 | *ube2v2* | -1.821 | 0.008 |
| Unknown | ? | -1.820 | 0.007 |
| E3 ubiquitin-protein ligase RNF31 | *rnf31* | -1.797 | 0.009 |
| Dual specificity mitogen-activated protein kinase kinase 6 | *map2k6* | -1.790 | 0.008 |
| Forkhead box protein J2 | *foxj2* | -1.781 | 0.007 |
| NEDD4-like E3 ubiquitin-protein ligase WWP1 | *wwp1* | -1.780 | 0.007 |
| Charged multivesicular body protein 3 | *chmp3* | -1.757 | 0.007 |
| Forkhead box protein K1 | *foxk1* | -1.751 | 0.006 |
| cAMP-dependent protein kinase type I-alpha regulatory subunit | *prkar1a* | -1.744 | 0.008 |
| Tax1-binding protein 1 | *tax1bp1* | -1.734 | 0.007 |
| Inositol monophosphatase 1 | *impa1* | -1.733 | 0.009 |
| Apoptosis-stimulating of p53 protein 1 | *ppp1r13b* | -1.733 | 0.009 |
| Zinc finger CCCH domain-containing protein 7B | *zc3h7b* | -1.726 | 0.009 |
| Serine/threonine-protein kinase ICK | *ick* | -1.716 | 0.007 |
| RNA polymerase-associated protein RTF1 homolog | *rtf1* | -1.682 | 0.008 |
| EF-hand calcium-binding domain-containing protein 6 | *efcab6* | -1.680 | 0.009 |
| GDP-Man:Man(3)GlcNAc(2)-PP-Dol alpha-1,2-mannosyltransferase | *alg11* | -1.679 | 0.006 |
| Apoptosis-resistant E3 ubiquitin protein ligase 1 | *arel1* | -1.676 | 0.007 |
| Unknown | ? | -1.669 | 0.010 |
| Transcription factor p65 | *rela* | -1.659 | 0.007 |
| 28S ribosomal protein S33, mitochondrial | *mrps33* | -1.655 | 0.006 |
| Serine/threonine-protein kinase tousled-like 2 | *tlk2* | -1.634 | 0.006 |
| Unknown | ? | -1.623 | 0.010 |
| WD repeat domain phosphoinositide-interacting protein 2 | *wipi2* | -1.623 | 0.006 |
| Unknown | ? | -1.621 | 0.009 |
| E3 ubiquitin-protein ligase HUWE1 | *huwe1* | -1.615 | 0.010 |
| Cell division cycle and apoptosis regulator protein 1 | *ccar1* | -1.577 | 0.007 |
| UBX domain-containing protein 4 | *ubxn4* | -1.559 | 0.008 |
| Ubiquitin thioesterase OTUB1 | *otub1* | -1.537 | 0.006 |
| Complement C3 | *c3* | 18.227 | 0.007 |
| Cystatin-S | *cst4* | 16.203 | 0.007 |
| Geranylgeranyl pyrophosphate synthase | *ggps1* | 11.577 | 0.006 |
| Cytochrome c oxidase copper chaperone | *cox17* | 11.010 | 0.006 |
| Histone H2AX | *?* | 9.738 | 0.005 |
| Tetraspanin-13 | *tspan13* | 9.442 | 0.007 |
| Sorting nexin-10 | *snx10* | 7.389 | 0.007 |
| Cell division cycle protein 20 homolog | *cdc20* | 7.227 | 0.010 |
| Unknown | *?* | 6.897 | 0.007 |
| Cell death activator CIDE-3 | *cidec* | 6.714 | 0.010 |
| Cytochrome P450 26A1 | *cyp26a1* | 6.336 | 0.009 |
| Histone H2B type 1-C/E/F/G/I | *hist1h2bc* | 6.082 | 0.006 |
| Periphilin-1 | *pphln1* | 5.272 | 0.009 |
| Uridine-cytidine kinase 2 | *uck2* | 5.177 | 0.007 |
| Ribonucleoside-diphosphate reductase subunit M2 | *rrm2* | 4.848 | 0.009 |
| Tetraspanin-13 | *tspan13* | 4.796 | 0.009 |
| Nuclear autoantigenic sperm protein | *nasp* | 4.793 | 0.010 |
| Zona pellucida sperm-binding protein 5 | *zpc5* | 4.683 | 0.007 |
| Cytochrome c oxidase subunit 5A | *cox5a* | 4.612 | 0.006 |
| ER membrane protein complex subunit 8 | *emc8* | 4.594 | 0.006 |
| Selenoprotein H | *selh* | 4.463 | 0.006 |
| Complex III assembly factor LYRM7 | *lyrm7* | 4.411 | 0.008 |
| DNA-directed RNA polymerases I, II, and III subunit RPABC3 | *polr2h* | 4.337 | 0.007 |
| Glutaredoxin-2, mitochondrial | *glrx2* | 4.189 | 0.007 |
| Elongation factor Ts, mitochondrial | *tsfm* | 4.110 | 0.007 |
| Protein FAM60A | *fam60a* | 4.008 | 0.006 |
| Acetyl-CoA acetyltransferase, mitochondrial | *acat1* | 3.978 | 0.006 |
| Transcription factor SOX-3 | *sox3* | 3.865 | 0.006 |
| Prefoldin subunit 1 | *pfdn1* | 3.742 | 0.006 |
| Hepcidin | *hamp* | 3.714 | 0.007 |
| THAP domain-containing protein 4 | *thap4* | 3.679 | 0.007 |
| Ceroid-lipofuscinosis neuronal protein 5 | *cln5* | 3.645 | 0.006 |
| Unknown | ? | 3.593 | 0.007 |
| Glutathione peroxidase 3 | *gpx3* | 3.561 | 0.010 |
| 39S ribosomal protein L17, mitochondrial | *mrpl17* | 3.512 | 0.005 |
| Inactive hydroxysteroid dehydrogenase-like protein 1 | *hsdl1* | 3.495 | 0.009 |
| Frataxin, mitochondrial | *fxn* | 3.422 | 0.007 |
| Wee1-like protein kinase | *wee1* | 3.377 | 0.003 |
| RNA-binding protein 7 | *rbm7* | 3.377 | 0.006 |
| Epididymal secretory protein E1 | *npc2* | 3.353 | 0.006 |
| DNA-directed RNA polymerase II subunit RPB11-a | *polr2j* | 3.316 | 0.008 |
| Si:busm1-211o13.10 | *si:busm1-211o13.10* | 3.302 | 0.006 |
| Peptidyl-prolyl cis-trans isomerase-like 1 | *ppil1* | 3.286 | 0.007 |
| Probable aminopeptidase NPEPL1 | *npepl1* | 3.239 | 0.006 |
| Lutropin-choriogonadotropic hormone receptor | *lhcgr* | 3.235 | 0.007 |
| Histone H2B type 1-C/E/F/G/I | *hist1h2bc* | 3.165 | 0.006 |
| NADH dehydrogenase [ubiquinone] 1 alpha subcomplex subunit 1 | *ndufa1* | 3.123 | 0.008 |
| Dual specificity protein phosphatase 1 | *dusp1* | 3.087 | 0.007 |
| Coenzyme Q-binding protein COQ10 homolog B, mitochondrial | *coq10b* | 3.087 | 0.008 |
| Eukaryotic translation initiation factor 4 gamma 1 | *eif4g1* | 3.055 | 0.009 |
| Probable cytosolic iron-sulfur protein assembly protein CIAO1 | *ciao1* | 3.050 | 0.005 |
| Probable tRNA pseudouridine synthase 1 | *trub1* | 3.046 | 0.007 |
| Histone H2B type 1-O | *hist1h2bo* | 3.045 | 0.008 |
| Ribonuclease P protein subunit p21 | *rpp21* | 3.027 | 0.006 |
| Unknown | *?* | 3.024 | 0.006 |
| U6 snRNA-associated Sm-like protein LSm1 | *lsm1* | 3.018 | 0.007 |
| Unknown | ? | 3.012 | 0.007 |
| Junctional adhesion molecule A | *f11r* | 3.005 | 0.008 |
| AKT-interacting protein | *aktip* | 2.981 | 0.007 |
| Unknown | *?* | 2.923 | 0.010 |
| Cyclin-dependent kinase 5 activator 1 | *cdk5r1* | 2.901 | 0.006 |
| MAD2L1-binding protein | *mad2l1bp* | 2.890 | 0.007 |
| Lactoylglutathione lyase | *glo1* | 2.884 | 0.008 |
| Mitochondrial translocator assembly and maintenance protein 41 homolog | *tamm41* | 2.884 | 0.006 |
| DTW domain-containing protein 2 | *dtwd2* | 2.869 | 0.009 |
| Inactive L-threonine 3-dehydrogenase, mitochondrial | *tdh* | 2.856 | 0.006 |
| GTP-binding nuclear protein Ran | *ran* | 2.830 | 0.006 |
| 28S ribosomal protein S24, mitochondrial | *mrps24* | 2.828 | 0.008 |
| 39S ribosomal protein L35, mitochondrial | *mrpl35* | 2.827 | 0.007 |
| Trans-L-3-hydroxyproline dehydratase | *l3hypdh* | 2.825 | 0.010 |
| Hnrp1 | *hnrp* | 2.794 | 0.006 |
| Succinate dehydrogenase [ubiquinone] flavoprotein subunit, mitochondrial | *sdh1* | 2.744 | 0.007 |
| Embryonic stem cell-specific 5-hydroxymethylcytosine-binding protein | *hmces* | 2.734 | 0.006 |
| BTB/POZ domain-containing protein KCTD14 | *kctd14* | 2.730 | 0.007 |
| Succinate dehydrogenase [ubiquinone] iron-sulfur subunit, mitochondrial | *sdhb* | 2.704 | 0.010 |
| DNA-directed RNA polymerase I subunit RPA12 | *znrd1* | 2.689 | 0.008 |
| Anaphase-promoting complex subunit 13 | *anapc13* | 2.683 | 0.007 |
| Fibroblast growth factor 8 | *fgf8* | 2.683 | 0.007 |
| Unknown | *?* | 2.668 | 0.007 |
| Putative ribosomal RNA methyltransferase 2 | *ftsj2* | 2.658 | 0.008 |
| MIT domain-containing protein 1 | *mitd1* | 2.630 | 0.005 |
| RNA 3'-terminal phosphate cyclase-like protein | *rcl1* | 2.605 | 0.007 |
| Nonstructural protein P125-2 | *p125* | 2.601 | 0.005 |
| Transcription factor BTF3 homolog 4 | *btf3l4* | 2.577 | 0.009 |
| N(4)-(beta-N-acetylglucosaminyl)-L-asparaginase | *aga* | 2.569 | 0.007 |
| Protein Dr1 | *dr1* | 2.555 | 0.009 |
| SNARE-associated protein Snapin | *snapin* | 2.549 | 0.005 |
| Glioma tumor suppressor candidate region gene 2 protein | *gltscr2* | 2.548 | 0.007 |
| Activator of basal transcription 1 | *abt1* | 2.526 | 0.006 |
| Methionine--tRNA ligase, mitochondrial | *mars2* | 2.514 | 0.008 |
| S-acyl fatty acid synthase thioesterase, medium chain | *olah* | 2.513 | 0.007 |
| ER membrane protein complex subunit 6 | *emc6* | 2.503 | 0.006 |
| Kinetochore-associated protein NSL1 homolog | *nsl1* | 2.500 | 0.006 |
| ALK tyrosine kinase receptor | *alk* | 2.491 | 0.006 |
| 39S ribosomal protein L33, mitochondrial | *mrpl33* | 2.487 | 0.007 |
| Transmembrane protein 53 | *tmem53* | 2.481 | 0.007 |
| Transmembrane protein 70, mitochondrial | *tmem70* | 2.476 | 0.006 |
| Mitogen-activated protein kinase 14 | *mapk14* | 2.454 | 0.006 |
| Vitamin K epoxide reductase complex subunit 1 | *vkorc1* | 2.453 | 0.006 |
| Mitochondrial import inner membrane translocase subunit Tim10 B | *timm10b* | 2.448 | 0.010 |
| 39S ribosomal protein L18, mitochondrial | *mrpl18* | 2.445 | 0.007 |
| Mitochondrial import inner membrane translocase subunit Tim9 | *timm9* | 2.444 | 0.009 |
| Histone-lysine N-methyltransferase SETDB1 | *setdb1* | 2.433 | 0.005 |
| MARVEL domain-containing protein 3 | *marveld3* | 2.423 | 0.007 |
| Myc proto-oncogene protein | *myc* | 2.417 | 0.007 |
| 28S ribosomal protein S12, mitochondrial | *mrps12* | 2.416 | 0.005 |
| AN1-type zinc finger protein 2B | *zfand2b* | 2.411 | 0.006 |
| AP-2 complex subunit alpha-1 | *ap2a* | 2.408 | 0.009 |
| Acylpyruvase FAHD1, mitochondrial | *fahd1* | 2.401 | 0.008 |
| Arginine--tRNA ligase, cytoplasmic | *rars* | 2.392 | 0.008 |
| 39S ribosomal protein L4, mitochondrial | *mrpl4* | 2.391 | 0.007 |
| Tetratricopeptide repeat protein 27 | *ttc27* | 2.390 | 0.008 |
| Sideroflexin-4 | *sfxn4* | 2.390 | 0.006 |
| LYR motif-containing protein 1 | *lyrm1* | 2.388 | 0.006 |
| 28S ribosomal protein S11, mitochondrial | *mrps11* | 2.386 | 0.007 |
| Catechol O-methyltransferase | *comt* | 2.376 | 0.007 |
| Acidic leucine-rich nuclear phosphoprotein 32 family member A | *anp32a* | 2.368 | 0.006 |
| Fc receptor-like protein 6 | *fcrl6* | 2.367 | 0.006 |
| Zgc:194819 protein | *faf2* | 2.360 | 0.009 |
| Transmembrane protein 199 | *tmem199* | 2.359 | 0.006 |
| Cytosol aminopeptidase | *lap3* | 2.337 | 0.006 |
| Membrane magnesium transporter 1 | *mmgt1* | 2.310 | 0.007 |
| Mediator of RNA polymerase II transcription subunit 27 | *med27* | 2.295 | 0.008 |
| Probable inactive tRNA-specific adenosine deaminase-like protein 3 | *adat3* | 2.293 | 0.007 |
| MARVEL domain-containing protein 1 | *marveld1* | 2.287 | 0.007 |
| 39S ribosomal protein L27, mitochondrial | *mrpl27* | 2.286 | 0.007 |
| Aurora kinase A-interacting protein | *aurkaip1* | 2.277 | 0.009 |
| Unknown | ? | 2.274 | 0.007 |
| PHD finger-like domain-containing protein 5A | *phf5a* | 2.273 | 0.010 |
| Sphingomyelin phosphodiesterase 2 | *smpd2* | 2.261 | 0.009 |
| Peroxisomal membrane protein 4 | *pxmp4* | 2.255 | 0.007 |
| HIG1 domain family member 2A | *higd2a* | 2.253 | 0.005 |
| General transcription factor 3C polypeptide 5 | *gtf3c5* | 2.252 | 0.006 |
| RNA pseudouridylate synthase domain-containing protein 1 | *rpusd1* | 2.244 | 0.003 |
| IP05929p | *cg14903* | 2.225 | 0.006 |
| COP9 signalosome complex subunit 3 | *cops3* | 2.216 | 0.006 |
| Glycine receptor subunit alpha-3 | *glra3* | 2.212 | 0.006 |
| 28S ribosomal protein S21, mitochondrial | *mrps21* | 2.205 | 0.007 |
| Protein THEM6 | *them6* | 2.204 | 0.006 |
| AP-1 complex subunit sigma-3 | *ap1s3* | 2.196 | 0.009 |
| HRAS-like suppressor 3 | *pla2g16* | 2.187 | 0.005 |
| NADH dehydrogenase [ubiquinone] 1 alpha subcomplex assembly factor 3 | *ndufaf3* | 2.183 | 0.005 |
| Transmembrane protein 134 | *tmem134* | 2.183 | 0.006 |
| Anaphase-promoting complex subunit CDC26 | *cdc26* | 2.181 | 0.007 |
| Ubiquitin carboxyl-terminal hydrolase isozyme L5 | *uchl5* | 2.173 | 0.007 |
| Putative deoxyribonuclease TATDN1 | *tatdn1* | 2.173 | 0.007 |
| N(4)-(beta-N-acetylglucosaminyl)-L-asparaginase | *aga* | 2.169 | 0.008 |
| Cysteine-rich with EGF-like domain protein 2 | *creld2* | 2.163 | 0.007 |
| Protein LZIC | *lzic* | 2.158 | 0.007 |
| Rab5 GDP/GTP exchange factor | *rabgef1* | 2.151 | 0.009 |
| Autophagy-related protein 16-1 | *atg16l1* | 2.141 | 0.006 |
| Mitochondrial ribosome-associated GTPase 1 | *mtg1* | 2.139 | 0.007 |
| Protein disulfide-isomerase-like protein of the testis | *pdilt* | 2.133 | 0.009 |
| Ribosome biogenesis protein WDR12 | *wdr12* | 2.129 | 0.007 |
| Acetyl-CoA acetyltransferase, cytosolic | *acat2* | 2.126 | 0.007 |
| SET and MYND domain-containing protein 4 | *smyd4* | 2.120 | 0.007 |
| Transmembrane protein 147 | *tmem147* | 2.115 | 0.008 |
| N-alpha-acetyltransferase 20 | *?* | 2.113 | 0.007 |
| Methyl-CpG-binding domain protein 3 | *mbd3* | 2.108 | 0.006 |
| Cell cycle control protein 50A | *tmem30a* | 2.108 | 0.007 |
| 11-cis retinol dehydrogenase | *rdh5* | 2.108 | 0.006 |
| Signal peptidase complex subunit 1 | *spcs1* | 2.106 | 0.008 |
| Unknown | ? | 2.105 | 0.009 |
| Zinc finger CCHC domain-containing protein 4 | *zcchc4* | 2.103 | 0.007 |
| 5-hydroxytryptamine receptor 5A | *htr5a* | 2.099 | 0.006 |
| tRNA pseudouridine synthase A, mitochondrial | *pus1* | 2.097 | 0.009 |
| Transcription factor p65 | *rela* | 2.093 | 0.009 |
| Lipase maturation factor 2 | *lmf2* | 2.086 | 0.009 |
| NudC domain-containing protein 1 | *nudcd1* | 2.083 | 0.007 |
| U3 small nucleolar RNA-interacting protein 2 | *rrp9* | 2.082 | 0.007 |
| 28S ribosomal protein S18a, mitochondrial | *mrps18a* | 2.081 | 0.009 |
| Mediator of RNA polymerase II transcription subunit 20 | *med20* | 2.080 | 0.010 |
| Methylosome protein 50 | *wdr77* | 2.080 | 0.010 |
| Mitochondrial import inner membrane translocase subunit Tim21 | *timm21* | 2.071 | 0.009 |
| V-type proton ATPase subunit F | *atp6v* | 2.071 | 0.006 |
| Serine protease HTRA2, mitochondrial | *htra2* | 2.068 | 0.007 |
| OX-2 membrane glycoprotein | *cd200* | 2.060 | 0.007 |
| Endoplasmic reticulum-Golgi intermediate compartment protein 2 | *ergic2* | 2.052 | 0.006 |
| ATP synthase subunit e, mitochondrial | *atp5i* | 2.045 | 0.010 |
| Pyrroline-5-carboxylate reductase 1, mitochondrial | *pycr1* | 2.033 | 0.006 |
| RWD domain-containing protein 2B | *rwdd2b* | 2.031 | 0.008 |
| UMP-CMP kinase 2, mitochondrial | *cmpk2* | 2.030 | 0.007 |
| 3-oxoacyl-[acyl-carrier-protein] reductase FabG | *fabg* | 2.028 | 0.008 |
| Phosphatidylinositol N-acetylglucosaminyltransferase subunit H | *pigh* | 2.027 | 0.006 |
| Tyrosyl-DNA phosphodiesterase 2 | *tdp2* | 2.026 | 0.008 |
| Zinc finger CCHC domain-containing protein 10 | *zcchc10* | 2.021 | 0.006 |
| 39S ribosomal protein L9, mitochondrial | *mrpl9* | 2.018 | 0.007 |
| Ubiquilin-4 | *ubqln4* | 2.011 | 0.007 |
| Mediator of RNA polymerase II transcription subunit 21 | *med21* | 2.008 | 0.009 |
| Vasopressin-neurophysin 2-copeptin | *avp* | 1.995 | 0.006 |
| Ribonucleases P/MRP protein subunit POP1 | *pop1* | 1.994 | 0.007 |
| N-acetylglucosamine-1-phosphotransferase subunit gamma | *gnptg* | 1.993 | 0.006 |
| 14 kDa phosphohistidine phosphatase | *phpt1* | 1.993 | 0.010 |
| ATP synthase F(0) complex subunit C1, mitochondrial | *atp5g1* | 1.988 | 0.010 |
| Inorganic pyrophosphatase | *ppa1* | 1.985 | 0.006 |
| Carboxypeptidase Z | *cpz* | 1.983 | 0.006 |
| UDP-N-acetylglucosamine transferase subunit ALG14 homolog | *alg14* | 1.982 | 0.006 |
| Bis(5'-nucleosyl)-tetraphosphatase | *nudt2* | 1.973 | 0.006 |
| Putative N-acetylglucosamine-6-phosphate deacetylase | *amdhd2* | 1.969 | 0.006 |
| Phosphatidylinositol transfer protein beta isoform | *pitpnb* | 1.968 | 0.009 |
| 26S proteasome non-ATPase regulatory subunit 10 | *psmd10* | 1.965 | 0.006 |
| Glucosylceramidase | *gba* | 1.960 | 0.010 |
| Palmitoyl-protein thioesterase 1 | *ppt1* | 1.957 | 0.007 |
| Low-density lipoprotein receptor | *ldlr* | 1.955 | 0.008 |
| Ribonucleases P/MRP protein subunit POP1 | *pop1* | 1.937 | 0.010 |
| Dehydrogenase/reductase SDR family member 7B | *dhrs7b* | 1.936 | 0.006 |
| Rab5 GDP/GTP exchange factor | *rabgef1* | 1.935 | 0.006 |
| ER membrane protein complex subunit 10 | *emc10* | 1.933 | 0.006 |
| Sodium bicarbonate cotransporter 3 | *slc4a7* | 1.929 | 0.005 |
| Peroxisomal membrane protein 11A | *pex11a* | 1.926 | 0.007 |
| Receptor-type tyrosine-protein phosphatase zeta | *ptprz1* | 1.915 | 0.009 |
| Acylamino-acid-releasing enzyme | *apeh* | 1.900 | 0.010 |
| Inosine triphosphate pyrophosphatase | *itpa* | 1.894 | 0.007 |
| Unknown | ? | 1.891 | 0.008 |
| Elongation factor 1-delta | *eef1d* | 1.890 | 0.007 |
| Long-chain-fatty-acid--CoA ligase 6 | *acsl6* | 1.884 | 0.008 |
| Glucosylceramidase | *gba* | 1.883 | 0.006 |
| Nitrogen permease regulator 3-like protein | *nprl3* | 1.880 | 0.006 |
| Phosphatidylinositol-glycan biosynthesis class F protein | *pigf* | 1.869 | 0.006 |
| Solute carrier family 35 member F2 | *slc35f2* | 1.867 | 0.007 |
| Mitochondrial inner membrane protein OXA1L | *oxa1l* | 1.864 | 0.009 |
| G patch domain and KOW motifs-containing protein | *gpkow* | 1.854 | 0.009 |
| Calponin-2 | *cnn2* | 1.851 | 0.009 |
| Nucleolar protein 16 | *nop16* | 1.850 | 0.007 |
| Protein KTI12 homolog | *kti12* | 1.847 | 0.009 |
| histone deacetylase 11 | *hdac11* | 1.839 | 0.007 |
| WD repeat-containing protein 41 | *wdr41* | 1.832 | 0.006 |
| 39S ribosomal protein L52, mitochondrial | *mrpl52* | 1.814 | 0.010 |
| Steroid receptor RNA activator 1 | *sra1* | 1.810 | 0.009 |
| Cirhin | *cirh1a* | 1.808 | 0.009 |
| Conserved oligomeric Golgi complex subunit 4 | *cog4* | 1.807 | 0.008 |
| Exosome complex component RRP4 | *exosc2* | 1.795 | 0.007 |
| Arylamine N-acetyltransferase 1 | *nat1* | 1.794 | 0.008 |
| V-type proton ATPase subunit G 1 | *atp6v* | 1.794 | 0.007 |
| Pseudouridine-5'-monophosphatase | *hdhd1* | 1.792 | 0.007 |
| MKI67 FHA domain-interacting nucleolar phosphoprotein | *nifk* | 1.790 | 0.006 |
| Unknown | ? | 1.790 | 0.010 |
| Unknown | *?* | 1.784 | 0.007 |
| CD82 antigen | *cd82* | 1.780 | 0.006 |
| Mitochondrial intermediate peptidase | *mipep* | 1.780 | 0.008 |
| THO complex subunit 7 homolog | *thoc7* | 1.775 | 0.008 |
| Beta-catenin-interacting protein 1 | *ctnnbip1* | 1.774 | 0.008 |
| Partitioning defective 6 homolog gamma | *pard6g* | 1.772 | 0.008 |
| E3 ubiquitin-protein ligase MYLIP | *mylip* | 1.771 | 0.009 |
| Solute carrier family 25 member 40 | *slc25a40* | 1.771 | 0.008 |
| Signal recognition particle subunit SRP72 | *srp72* | 1.769 | 0.008 |
| Histidine triad nucleotide-binding protein 3 | *hint3* | 1.768 | 0.008 |
| Unknown | ? | 1.764 | 0.008 |
| Tail-anchored protein insertion receptor WRB | *wrb* | 1.761 | 0.009 |
| U3 small nucleolar ribonucleoprotein protein IMP3 | *imp3* | 1.759 | 0.009 |
| E3 ubiquitin-protein ligase TRIM39 | *trim39* | 1.743 | 0.005 |
| SET and MYND domain-containing protein 5 | *smyd5* | 1.734 | 0.009 |
| DNA-directed RNA polymerases I and III subunit RPAC1 | *polr1c* | 1.734 | 0.009 |
| UPF0511 protein C2orf56 homolog | ? | 1.733 | 0.005 |
| Protein FAM193B | *fam193b* | 1.731 | 0.006 |
| Protein TBRG4 | *tbrg4* | 1.727 | 0.010 |
| Protein FAM46A | *fam46a* | 1.724 | 0.007 |
| Glycerol-3-phosphate acyltransferase 3 | *agpat9* | 1.721 | 0.006 |
| C-X-C motif chemokine 13 | *cxcl13* | 1.715 | 0.007 |
| Protein GTLF3B | *gtlf3b* | 1.712 | 0.009 |
| Tubulin alpha-1B chain | *tuba1b* | 1.710 | 0.007 |
| ATPase family AAA domain-containing protein 1 | *atad1* | 1.709 | 0.006 |
| Inositol monophosphatase 1 | *impa1* | 1.707 | 0.007 |
| Probable RNA-binding protein EIF1AD | *eif1ad* | 1.699 | 0.009 |
| COMM domain-containing protein 3 | *commd3* | 1.699 | 0.007 |
| UPF0402 protein | *egm_09475* | 1.692 | 0.007 |
| Trimethylguanosine synthase | *tgs1* | 1.691 | 0.008 |
| Syntaxin-5 | *stx5* | 1.691 | 0.007 |
| E3 ubiquitin-protein ligase RNF126 | *rnf126* | 1.687 | 0.009 |
| Glutathione synthetase | *gss* | 1.685 | 0.008 |
| Alpha/beta hydrolase domain-containing protein 14A | *abhd14a* | 1.683 | 0.009 |
| Ankyrin repeat domain-containing protein 16 | *ankrd16* | 1.678 | 0.010 |
| Acidic fibroblast growth factor intracellular-binding protein | *fibp* | 1.676 | 0.007 |
| C1GALT1-specific chaperone 1 | *c1galt1c1* | 1.669 | 0.008 |
| Spermatogenesis-associated protein 31A1 | *spata31a1* | 1.669 | 0.006 |
| Interferon-related developmental regulator 1 | *ifrd1* | 1.667 | 0.010 |
| E3 ubiquitin-protein ligase RAD18 | ? | 1.666 | 0.009 |
| Unknown | ? | 1.666 | 0.009 |
| Unknown | ? | 1.665 | 0.009 |
| FBP32 | *fbp32* | 1.661 | 0.008 |
| Unknown | ? | 1.659 | 0.007 |
| Molybdopterin synthase sulfur carrier subunit | *mocs2* | 1.656 | 0.010 |
| Osteopetrosis-associated transmembrane protein 1 | *ostm1* | 1.644 | 0.009 |
| Transcription factor Sp9 | *sp9* | 1.639 | 0.007 |
| 14 kDa phosphohistidine phosphatase | *phpt1* | 1.634 | 0.004 |
| snRNA-activating protein complex subunit 1 | *snapc1* | 1.630 | 0.008 |
| E3 ubiquitin-protein ligase RNF146 | *rnf146* | 1.628 | 0.006 |
| SH2 domain-containing protein 5 | *sh2d5* | 1.622 | 0.006 |
| Mitochondrial thiamine pyrophosphate carrier | *slc25a19* | 1.615 | 0.007 |
| Serine incorporator 3 | *serinc3* | 1.611 | 0.008 |
| Serine/threonine-protein phosphatase 2A [≈ High power LED current, peak 2.7 A] 55 kDa regulatory subunit B gamma isoform | *ppp2r2c* | 1.608 | 0.009 |
| Cell division control protein 42 homolog | *cdc42* | 1.606 | 0.007 |
| Prefoldin subunit 5 | *pfdn5* | 1.604 | 0.007 |
| Lariat debranching enzyme | *dbr1* | 1.602 | 0.007 |
| Dipeptidyl peptidase 4 | *dpp4* | 1.600 | 0.007 |
| PRELI domain-containing protein 1, mitochondrial | *prelid1* | 1.597 | 0.006 |
| Thioredoxin-related transmembrane protein 2 | *tmx2* | 1.593 | 0.009 |
| RISC-loading complex subunit TARBP2 | *tarbp2* | 1.590 | 0.007 |
| Dolichol-phosphate mannosyltransferase | *dpm1* | 1.568 | 0.009 |
| Unknown | *?* | 1.560 | 0.006 |
| 40S ribosomal protein SA | *rpsa* | 1.557 | 0.007 |
| Transcription termination factor, mitochondrial | *mterf* | 1.554 | 0.007 |
| MAPK-interacting and spindle-stabilizing protein-like | *mapk1ip1l* | 1.549 | 0.006 |
| Mitochondrial dynamics protein MID51 | *mief1* | 1.545 | 0.009 |
| Ecto-NOX disulfide-thiol exchanger 1 | *enox1* | 1.541 | 0.007 |
| Integral membrane protein 2C | *itm2c* | 1.541 | 0.010 |
| Cell cycle control protein 50A | *tmem30a* | 1.528 | 0.007 |
| Antizyme inhibitor 1 | *azin1* | 1.526 | 0.010 |
| Transcription factor IIIA | *gtf3a* | 1.523 | 0.008 |
| Grainyhead-like protein 2 homolog | *grhl2* | 1.521 | 0.010 |
| Magnesium transporter protein 1 | *magt1* | 1.514 | 0.008 |
| GPN-loop GTPase 3 | *gpn3* | 1.512 | 0.009 |
| Probable N-acetyltransferase 8B | *nat8b* | 1.504 | 0.006 |
| Phosphatidylinositol N-acetylglucosaminyltransferase subunit C | *pigc* | 1.503 | 0.009 |
| Mannose-1-phosphate guanyltransferase alpha | *gmppa* | 1.502 | 0.006 |
