# Supplementary material for: Effects of Changes in Food Supply at the Time of Sex Differentiation on the Gonadal Transcriptome of Juvenile Fish. Implications for Natural and Farmed Populations
Source: PLoS One. 2014 Oct 23;9(10):e111304. doi: 10.1371/journal.pone.0111304 (PMC4207807; doi:10.1371/journal.pone.0111304)
Supplement: Table S13 — Affected KEGG pathways in the FS vs. FF group comparison. (DOCX) [file pone.0111304.s017.docx]

Supplementary Table 13. KEGG pathways from FS versus FFcomparison

| Pathways | # Sequences | # Enzymes | up/ down |
| --- | --- | --- | --- |
| Amino sugar and nucleotide sugar metabolism | 1 | 1 | up |
| Aminoacyl-tRNA biosynthesis | 2 | 2 | up |
| Arachidonic acid metabolism | 2 | 2 | down |
| Arginine and proline metabolism | 1 | 1 | down |
| Arginine and proline metabolism | 2 | 2 | up |
| Benzoate degradation | 1 | 1 | up |
| Beta-Alanine metabolism | 1 | 1 | down |
| Betalain biosynthesis | 1 | 1 | up |
| Biosynthesis of unsaturated fatty acids | 1 | 1 | up |
| Biotin metabolism | 1 | 1 | up |
| Butanoate metabolism | 1 | 1 | up |
| Chloroalkane and chloroalkene degradation | 1 | 1 | down |
| Citrate cycle (TCA cycle) | 2 | 1 | up |
| Drug metabolism-cytochrome P450 | 2 | 2 | down |
| Drug-metabolism-other enzymes | 1 | 1 | up |
| Ether lipid metabolism | 1 | 1 | up |
| Fatty acid biosynthesis | 2 | 2 | up |
| Fatty acid metabolism | 1 | 1 | up |
| Fructose and mannose metabolism | 1 | 1 | up |
| Glutathione metabolism | 3 | 3 | up |
| Glycerolipid metabolism | 1 | 1 | up |
| Glycerophospholipid metabolism | 2 | 2 | up |
| Glycine, serine and threonine metabolism | 2 | 2 | down |
| Glycolysis/Gluconeogenesis | 2 | 2 | down |
| Glycosylphophatidylinositol (GPI)-anchor | 2 | 2 | up |
| Glycerolipid metabolism | 1 | 1 | down |
| Glyoxylate and dicarboxylate metabolism | 1 | 1 | up |
| Histidine metabolism | 1 | 1 | down |
| Lysine degradation | 2 | 2 | up |
| Metabolism of xenobiotics by cytochrome P450 | 2 | 2 | down |
| mTOR signaling pathway | 1 | 1 | up |
| Naphthalene degradation | 1 | 1 | down |
| N-Glycan biosynthesis | 1 | 1 | up |
| One carbon pool by folate | 1 | 1 | down |
| Other glycan degradation | 3 | 2 | up |
| Other types of O-glycan biosynthesis | 1 | 1 | up |
| Phenylalanine metabolism | 2 | 2 | down |
| Phenylpropanoid biosynthesis | 1 | 1 | down |
| Phosphonate and phosphinate metabolism | 1 | 1 | up |
| Porphyrin and chlorophyll metabolism | 1 | 1 | up |
| Propanoate metabolism | 1 | 1 | up |
| Purine metabolism | 11 | 7 | up |
| Pyrimidine metabolism | 11 | 6 | up |
| Pyruvate metabolism | 2 | 2 | up |
| Selenocompound metabolism | 1 | 1 | up |
| Sphingolipid metabolism | 3 | 2 | up |
| Steroid biosynthesis | 1 | 1 | up |
| Steroid hormone biosynthesis | 1 | 1 | up |
| Synthesis and degradation of ketone bodies | 1 | 1 | up |
| T cell receptor signaling pathway | 2 | 2 | up |
| Terpenoid backbone biosynthesis | 1 | 1 | up |
| Thiamine metabolism | 1 | 1 | up |
| Tryptophan metabolism | 1 | 1 | up |
| Ubiquinone and other terpenoid-quinone biosynthesis | 1 | 1 | up |
| Valine, leucine and isoleucine degradation | 1 | 1 | up |
