# Supplementary material for: Effects of Changes in Food Supply at the Time of Sex Differentiation on the Gonadal Transcriptome of Juvenile Fish. Implications for Natural and Farmed Populations
Source: PLoS One. 2014 Oct 23;9(10):e111304. doi: 10.1371/journal.pone.0111304 (PMC4207807; doi:10.1371/journal.pone.0111304)
Supplement: Table S14 — Two-tails Fisher's exact test with Multiple Testing Corrections for FDR results for the FS vs. FF group comparison. (DOCX) [file pone.0111304.s018.docx]

Supplementary Table 14. Fisher’s Exact Test with Multiple Corrections for FDR for FS vs. FF comparison

| GO Term | Name | Type | FDR | single test p-Value | # in test group | # in reference group | Over/ |
| --- | --- | --- | --- | --- | --- | --- | --- |
|  |  |  |  |  |  |  | Under |
| [GO:0005739](FisherInfo:GO:0005739) | Mitochondrion | CC | 1,60E-02 | 2,40E-05 | 79 | 852 | over |
| [GO:0000786](FisherInfo:GO:0000786) | Nucleosome | CC | 2,20E-02 | 4,30E-05 | 8 | 18 | over |
| [GO:0005730](FisherInfo:GO:0005730) | Nucleolus | CC | 2,80E-02 | 6,90E-05 | 47 | 442 | over |
| [GO:0004872](FisherInfo:GO:0004872) | receptor activity | MF | 2,80E-02 | 7,40E-05 | 3 | 300 | under |
| [GO:0010467](FisherInfo:GO:0010467) | gene expression | BP | 2,80E-02 | 1,10E-04 | 115 | 1435 | over |
| [GO:0010604](FisherInfo:GO:0010604) | positive regulation of macromolecule metabolic process | BP | 2,80E-02 | 1,50E-04 | 50 | 494 | over |
| [GO:0051724](FisherInfo:GO:0051724) | NAD transporter activity | MF | 2,80E-02 | 1,50E-04 | 3 | 0 | over |
| [GO:0005347](FisherInfo:GO:0005347) | ATP transmembrane transporter activity | MF | 2,80E-02 | 1,50E-04 | 3 | 0 | over |
| [GO:0051018](FisherInfo:GO:0051018) | protein kinase A binding | MF | 2,80E-02 | 1,50E-04 | 3 | 0 | over |
| [GO:0043132](FisherInfo:GO:0043132) | NAD transport | BP | 2,80E-02 | 1,50E-04 | 3 | 0 | over |
| [GO:0015867](FisherInfo:GO:0015867) | ATP transport | BP | 2,80E-02 | 1,50E-04 | 3 | 0 | over |
| [GO:0015866](FisherInfo:GO:0015866) | ADP transport | BP | 2,80E-02 | 1,50E-04 | 3 | 0 | over |
| [GO:0035350](FisherInfo:GO:0035350) | FAD transmembrane transport | BP | 2,80E-02 | 1,50E-04 | 3 | 0 | over |
| [GO:0035349](FisherInfo:GO:0035349) | coenzyme A transmembrane transport | BP | 2,80E-02 | 1,50E-04 | 3 | 0 | over |
| [GO:0014911](FisherInfo:GO:0014911) | positive regulation of smooth muscle cell migration | BP | 2,80E-02 | 1,50E-04 | 3 | 0 | over |
| [GO:0015230](FisherInfo:GO:0015230) | FAD transmembrane transporter activity | MF | 2,80E-02 | 1,50E-04 | 3 | 0 | over |
| [GO:0015228](FisherInfo:GO:0015228) | coenzyme A transmembrane transporter activity | MF | 2,80E-02 | 1,50E-04 | 3 | 0 | over |
| [GO:0015217](FisherInfo:GO:0015217) | ADP transmembrane transporter activity | MF | 2,80E-02 | 1,50E-04 | 3 | 0 | over |
| [GO:0080122](FisherInfo:GO:0080122) | AMP transmembrane transporter activity | MF | 2,80E-02 | 1,50E-04 | 3 | 0 | over |
| [GO:0080121](FisherInfo:GO:0080121) | AMP transport | BP | 2,80E-02 | 1,50E-04 | 3 | 0 | over |
| [GO:0031325](FisherInfo:GO:0031325) | positive regulation of cellular metabolic process | BP | 2,90E-02 | 1,60E-04 | 50 | 498 | over |
| [GO:0034645](FisherInfo:GO:0034645) | cellular macromolecule biosynthetic process | BP | 3,60E-02 | 2,10E-04 | 108 | 1348 | over |
| [GO:0006334](FisherInfo:GO:0006334) | nucleosome assembly | BP | 4,80E-02 | 3,10E-04 | 10 | 41 | over |
