# Supplementary material for: Effects of Changes in Food Supply at the Time of Sex Differentiation on the Gonadal Transcriptome of Juvenile Fish. Implications for Natural and Farmed Populations
Source: PLoS One. 2014 Oct 23;9(10):e111304. doi: 10.1371/journal.pone.0111304 (PMC4207807; doi:10.1371/journal.pone.0111304)
Supplement: Table S15 — DE gene list for the FS vs. SS group comparison. (DOCX) [file pone.0111304.s019.docx]

Supplementary Table 15. DE genes from the comparison FS vs. SS

| Description | Gene symbol | Fold change | Adjusted *P*-value |
| --- | --- | --- | --- |
| Carbonic anhydrase 1 | *ca1* | 36.701 | 0.004 |
| Complement receptor-like | *?* | 18.232 | 0.004 |
| Tetraspanin-13 | *tspan13* | 13.125 | 0.002 |
| Gamma-interferon-inducible lysosomal thiol reductase | *ifi30* | 12.332 | 0.004 |
| Cytochrome c oxidase copper chaperone | *cox17* | 12.064 | 0.002 |
| Mid1-interacting protein 1 | *mid1ip1* | 11.400 | 0.001 |
| Histone H2AX | *h2afx* | 10.117 | 0.001 |
| 50S ribosomal protein L4 | *rpld* | 9.307 | 0.004 |
| Geranylgeranyl pyrophosphate synthase | *ggps1* | 9.157 | 0.003 |
| Apo-Eif4aiii | *aurandraft_58937* | 8.632 | 0.002 |
| Cell death activator CIDE-3 | *cidec* | 8.194 | 0.003 |
| Sorting nexin-10 | *snx10* | 7.612 | 0.003 |
| Cytochrome P450 26A1 | *cyp26a1* | 7.406 | 0.003 |
| Tetraspanin-13 | *tspan13* | 6.542 | 0.002 |
| Metallothionein-2 | *mt2a* | 6.233 | 0.009 |
| Histone H2A | *h2a.zl1* | 6.168 | 0.001 |
| Cytoplasmic dynein 1 heavy chain 1 | *dync1h1* | 6.034 | 0.004 |
| Periphilin-1 | *pphln1* | 5.963 | 0.004 |
| Histone H2B 1/2/3/4/6 | *h2b-i* | 5.540 | 0.003 |
| Nuclear autoantigenic sperm protein | *nasp* | 5.534 | 0.004 |
| HAUS augmin-like complex subunit 6 | *haus6* | 5.472 | 0.002 |
| Multifunctional protein ADE2 | *paics* | 5.434 | 0.002 |
| Translation initiation factor eIF-2B subunit alpha | *eif2b1* | 5.305 | 0.001 |
| Erythropoietin | *epo* | 5.042 | 0.005 |
| Uridine-cytidine kinase 2 | *uck2* | 5.042 | 0.004 |
| Glutathione peroxidase 3 | *gpx3* | 4.996 | 0.002 |
| Cytochrome c oxidase subunit 5A | *cox5a* | 4.666 | 0.002 |
| Transcription factor SOX-3 | *sox3* | 4.507 | 0.001 |
| Coenzyme Q-binding protein COQ10 homolog B, mitochondrial | *coq10b* | 4.503 | 0.001 |
| 60S acidic ribosomal protein P0 | *rplp0* | 4.410 | 0.000 |
| Selenoprotein H | *selh* | 4.383 | 0.003 |
| DNA-directed RNA polymerases I, II, and III subunit RPABC3 | *polr2h* | 4.240 | 0.005 |
| Junctional adhesion molecule A | *f11r* | 4.237 | 0.001 |
| ATP-dependent RNA helicase DDX39 | *ddx39* | 4.157 | 0.006 |
| Glutaredoxin-2, mitochondrial | *glrx2* | 4.107 | 0.004 |
| ER membrane protein complex subunit 8 | *emc8* | 4.102 | 0.003 |
| Inactive hydroxysteroid dehydrogenase-like protein 1 | *hsdl1* | 4.097 | 0.001 |
| Fructose-bisphosphate aldolase B | *aldob* | 4.068 | 0.009 |
| Profilin-2 | *pfn2* | 4.053 | 0.010 |
| 28S ribosomal protein S24, mitochondrial | *mrps24* | 3.957 | 0.001 |
| Mitochondrial translocator assembly and maintenance protein 41 homolog | *tamm41* | 3.950 | 0.000 |
| Ceroid-lipofuscinosis neuronal protein 5 | *cln5* | 3.944 | 0.001 |
| Voltage-gated hydrogen channel 1 | *hvcn1* | 3.851 | 0.005 |
| 28S ribosomal protein S17, mitochondrial | *mrpl17* | 3.826 | 0.000 |
| Complex III assembly factor LYRM7 | *lyrm7* | 3.760 | 0.009 |
| Prefoldin subunit 1 | *pfdn1* | 3.724 | 0.002 |
| RNA, 5.8S Ribosomal 1 | *rn5-8s1* | 3.671 | 0.003 |
| Unknown | *?* | 3.670 | 0.002 |
| Betaine--homocysteine S-methyltransferase 1 | *bhmt* | 3.612 | 0.007 |
| Ribonuclease P protein subunit p21 | *rpp21* | 3.594 | 0.001 |
| Cyclin-dependent kinase 9 | *cdk9* | 3.578 | 0.002 |
| NADH dehydrogenase [ubiquinone] 1 alpha subcomplex subunit 1 | *ndufa1* | 3.570 | 0.001 |
| Putative surface protein | *mgas10750_spy1694* | 3.561 | 0.001 |
| Lactoylglutathione lyase | *glo1* | 3.529 | 0.001 |
| Protein FAM60A | *fam60a* | 3.521 | 0.002 |
| Nonstructural protein P125-2 | *p125* | 3.461 | 0.000 |
| Sodium/potassium-transporting ATPase subunit alpha-1 | *atp1a1* | 3.443 | 0.002 |
| RNA-binding protein 7 | *rbm7* | 3.422 | 0.002 |
| Dual specificity protein phosphatase 2 | *dusp2* | 3.355 | 0.002 |
| zona pellucida complex 5 | *zpc5* | 3.354 | 0.009 |
| THAP domain-containing protein 4 | *thap4* | 3.350 | 0.005 |
| Si:busm1-211o13.10 | *si:busm1-211o13.10* | 3.343 | 0.002 |
| DTW domain-containing protein 2 | *dtwd2* | 3.335 | 0.002 |
| Type II inositol 1,4,5-trisphosphate 5-phosphatase | *inpp5b* | 3.329 | 0.000 |
| Lamina-associated polypeptide 2, isoform alpha | *tmpo* | 3.303 | 0.000 |
| Elongation factor 1-delta | *eef1d* | 3.293 | 0.000 |
| Ribonuclease UK114 | *hrsp12* | 3.292 | 0.007 |
| MARVEL domain-containing protein 1 | *marveld1* | 3.285 | 0.000 |
| Transaldolase | *taldo1* | 3.281 | 0.002 |
| THAP domain-containing protein 1 | *thap1* | 3.269 | 0.000 |
| Lutropin-choriogonadotropic hormone receptor | *lhcgr* | 3.269 | 0.003 |
| ATP synthase subunit gamma, mitochondrial | *atp5c1* | 3.264 | 0.001 |
| Cob(I)yrinic acid a,c-diamide adenosyltransferase, mitochondrial | *mmab* | 3.260 | 0.003 |
| Epididymal secretory protein E1 | *npc2* | 3.245 | 0.002 |
| Endothelial lipase | *lipg* | 3.229 | 0.004 |
| Cyclin-dependent kinase 5 | *cdk5* | 3.218 | 0.001 |
| Putative deoxyribonuclease TATDN1 | *tatdn1* | 3.203 | 0.000 |
| Vitamin K epoxide reductase complex subunit 1 | *vkorc1* | 3.200 | 0.000 |
| Glioma tumor suppressor candidate region gene 2 protein | *gltscr2* | 3.178 | 0.001 |
| Vinculin | *deb-1* | 3.174 | 0.006 |
| Frataxin, mitochondrial | *fxn* | 3.172 | 0.003 |
| Lactotransferrin | *ltf* | 3.157 | 0.002 |
| Synaptonemal complex protein SC65 | *leprel4* | 3.130 | 0.003 |
| Sarcolemmal membrane-associated protein | *slmap* | 3.116 | 0.004 |
| Hnrp1 | *hnrp* | 3.097 | 0.001 |
| AN1-type zinc finger protein 2B | *zfand2b* | 3.094 | 0.000 |
| Isocitrate dehydrogenase [NADP], mitochondrial | *idh2* | 3.081 | 0.001 |
| Probable aminopeptidase NPEPL1 | *npepl1* | 3.041 | 0.002 |
| 39S ribosomal protein L22 | *rpl22* | 3.037 | 0.000 |
| histone cluster 1 | *hist1h2bo* | 3.027 | 0.005 |
| DNA primase small subunit | *prim1* | 3.022 | 0.003 |
| DNA-directed RNA polymerase II subunit RPB11-a | *polr2j* | 3.005 | 0.008 |
| Acidic leucine-rich nuclear phosphoprotein 32 family member A | *anp32a* | 2.993 | 0.001 |
| 39S ribosomal protein L27, mitochondrial | *mrpl27* | 2.987 | 0.001 |
| Cyclic AMP-dependent transcription factor ATF-4 | *atf4* | 2.981 | 0.000 |
| Serine/threonine-protein phosphatase PGAM5, mitochondrial | *pgam5* | 2.967 | 0.001 |
| Interferon-induced GTP-binding protein Mx1 | *mx1* | 2.958 | 0.006 |
| Acylamino-acid-releasing enzyme | *apeh* | 2.953 | 0.000 |
| Nucleoside diphosphate kinase A | *nme1* | 2.947 | 0.000 |
| Aurora kinase A-interacting protein | *aurkaip1* | 2.940 | 0.001 |
| Methionine--tRNA ligase, cytoplasmic | *mars* | 2.919 | 0.002 |
| ER membrane protein complex subunit 6 | *emc6* | 2.917 | 0.001 |
| Interactor protein for cytohesin exchange factors 1 | *ipcef1* | 2.914 | 0.002 |
| Kinesin-like protein KIF22 | *kif22* | 2.913 | 0.002 |
| Sorting nexin-24 | *snx24* | 2.896 | 0.010 |
| Myc proto-oncogene protein | *myc* | 2.888 | 0.001 |
| Transmembrane protein 208 | *tmem208* | 2.874 | 0.000 |
| MIT domain-containing protein 1 | *mitd1* | 2.871 | 0.000 |
| Aspartyl aminopeptidase | *dnpep* | 2.868 | 0.001 |
| ER membrane protein complex subunit 10 | *emc10* | 2.858 | 0.006 |
| BTB/POZ domain-containing protein KCTD14 | *kctd14* | 2.857 | 0.003 |
| MARVEL domain-containing protein 3 | *marveld3* | 2.855 | 0.001 |
| Signal recognition particle 9 kDa protein | *srp9* | 2.844 | 0.002 |
| Succinate dehydrogenase assembly factor 2, mitochondrial | *emi5* | 2.831 | 0.003 |
| 5-methylcytosine rRNA methyltransferase NSUN4 | *nsun4* | 2.827 | 0.001 |
| Wee1-like protein kinase | *wee1* | 2.825 | 0.001 |
| Catechol O-methyltransferase | *comt* | 2.816 | 0.001 |
| Transcription factor BTF3 homolog 4 | *btf3l4* | 2.786 | 0.007 |
| Transmembrane protein 70, mitochondrial | *tmem70* | 2.782 | 0.001 |
| N(4)-(beta-N-acetylglucosaminyl)-L-asparaginase | *aga* | 2.782 | 0.001 |
| Protein Tob1 | *tob1* | 2.779 | 0.005 |
| Oxysterol-binding protein-related protein 3 | *osbpl3* | 2.770 | 0.002 |
| Trans-L-3-hydroxyproline dehydratase | *l3hypdh* | 2.769 | 0.007 |
| Cytochrome P450 27C1 | *cyp27c* | 2.768 | 0.004 |
| THO complex subunit 4 | *alyref* | 2.763 | 0.004 |
| Activator of basal transcription 1 | *abt1* | 2.757 | 0.001 |
| Methylosome protein 50 | *wdr77* | 2.747 | 0.005 |
| Embryonic stem cell-specific 5-hydroxymethylcytosine-binding protein | *hmces* | 2.740 | 0.001 |
| Cell cycle control protein 50A | *tmem30a* | 2.740 | 0.000 |
| Aktip protein | *aktip* | 2.738 | 0.002 |
| RNA polymerase II subunit A C-terminal domain phosphatase SSU72 | *ssu72* | 2.738 | 0.002 |
| 28S ribosomal protein S18a, mitochondrial | *mrps18a* | 2.733 | 0.001 |
| 39S ribosomal protein L18, mitochondrial | *mrpl18* | 2.731 | 0.002 |
| Mitotic spindle assembly checkpoint protein MAD2B | *mad2l2* | 2.730 | 0.004 |
| Ras-related protein Rab-8A | *rab8a* | 2.728 | 0.004 |
| Chromobox protein homolog 5 | *cbx5* | 2.728 | 0.005 |
| Argininosuccinate synthase | *ass1* | 2.711 | 0.006 |
| S-acyl fatty acid synthase thioesterase, medium chain | *olah* | 2.706 | 0.002 |
| Histone-lysine N-methyltransferase SETDB1 | *setdb1* | 2.706 | 0.000 |
| Zinc finger CCHC domain-containing protein 4 | *zcchc4* | 2.704 | 0.002 |
| LYR motif-containing protein 1 | *lyrm1* | 2.700 | 0.001 |
| Mitochondrial import inner membrane translocase subunit Tim10 B | *timm10b* | 2.696 | 0.003 |
| Biogenesis of lysosome-related organelles complex 1 subunit 1 | *bloc1s1* | 2.694 | 0.001 |
| Tetratricopeptide repeat protein 27 | *ttc27* | 2.694 | 0.002 |
| Palmitoyl-protein thioesterase 1 | *ppt1* | 2.689 | 0.000 |
| SUMO-activating enzyme subunit 2 | *uba2* | 2.682 | 0.007 |
| Unknown | *?* | 2.673 | 0.001 |
| Mitogen-activated protein kinase 14 | *mapk14* | 2.659 | 0.001 |
| 28S ribosomal protein S2, mitochondrial | *mrps2* | 2.658 | 0.001 |
| Transmembrane protein 134 | *tmem134* | 2.651 | 0.000 |
| Peroxisomal membrane protein 4 | *pxmp4* | 2.651 | 0.001 |
| Fibroblast growth factor 8 | *fgf8* | 2.651 | 0.004 |
| Mitochondrial import inner membrane translocase subunit Tim21 | *timm21* | 2.649 | 0.001 |
| RNA 3'-terminal phosphate cyclase-like protein | *rcl1* | 2.648 | 0.002 |
| AP-1 complex subunit sigma-3 | *ap1s3* | 2.647 | 0.002 |
| Transcription initiation protein SPT3 homolog | *supt3h* | 2.640 | 0.002 |
| G patch domain and KOW motifs-containing protein | *gpkow* | 2.639 | 0.001 |
| Protein KTI12 homolog | *kti12* | 2.626 | 0.001 |
| Sideroflexin-4 | *sfxn4* | 2.624 | 0.001 |
| Succinate dehydrogenase [ubiquinone] iron-sulfur subunit, mitochondrial | *sdhb* | 2.614 | 0.008 |
| Transmembrane protein 147 | *tmem147* | 2.607 | 0.001 |
| Acyl-CoA dehydrogenase family member 11 | *acad11* | 2.607 | 0.001 |
| Autophagy-related protein 16-1 | *atg16l1* | 2.597 | 0.000 |
| Rab5 GDP/GTP exchange factor | *rabgef1* | 2.597 | 0.001 |
| Protein disulfide-isomerase-like protein of the testis | *pdilt* | 2.593 | 0.001 |
| Lipid phosphate phosphatase-related protein type 4 | *lppr4* | 2.583 | 0.008 |
| 39S ribosomal protein L35, mitochondrial | *mrpl35* | 2.570 | 0.008 |
| Very-long-chain (3R)-3-hydroxyacyl-[acyl-carrier protein] dehydratase 3 | *ptplad1* | 2.560 | 0.004 |
| U3 small nucleolar RNA-interacting protein 2 | *rrp9* | 2.559 | 0.002 |
| HMG domain-containing protein 4 | *hmgxb4* | 2.558 | 0.003 |
| Putative ribosomal RNA methyltransferase 2 | *ftsj2* | 2.549 | 0.002 |
| Fc receptor-like protein 6 | *fcrl6* | 2.545 | 0.001 |
| Putative N-acetylglucosamine-6-phosphate deacetylase | *amdhd2* | 2.544 | 0.000 |
| Coiled-coil domain-containing protein R3HCC1L | *r3hcc1l* | 2.530 | 0.002 |
| Homeobox protein VOX2 | *vox2* | 2.530 | 0.005 |
| General transcription factor 3C polypeptide 5 | *gtf3c5* | 2.525 | 0.001 |
| EH domain-binding protein 1 | *ehbp1* | 2.511 | 0.002 |
| Probable cytosolic iron-sulfur protein assembly protein CIAO1 | *ciao1* | 2.502 | 0.003 |
| 39S ribosomal protein L15, mitochondrial | *mrpl15* | 2.501 | 0.001 |
| Trafficking protein particle complex subunit 5 | *trappc5* | 2.497 | 0.001 |
| Serine protease HTRA2, mitochondrial | *htra2* | 2.495 | 0.001 |
| Cytosol aminopeptidase | *lap3* | 2.494 | 0.001 |
| Tyrosyl-DNA phosphodiesterase 2 | *tdp2* | 2.489 | 0.003 |
| 39S ribosomal protein L10 | *rpl10* | 2.484 | 0.000 |
| Zinc finger protein 518A | *znf518a* | 2.482 | 0.002 |
| Transcription factor p65 | *rela* | 2.481 | 0.002 |
| Cytochrome b-c1 complex subunit 1, mitochondrial | *uqcrc1* | 2.457 | 0.003 |
| Cellular tumor antigen p53 | *tp53* | 2.433 | 0.006 |
| Arginine--tRNA ligase, cytoplasmic | *rars* | 2.432 | 0.002 |
| Ubiquitin carboxyl-terminal hydrolase isozyme L5 | *uchl5* | 2.425 | 0.002 |
| Transmembrane protein 199 | *tmem199* | 2.415 | 0.002 |
| DnaJ-like subfamily B member 11 | *hsp40b11* | 2.404 | 0.003 |
| U6 snRNA-associated Sm-like protein LSm1 | *lsm1* | 2.401 | 0.010 |
| Nucleophosmin | *npm1* | 2.398 | 0.002 |
| 39S ribosomal protein L4, mitochondrial | *mrpl4* | 2.390 | 0.004 |
| 28S ribosomal protein S11, mitochondrial | *mrps11* | 2.378 | 0.004 |
| Sphingomyelin phosphodiesterase | *smpd1* | 2.367 | 0.003 |
| U3 small nucleolar ribonucleoprotein protein IMP3 | *imp3* | 2.361 | 0.002 |
| Acetyl-CoA acetyltransferase, cytosolic | *acat2* | 2.358 | 0.001 |
| 39S ribosomal protein L9, mitochondrial | *mrpl9* | 2.347 | 0.002 |
| Ancient ubiquitous protein 1 | *aup1* | 2.339 | 0.003 |
| 15 kDa selenoprotein | *42248* | 2.339 | 0.002 |
| Interleukin enhancer-binding factor 2 | *ilf2* | 2.329 | 0.009 |
| Probable tRNA pseudouridine synthase 1 | *trub1* | 2.327 | 0.008 |
| Retinol dehydrogenase 1 (All trans) | *rdh1* | 2.324 | 0.002 |
| snRNA-activating protein complex subunit 1 | *snapc1* | 2.319 | 0.000 |
| 40S ribosomal protein S12, mitochondrial | *tko* | 2.311 | 0.004 |
| 40S ribosomal protein SA | *rpsa* | 2.309 | 0.008 |
| Solute carrier family 35 member F2 | *slc35f2* | 2.309 | 0.000 |
| N-acetylglucosamine-1-phosphotransferase subunit gamma | *gnptg* | 2.307 | 0.001 |
| RING finger protein 113A | *rnf113a* | 2.306 | 0.002 |
| Mitochondrial import inner membrane translocase subunit Tim9 | *timm9* | 2.304 | 0.008 |
| Ubiquitin-like protein ATG12 | *atg12* | 2.302 | 0.002 |
| Ras-related protein Rab-32 | *rab32* | 2.301 | 0.001 |
| Long-chain-fatty-acid--CoA ligase 1 | *acsl1* | 2.298 | 0.004 |
| Double-stranded RNA-specific adenosine deaminase | *adar* | 2.296 | 0.002 |
| Factor in the germline alpha | *figla* | 2.292 | 0.008 |
| NADH dehydrogenase [ubiquinone] 1 alpha subcomplex subunit 9, mitochondrial | *ndufa9* | 2.283 | 0.006 |
| Glycine receptor subunit alpha-3 | *glra3* | 2.280 | 0.002 |
| HMG domain-containing protein 4 | *hmgxb4* | 2.276 | 0.001 |
| Protein disulfide-isomerase A4 | *pdia4* | 2.276 | 0.007 |
| Acyl-coenzyme A thioesterase 8 | *acot8* | 2.274 | 0.002 |
| Schwannomin-interacting protein 1 | *schip1* | 2.271 | 0.000 |
| Pyrroline-5-carboxylate reductase 1, mitochondrial | *pycr1* | 2.268 | 0.001 |
| Eukaryotic translation initiation factor 4E transporter | *eif4enif1* | 2.267 | 0.006 |
| Glucosidase 2 subunit beta | *prkcsh* | 2.266 | 0.004 |
| Proteasome subunit alpha type-3 | *psma3* | 2.249 | 0.004 |
| Long-chain-fatty-acid--CoA ligase 6 | *acsl6* | 2.247 | 0.001 |
| Protein THEM6 | *them6* | 2.247 | 0.002 |
| Fatty acyl-CoA reductase 2 | *far2* | 2.243 | 0.002 |
| Pseudouridine-5'-monophosphatase | *hdhd1* | 2.242 | 0.000 |
| AGAP012577-PA | *agap012577-pa* | 2.241 | 0.000 |
| COP9 signalosome complex subunit 3 | *cops3* | 2.240 | 0.002 |
| Protein MTO1 homolog, mitochondrial | *mto1* | 2.236 | 0.002 |
| Alanyl-tRNA editing protein Aarsd1 | *aarsd1* | 2.236 | 0.001 |
| ATP synthase subunit e, mitochondrial | *atp5i* | 2.235 | 0.003 |
| CDP-diacylglycerol--glycerol-3-phosphate 3-phosphatidyltransferase, mitochondrial | *pgs1* | 2.233 | 0.001 |
| RNA pseudouridylate synthase domain-containing protein 1 | *rpusd1* | 2.228 | 0.001 |
| Methyl-CpG-binding domain protein 3 | *mbd3* | 2.225 | 0.001 |
| Glucosylceramidase | *gba* | 2.225 | 0.001 |
| claudin 18 | *cldn18* | 2.225 | 0.002 |
| OTTMUSG00000010694 protein | *zfp600* | 2.222 | 0.004 |
| Membrane magnesium transporter 1 | *mmgt1* | 2.220 | 0.005 |
| Cyclin-dependent kinase 10 | *cdk10* | 2.218 | 0.007 |
| Cysteine-rich with EGF-like domain protein 2 | *creld2* | 2.216 | 0.003 |
| SET and MYND domain-containing protein 4 | *smyd4* | 2.215 | 0.002 |
| UDP-N-acetylglucosamine transferase subunit ALG14 homolog | *alg14* | 2.213 | 0.001 |
| Thioredoxin-2 | *trx2* | 2.198 | 0.000 |
| Ectonucleoside triphosphate diphosphohydrolase 8 | *entpd8* | 2.192 | 0.000 |
| DNA primase large subunit | *prim2* | 2.191 | 0.005 |
| Metaxin-2 | *mtx2* | 2.190 | 0.004 |
| FGFR1 oncogene partner 2 | *fgfr1op2* | 2.182 | 0.001 |
| Mediator of RNA polymerase II transcription subunit 17 | *med17* | 2.182 | 0.002 |
| Glucosamine-6-phosphate isomerase 1 | *gnpda1* | 2.180 | 0.005 |
| Translation initiation factor eIF-2B subunit alpha | *eif2b1* | 2.179 | 0.004 |
| 39S ribosomal protein L45, mitochondrial | *mrpl45* | 2.179 | 0.001 |
| UBX domain-containing protein 2A | *ubxn2a* | 2.167 | 0.001 |
| Ufm1-specific protease 2 | *ufsp2* | 2.167 | 0.001 |
| E3 ubiquitin-protein ligase MYLIP | *mylip* | 2.166 | 0.001 |
| Partitioning defective 6 homolog gamma | *pard6g* | 2.166 | 0.002 |
| ATP-dependent RNA helicase DDX55 | *ddx55* | 2.165 | 0.001 |
| Mediator of RNA polymerase II transcription subunit 27 | *med27* | 2.162 | 0.008 |
| Calcium release-activated calcium channel protein 1 | *orai1* | 2.160 | 0.003 |
| Densityregulated protein | *denr* | 2.159 | 0.001 |
| Im:7145112 protein | *elac2* | 2.158 | 0.002 |
| 28S ribosomal protein S26, mitochondrial | *mrps26* | 2.149 | 0.002 |
| ADP-ribosylation factor-like protein 6-interacting protein 1 | *arl6ip1* | 2.149 | 0.002 |
| IP05929p | *cg14903* | 2.145 | 0.002 |
| Inositol monophosphatase 1 | *impa1* | 2.145 | 0.000 |
| Transcription factor p65 | *rela* | 2.143 | 0.005 |
| Cryptochrome-1 | *cry1* | 2.140 | 0.001 |
| tRNA pseudouridine synthase A, mitochondrial | *pus1* | 2.134 | 0.005 |
| Carboxypeptidase Z | *cpz* | 2.133 | 0.001 |
| CAAX prenyl protease 1 homolog | *zmpste24* | 2.132 | 0.001 |
| Mitochondrial Rho GTPase 1 | *rhot1* | 2.132 | 0.002 |
| Propionyl-CoA carboxylase beta chain, mitochondrial | *pccb* | 2.132 | 0.002 |
| Endonuclease 8-like 1 | *neil1* | 2.131 | 0.001 |
| Target of rapamycin complex 2 subunit MAPKAP1 | *mapkap1* | 2.113 | 0.000 |
| DNA polymerase beta | *polb* | 2.113 | 0.002 |
| Zinc finger CCHC domain-containing protein 10 | *zcchc10* | 2.109 | 0.002 |
| Glycerol-3-phosphate acyltransferase 3 | *agpat9* | 2.108 | 0.000 |
| Nucleolar complex protein 4 homolog | *noc4l* | 2.107 | 0.002 |
| Interferon-related developmental regulator 1 | *ifrd1* | 2.107 | 0.002 |
| Mediator of RNA polymerase II transcription subunit 20 | *med20* | 2.105 | 0.006 |
| Histone-lysine N-methyltransferase SMYD3 | *smyd3* | 2.105 | 0.002 |
| Putative protein MSS51 homolog, mitochondrial | *mss51* | 2.105 | 0.003 |
| Peroxisomal membrane protein 11A | *pex11a* | 2.104 | 0.002 |
| Unknown | *?* | 2.102 | 0.002 |
| Ras GTPase-activating-like protein IQGAP2 | *iqgap2* | 2.101 | 0.004 |
| Pre-mRNA-splicing factor RBM22 | *rbm22* | 2.089 | 0.008 |
| Ubiquitin-like protein 4A | *ubl4a* | 2.089 | 0.002 |
| Ribonuclease P protein subunit p38 | *rpp38* | 2.088 | 0.008 |
| V-type proton ATPase subunit G 1 | *atp6v* | 2.087 | 0.001 |
| 3-oxoacyl-[acyl-carrier-protein] synthase, mitochondrial | *oxsm* | 2.085 | 0.003 |
| tRNA wybutosine-synthesizing protein 3 homolog | *tyw3* | 2.084 | 0.004 |
| Heat shock 70 kDa protein 14 | *hspa14* | 2.082 | 0.000 |
| 39S ribosomal protein L18 | *rpl18* | 2.081 | 0.000 |
| DNA-(apurinic or apyrimidinic site) lyase | *apex1* | 2.077 | 0.007 |
| Ribonuclease P protein subunit p40 | *rpp40* | 2.077 | 0.002 |
| UMP-CMP kinase 2, mitochondrial | *cmpk2* | 2.074 | 0.004 |
| Histone-lysine N-methyltransferase SETD7 | *setd7* | 2.071 | 0.005 |
| Tight junction protein ZO-2 | *tjp2* | 2.070 | 0.002 |
| Zinc finger protein 277 | *znf277* | 2.067 | 0.001 |
| 2',3'-cyclic-nucleotide 3'-phosphodiesterase | *cnp* | 2.064 | 0.006 |
| Transcription factor BTF3 | *btf3* | 2.061 | 0.002 |
| 5-hydroxytryptamine receptor 5A | *htr5a* | 2.061 | 0.002 |
| HRAS-like suppressor 3 | *pla2g16* | 2.052 | 0.002 |
| Anoctamin-10 | *ano10* | 2.051 | 0.003 |
| Zinc transporter ZIP12 | *slc39a* | 2.051 | 0.002 |
| 39S ribosomal protein L9, mitochondrial | *rpl9* | 2.049 | 0.004 |
| Type-1 angiotensin II receptor-associated protein | *agtrap* | 2.045 | 0.007 |
| Cell differentiation protein RCD1 homolog | *rcd1* | 2.038 | 0.002 |
| DNA polymerase kappa | *polk* | 2.038 | 0.003 |
| RNA-binding protein NOB1 | *nob1* | 2.037 | 0.007 |
| 26S proteasome non-ATPase regulatory subunit 10 | *psmd10* | 2.037 | 0.002 |
| Unknown | *?* | 2.036 | 0.009 |
| NADH dehydrogenase [ubiquinone] 1 alpha subcomplex assembly factor 3 | *ndufaf3* | 2.034 | 0.003 |
| Selenoprotein T | *selt* | 2.034 | 0.002 |
| Pleiotrophin | *ptn* | 2.029 | 0.002 |
| Zgc:162641 | *slc25a17* | 2.029 | 0.001 |
| Cathepsin C | *ctsc* | 2.029 | 0.007 |
| HIG1 domain family member 2A | *higd2a* | 2.029 | 0.002 |
| Transmembrane protein 147 | *tmem147* | 2.025 | 0.006 |
| Inorganic pyrophosphatase | *ppa1* | 2.024 | 0.001 |
| 39S ribosomal protein L22, mitochondrial | *mrpl22* | 2.024 | 0.002 |
| 39S ribosomal protein L4 | *rpl4* | 2.017 | 0.003 |
| DNA damage-binding protein 2 | *ddb2* | 2.010 | 0.004 |
| ATP synthase F(0) complex subunit C1, mitochondrial | *atp5g1* | 2.007 | 0.001 |
| OX-2 membrane glycoprotein | *cd200* | 2.007 | 0.004 |
| Tether containing UBX domain for GLUT4 | *aspscr1* | 2.006 | 0.000 |
| Methyltransferase-like protein 4 | *mettl4* | 2.004 | 0.005 |
| Ubiquitin-like protein 7 | *ubl7* | 2.004 | 0.000 |
| Mitochondrial thiamine pyrophosphate carrier | *slc25a19* | 2.004 | 0.000 |
| NADH dehydrogenase (Ubiquinone) Fe-S protein 3 (Predicted), isoform CRA_c | *ndufs3* | 2.004 | 0.005 |
| TIP41-like protein | *tiprl* | 2.003 | 0.002 |
| Methionine aminopeptidase 1D, mitochondrial | *metap1d* | 2.002 | 0.001 |
| Mitochondrial inner membrane protein OXA1L | *oxa1l* | 1.999 | 0.003 |
| NTF2-related export protein 1 | *nxt1* | 1.996 | 0.006 |
| Cathepsin B | *ctsb* | 1.996 | 0.008 |
| Ankyrin repeat and SOCS box protein 13 | *asb13* | 1.995 | 0.008 |
| Nucleolar protein 16 | *nop16* | 1.992 | 0.002 |
| Cytochrome b-c1 complex subunit 8 | *uqcrq* | 1.992 | 0.006 |
| 60S ribosomal protein L24 | *rpl24* | 1.990 | 0.000 |
| 60 kDa heat shock protein, mitochondrial | *hspd1* | 1.988 | 0.003 |
| EH domain-containing protein 1 | *ehd1* | 1.987 | 0.008 |
| DDB1- and CUL4-associated factor 13 | *dcaf13* | 1.986 | 0.005 |
| SET and MYND domain-containing protein 5 | *smyd5* | 1.983 | 0.001 |
| BAG family molecular chaperone regulator 1 | *bag1* | 1.983 | 0.002 |
| Acidic fibroblast growth factor intracellular-binding protein | *fibp* | 1.981 | 0.002 |
| 39S ribosomal protein L52, mitochondrial | *mrpl52* | 1.981 | 0.002 |
| RNA-binding protein 39 | *rbm39* | 1.976 | 0.008 |
| U5 small nuclear ribonucleoprotein 40 kDa protein | *snrnp40* | 1.967 | 0.002 |
| Nicotinate phosphoribosyltransferase | *naprt1* | 1.965 | 0.001 |
| Solute carrier family 25 member 40 | *slc25a40* | 1.965 | 0.004 |
| 39S ribosomal protein L5 | *rpl5* | 1.962 | 0.007 |
| Betaine aldehyde dehydrogenase | *aldh9a* | 1.962 | 0.006 |
| Rab5 GDP/GTP exchange factor | *rabgef1* | 1.961 | 0.002 |
| Proteasome activator complex subunit 1 | *psme1* | 1.958 | 0.000 |
| Mitochondrial intermediate peptidase | *mipep* | 1.958 | 0.002 |
| Serine/threonine-protein phosphatase 2A | *ppp2r2c* | 1.958 | 0.002 |
| ES1 protein homolog, mitochondrial | *c21orf33* | 1.957 | 0.005 |
| Cirhin | *cirh1a* | 1.956 | 0.002 |
| ATP synthase subunit g, mitochondrial | *atp5l* | 1.954 | 0.006 |
| Protein phosphatase methylesterase 1 | *ppme1* | 1.954 | 0.010 |
| Mortality factor 4-like protein 1 | *morf4l1* | 1.953 | 0.001 |
| m7GpppX diphosphatase | *dcps* | 1.952 | 0.009 |
| Myocardial zonula adherens protein | *gcom1* | 1.949 | 0.003 |
| 39S ribosomal protein L49, mitochondrial | *mrpl49* | 1.949 | 0.008 |
| Receptor-type tyrosine-protein phosphatase zeta | *ptprz1* | 1.949 | 0.005 |
| Low-density lipoprotein receptor | *ldlr* | 1.948 | 0.005 |
| Unknown | *?* | 1.945 | 0.003 |
| Ephrin type-A receptor 2 | *epha2* | 1.945 | 0.006 |
| 39S ribosomal protein L30 | *rpl30* | 1.944 | 0.000 |
| ADP-sugar pyrophosphatase | *nudt5* | 1.943 | 0.002 |
| Glutaredoxin-related protein 5, mitochondrial | *glrx5* | 1.942 | 0.003 |
| Probable ATP-dependent RNA helicase DHX37 | *dhx37* | 1.939 | 0.003 |
| Unknown | *?* | 1.934 | 0.002 |
| Histone H2A | *h2afv* | 1.931 | 0.008 |
| TATA box-binding protein-associated factor RNA polymerase I subunit A | *taf1a* | 1.927 | 0.001 |
| Unknown | *?* | 1.925 | 0.001 |
| Inosine triphosphate pyrophosphatase | *itpa* | 1.919 | 0.003 |
| 39S ribosomal protein L13 | *rpl13* | 1.919 | 0.001 |
| Glycosylphosphatidylinositol anchor attachment 1 protein | *gpaa1* | 1.918 | 0.003 |
| Bis(5'-nucleosyl)-tetraphosphatase [asymmetrical] | *nudt2* | 1.917 | 0.003 |
| Cleavage and polyadenylation specificity factor subunit 5 | *nudt21* | 1.914 | 0.004 |
| Heat shock cognate 71 kDa protein | *hspa8* | 1.914 | 0.003 |
| Lysophospholipase-like protein 1 | *lyplal1* | 1.913 | 0.003 |
| SH3 domain-binding glutamic acid-rich-like protein | *sh3bgrl* | 1.913 | 0.004 |
| tRNA-dihydrouridine(47) synthase [NAD(P)(+)]-like | *dus3l* | 1.908 | 0.007 |
| Geranylgeranyl transferase type-2 subunit beta | *rabggtb* | 1.903 | 0.002 |
| 40S ribosomal protein S17 | *rps17* | 1.903 | 0.000 |
| Caspase-1 | *casp1* | 1.903 | 0.005 |
| Histidine triad nucleotide-binding protein 3 | *hint3* | 1.898 | 0.002 |
| Mitochondrial ribonuclease P protein 3 | *kiaa0391* | 1.898 | 0.001 |
| WD repeat-containing protein 41 | *wdr41* | 1.896 | 0.001 |
| Splicing factor 45 | *rbm17* | 1.888 | 0.004 |
| Protein VAC14 homolog | *vac14* | 1.888 | 0.002 |
| Reverse transcriptase-like protein | *rtl* | 1.885 | 0.000 |
| Glutathione S-transferase Mu 1 | *gstm1* | 1.884 | 0.001 |
| GTPase Era, mitochondrial | *eral1* | 1.883 | 0.003 |
| Large neutral amino acids transporter small subunit 2 | *slc7a8* | 1.880 | 0.002 |
| Riboflavin kinase | *rfk* | 1.880 | 0.006 |
| Alpha/beta hydrolase domain-containing protein 14A | *abhd14a* | 1.880 | 0.002 |
| Probable tRNA N6-adenosine threonylcarbamoyltransferase, mitochondrial | *osgepl1* | 1.876 | 0.005 |
| Pericentrin | *pcnt* | 1.875 | 0.006 |
| Ribosome production factor 1 | *rpf1* | 1.875 | 0.005 |
| ATP-dependent RNA helicase DDX56 | *ddx56* | 1.862 | 0.002 |
| Cytoplasmic dynein 1 intermediate chain 2 | *dync1i2* | 1.862 | 0.001 |
| cAMP-dependent protein kinase inhibitor alpha | *pkia* | 1.860 | 0.004 |
| Cytoskeleton-associated protein 2 | *ckap2* | 1.859 | 0.007 |
| Leukocyte immune-type receptor TS32.15 L1.1a | *?* | 1.858 | 0.009 |
| Protein FAM46A | *fam46a* | 1.854 | 0.002 |
| Hydroxyacylglutathione hydrolase, mitochondrial | *hagh* | 1.853 | 0.002 |
| STON1-GTF2A1L protein | *ston1-gtf2a1l* | 1.852 | 0.005 |
| Glycine cleavage system H protein, mitochondrial | *gcsh* | 1.850 | 0.001 |
| Unknown | *?* | 1.850 | 0.007 |
| Steroid receptor RNA activator 1 | *sra1* | 1.847 | 0.005 |
| Anaphase-promoting complex subunit 7 | *anapc7* | 1.846 | 0.003 |
| Rho-associated protein kinase 2 | *rock2* | 1.844 | 0.007 |
| Translation initiation factor IF-3, mitochondrial | *mtif3* | 1.844 | 0.000 |
| Probable E3 ubiquitin-protein ligase HERC3 | *herc3* | 1.844 | 0.001 |
| NEDD8-activating enzyme E1 catalytic subunit | *uba3* | 1.844 | 0.001 |
| Endoplasmin | *hsp90b1* | 1.843 | 0.004 |
| Poly(rC)-binding protein 2 | *pcbp2* | 1.842 | 0.002 |
| Protein-tyrosine sulfotransferase 1 | *tpst1* | 1.842 | 0.000 |
| Glycogen phosphorylase, muscle form | *pygm* | 1.841 | 0.009 |
| ADM | *adm* | 1.839 | 0.004 |
| Endoplasmic reticulum-Golgi intermediate compartment protein 2 | *ergic2* | 1.838 | 0.001 |
| Tripeptidyl-peptidase 1 | *tpp1* | 1.835 | 0.004 |
| CD82 antigen | *cd82* | 1.834 | 0.002 |
| Signal recognition particle subunit SRP72 | *srp72* | 1.833 | 0.003 |
| 39S ribosomal protein L28, mitochondrial | *mrpl28* | 1.833 | 0.002 |
| Nitrogen permease regulator 3-like protein | *nprl3* | 1.826 | 0.003 |
| 40S ribosomal protein S18 | *rps18* | 1.826 | 0.000 |
| Eukaryotic initiation factor 4A-I | *eif4a1* | 1.825 | 0.003 |
| TNFAIP3-interacting protein 1 | *tnip1* | 1.824 | 0.002 |
| Polycomb group RING finger protein 2 | *pcgf2* | 1.822 | 0.001 |
| Pre-mRNA-splicing factor SPF27 | *bcas2* | 1.822 | 0.009 |
| Exosome complex exonuclease rrp4 | *exos2* | 1.821 | 0.010 |
| Arylamine N-acetyltransferase 1 | *nat1* | 1.819 | 0.001 |
| Ankyrin repeat domain-containing protein 16 | *ankrd16* | 1.817 | 0.002 |
| Phosphatidylinositol-glycan biosynthesis class F protein | *pigf* | 1.815 | 0.002 |
| Homeodomain-interacting protein kinase 1 | *hipk1* | 1.814 | 0.004 |
| Transferrin receptor protein 1 | *tfrc* | 1.813 | 0.001 |
| Phosphatidylinositol N-acetylglucosaminyltransferase subunit H | *pigh* | 1.810 | 0.009 |
| Ribonucleases P/MRP protein subunit POP1 | *pop1* | 1.810 | 0.009 |
| Phosphatidylinositol N-acetylglucosaminyltransferase subunit A | *piga* | 1.809 | 0.002 |
| tRNA methyltransferase 112 homolog | *trmt112* | 1.809 | 0.000 |
| Nucleolar protein 14 | *nop14* | 1.809 | 0.001 |
| Conserved oligomeric Golgi complex subunit 4 | *cog4* | 1.806 | 0.004 |
| Eukaryotic translation initiation factor 6 | *eif6* | 1.803 | 0.001 |
| Protein GTLF3B | *gtlf3b* | 1.802 | 0.010 |
| Golgi SNAP receptor complex member 2 | *gosr2* | 1.799 | 0.001 |
| Inhibitor of growth protein 1 | *ing1* | 1.798 | 0.010 |
| Nuclear receptor coactivator 1 | *ncoa1* | 1.798 | 0.000 |
| Hypoxanthine-guanine phosphoribosyltransferase | *hprt1* | 1.796 | 0.008 |
| E3 ubiquitin-protein ligase RAD18 | *rad18* | 1.795 | 0.002 |
| Sodium bicarbonate cotransporter | *nbc* | 1.794 | 0.002 |
| C-X-C chemokine receptor type 2 | *cxcr2* | 1.793 | 0.002 |
| Protein arginine N-methyltransferase 5 | *prmt5* | 1.790 | 0.004 |
| Histone acetyltransferase KAT8 | *kat8* | 1.790 | 0.002 |
| Dipeptidyl peptidase 2 | *dpp2* | 1.789 | 0.003 |
| WD40 repeat-containing protein SMU1 | *smu1* | 1.788 | 0.006 |
| Band 4.1-like protein 3 | *epb41l3* | 1.787 | 0.004 |
| Probable ATP-dependent RNA helicase DDX49 | *ddx49* | 1.786 | 0.002 |
| Serine incorporator 3 | *serinc3* | 1.785 | 0.002 |
| Coiled-coil-helix-coiled-coil-helix domain-containing protein 2, mitochondrial | *chchd2* | 1.782 | 0.008 |
| Adenylosuccinate synthetase isozyme 2 | *adss* | 1.782 | 0.006 |
| mRNA-capping enzyme | *rngtt* | 1.781 | 0.002 |
| 28S ribosomal protein S29, mitochondrial | *dap3* | 1.778 | 0.002 |
| Sodium-dependent neutral amino acid transporter SLC6A17 | *slc6a17* | 1.778 | 0.004 |
| Putative uncharacterized protein DKFZp686D02116 | *dkfzp686d02116* | 1.777 | 0.001 |
| SPARC | *sparc* | 1.776 | 0.002 |
| T-complex protein 1 subunit beta | *cct2* | 1.776 | 0.005 |
| FAD synthase | *flad1* | 1.771 | 0.008 |
| V-type proton ATPase subunit F | *atp6v* | 1.768 | 0.002 |
| UPF0511 protein C2orf56-like protein, mitochondrial | *eag_10011* | 1.767 | 0.001 |
| Hyaluronidase-1 | *hyal1* | 1.766 | 0.001 |
| Methyltransferase-like protein 2B | *mettl2b* | 1.762 | 0.004 |
| Ribonucleoside-diphosphate reductase subunit M2 B | *rrm2b* | 1.758 | 0.001 |
| Elongation factor Ts, mitochondrial | *tsfm* | 1.758 | 0.002 |
| tRNA pseudouridine synthase-like 1 | *pusl1* | 1.758 | 0.001 |
| MAD2L1-binding protein | *mad2l1bp* | 1.756 | 0.001 |
| 40S ribosomal protein S15 | *rps15* | 1.754 | 0.000 |
| 40S ribosomal protein S3 | *rps3* | 1.754 | 0.002 |
| LanC-like protein 1 | *lancl1* | 1.753 | 0.009 |
| Ras-related protein Rap-2b | *rap2b* | 1.751 | 0.009 |
| Derlin-2 | *derl2* | 1.751 | 0.004 |
| Dehydrogenase/reductase SDR family member 7B | *dhrs7b* | 1.749 | 0.008 |
| SH2 domain-containing protein 5 | *sh2d5* | 1.748 | 0.001 |
| Transmembrane protein 60 | *tmem60* | 1.747 | 0.003 |
| Molybdopterin synthase sulfur carrier subunit | *mocs2* | 1.747 | 0.003 |
| Dystrophin | *dmd* | 1.745 | 0.005 |
| THO complex subunit 4 | *alyref* | 1.744 | 0.008 |
| C-X-C motif chemokine | *cxcl* | 1.739 | 0.003 |
| Protein FAM76B | *fam76b* | 1.739 | 0.002 |
| Tail-anchored protein insertion receptor WRB | *wrb* | 1.739 | 0.006 |
| Zinc finger protein 330 | *znf330* | 1.738 | 0.005 |
| RISC-loading complex subunit TARBP2 | *tarbp2* | 1.734 | 0.004 |
| Peroxisomal biogenesis factor 19 | *pex19* | 1.733 | 0.000 |
| Nucleolar protein 58 | *nop58* | 1.732 | 0.007 |
| PRELI domain-containing protein 1, mitochondrial | *prelid1* | 1.732 | 0.001 |
| WD repeat-containing protein 13 | *wdr13* | 1.730 | 0.002 |
| EKC/KEOPS complex subunit TPRKB | *tprkb* | 1.730 | 0.001 |
| Transcription termination factor, mitochondrial | *mterf* | 1.729 | 0.004 |
| cAMPregulated phosphoprotein 21 | *arpp21* | 1.727 | 0.002 |
| Osteopetrosis-associated transmembrane protein 1 | *ostm1* | 1.727 | 0.004 |
| Protein TEX261 | *tex261* | 1.724 | 0.002 |
| 39S ribosomal protein L19 | *rpl19* | 1.724 | 0.000 |
| Alpha-soluble NSF attachment protein | *napa* | 1.723 | 0.003 |
| Adenylate cyclase type 2 | *adcy2* | 1.723 | 0.002 |
| Transcription factor Sp9 | *sp9* | 1.722 | 0.002 |
| THO complex subunit 7 homolog | *thoc7* | 1.721 | 0.008 |
| Ras-related protein Rab-9A | *rab9a* | 1.720 | 0.006 |
| V-type proton ATPase 16 kDa proteolipid subunit | *atp6v* | 1.717 | 0.008 |
| Radical S-adenosyl methionine domain-containing protein 2 | *rsad2* | 1.717 | 0.000 |
| Tetratricopeptide repeat protein 27 | *ttc27* | 1.717 | 0.002 |
| 3-hydroxyisobutyryl-CoA hydrolase, mitochondrial | *hibch* | 1.717 | 0.002 |
| Ras-related protein Rap-1A | *rap1a* | 1.715 | 0.006 |
| Transcription elongation factor SPT4-B | *supt4h1b* | 1.710 | 0.002 |
| Transmembrane protein 69 | *tmem69* | 1.710 | 0.007 |
| Estradiol 17-beta-dehydrogenase 12 | *hsd17b12* | 1.709 | 0.010 |
| ATP-dependent RNA helicase DDX18 | *ddx18* | 1.709 | 0.009 |
| N-lysine methyltransferase SETD6 | *setd6* | 1.708 | 0.005 |
| Enteropeptidase | *tmprss15* | 1.705 | 0.009 |
| Lactosylceramide alpha-2,3-sialyltransferase | *st3gal5* | 1.705 | 0.004 |
| Legumain | *lgmn* | 1.704 | 0.003 |
| DNA-directed RNA polymerase III subunit RPC9 | *crcp* | 1.703 | 0.002 |
| Junction plakoglobin | *jup* | 1.703 | 0.001 |
| AP-3 complex subunit sigma-2 | *ap3s2* | 1.702 | 0.004 |
| E3 ubiquitin-protein ligase TRIM13 | *trim13* | 1.702 | 0.002 |
| DnaJ homolog subfamily C member 11 | *dnajc11* | 1.702 | 0.004 |
| RING finger protein 121 | *rnf121* | 1.702 | 0.004 |
| AN1-type zinc finger protein 1 | *zfand1* | 1.701 | 0.009 |
| Zinc finger protein 706 | *znf706* | 1.699 | 0.008 |
| Histone deacetylase 11 | *hdac11* | 1.699 | 0.009 |
| Purine nucleoside phosphorylase | *pnp* | 1.697 | 0.000 |
| Platelet-activating factor acetylhydrolase IB subunit gamma | *pafah1b3* | 1.695 | 0.006 |
| 28S ribosomal protein S17, mitochondrial | *mrps17* | 1.693 | 0.010 |
| Protein LZIC | *lzic* | 1.693 | 0.006 |
| Ubiquinone biosynthesis monooxygenase COQ6 | *coq6* | 1.692 | 0.008 |
| Growth hormoneregulated TBC protein 1 | *grtp1* | 1.692 | 0.004 |
| Receptor-type tyrosine-protein phosphatase alpha | *ptpra* | 1.688 | 0.009 |
| Glutamine-rich protein 1 | *qrich1* | 1.688 | 0.008 |
| Keratinocyte-associated protein 2 | *krtcap2* | 1.687 | 0.010 |
| Homeobox and leucine zipper protein Homez | *homez* | 1.687 | 0.006 |
| Tubulin alpha-1B chain | *tuba1b* | 1.686 | 0.006 |
| Mucin-2 | *muc2* | 1.686 | 0.000 |
| Trimethylguanosine synthase | *tgs1* | 1.686 | 0.010 |
| Galactocerebrosidase | *galc* | 1.686 | 0.005 |
| Cat eye syndrome critical region protein 5 | *cecr5* | 1.686 | 0.005 |
| MKI67 FHA domain-interacting nucleolar phosphoprotein | *nifk* | 1.686 | 0.004 |
| E3 ubiquitin-protein ligase RNF126 | *rnf126* | 1.685 | 0.006 |
| Golgin subfamily A member 5 | *golga5* | 1.684 | 0.001 |
| Palmitoyltransferase ZDHHC23 | *zdhhc23* | 1.683 | 0.002 |
| Probable ATP-dependent RNA helicase DHX8 | *dhx8* | 1.682 | 0.008 |
| Coatomer subunit epsilon | *cope* | 1.681 | 0.008 |
| Wu:fj80h11 protein | *si:dkey-256h2.1* | 1.678 | 0.005 |
| Kynurenine--oxoglutarate transaminase 1 | *ccbl1* | 1.676 | 0.007 |
| Transmembrane channel-like protein | *tmc6* | 1.674 | 0.003 |
| Ras-related protein R-Ras2 | *rras2* | 1.674 | 0.001 |
| Elongation factor G, mitochondrial | *gfm1* | 1.672 | 0.003 |
| Patatin-like phospholipase domain-containing protein 2 | *pnpla2* | 1.671 | 0.002 |
| Alpha-2C adrenergic receptor | *adra2c* | 1.671 | 0.002 |
| UPF0402 protein | *egm_09475* | 1.670 | 0.005 |
| Protein BANP | *banp* | 1.669 | 0.001 |
| Eukaryotic translation initiation factor 3 subunit A | *eif3a* | 1.666 | 0.003 |
| Tyrosine--tRNA ligase, mitochondrial | *yars2* | 1.659 | 0.006 |
| UNC119-binding protein C5orf30 homolog | *d1ertd622e* | 1.659 | 0.004 |
| C1GALT1-specific chaperone 1 | *c1galt1c1* | 1.657 | 0.005 |
| Nucleoside diphosphate kinase 6 | *nme6* | 1.657 | 0.009 |
| Threonine--tRNA ligase, cytoplasmic | *tars* | 1.657 | 0.006 |
| Polymerase delta-interacting protein 2 | *poldip2* | 1.656 | 0.004 |
| Serine/threonine-protein kinase A-Raf | *araf* | 1.654 | 0.004 |
| Calcium uptake protein 2, mitochondrial | *micu2* | 1.652 | 0.010 |
| COMM domain-containing protein 3 | *commd3* | 1.652 | 0.002 |
| Cytoplasmic dynein 1 intermediate chain 2 | *dync1i2* | 1.651 | 0.002 |
| WD repeat-containing protein 6 | *wdr6* | 1.647 | 0.002 |
| E3 ubiquitin-protein ligase RNF146 | *rnf146* | 1.647 | 0.002 |
| 39S ribosomal protein L20, mitochondrial | *mrpl20* | 1.643 | 0.009 |
| Septin-2 | *sept2* | 1.643 | 0.009 |
| Fanconi anemia group F protein | *fancf* | 1.641 | 0.006 |
| Serine/threonine/tyrosine-interacting protein | *styx* | 1.641 | 0.005 |
| Mesoderm-specific transcript homolog protein | *mest* | 1.639 | 0.004 |
| Tensin-4 | *tns4* | 1.638 | 0.002 |
| HCLS1-associated protein X-1 | *hax1* | 1.634 | 0.004 |
| Dynamin-1-like protein | *dnm1l* | 1.634 | 0.004 |
| Nuclear transcription factor Y subunit alpha | *nfya* | 1.633 | 0.003 |
| Golgin subfamily B member 1 | *golgb1* | 1.633 | 0.009 |
| Tetratricopeptide repeat protein 17 | *ttc17* | 1.632 | 0.005 |
| 40S ribosomal protein S8 | *rps8* | 1.631 | 0.000 |
| Transcription factor 25 | *tcf25* | 1.631 | 0.001 |
| UDP-glucose 4-epimerase | *gale* | 1.626 | 0.007 |
| 40S ribosomal protein S30 | *rps30* | 1.626 | 0.003 |
| Pre-rRNA-processing protein TSR1 homolog | *tsr1* | 1.625 | 0.008 |
| Phosphoglucomutase-2 | *pgm2* | 1.623 | 0.002 |
| Eukaryotic translation initiation factor 4E-binding protein 1 | *eif4ebp1* | 1.622 | 0.009 |
| Translationally-controlled tumor protein | *tpt1* | 1.622 | 0.008 |
| Nicotinamide mononucleotide adenylyltransferase 3 | *nmnat3* | 1.622 | 0.001 |
| ATPase family AAA domain-containing protein 1-B | *atad1b* | 1.621 | 0.003 |
| Protein arginine N-methyltransferase 7 | *prmt7* | 1.619 | 0.008 |
| Presequence protease, mitochondrial | *pitrm1* | 1.618 | 0.003 |
| Ribosomal protein S6 kinase alpha-1 | *rps6ka1* | 1.615 | 0.004 |
| Plakophilin-2 | *pkp2* | 1.615 | 0.001 |
| tRNA (guanine-N(7)-)-methyltransferase non-catalytic subunit WDR4 | *wdr4* | 1.614 | 0.005 |
| Cell cycle control protein 50A | *tmem30a* | 1.613 | 0.001 |
| Syntaxin-5 | *stx5* | 1.612 | 0.005 |
| Glutathione synthetase | *gss* | 1.611 | 0.008 |
| CG13731 | *dmel_cg13731* | 1.610 | 0.002 |
| Cytochrome b561 domain-containing protein 2 | *cyb561d2* | 1.610 | 0.003 |
| Ecto-NOX disulfide-thiol exchanger 1 | *enox1* | 1.607 | 0.002 |
| COMM domain-containing protein 2 | *commd2* | 1.606 | 0.002 |
| Tetratricopeptide repeat protein 39C | *ttc39c* | 1.606 | 0.008 |
| Mannose-1-phosphate guanyltransferase alpha | *gmppa* | 1.604 | 0.001 |
| Proteasome subunit alpha type-6 | *psma6* | 1.599 | 0.006 |
| SRSF protein kinase 1 | *srpk1* | 1.599 | 0.002 |
| ER lumen protein retaining receptor 2 | *kdelr2* | 1.598 | 0.005 |
| Cytochrome c oxidase assembly protein COX15 homolog | *cox15* | 1.596 | 0.009 |
| General transcription factor IIH subunit 3 | *gtf2h3* | 1.595 | 0.010 |
| Troponin C, skeletal muscle | *tnnc2* | 1.594 | 0.003 |
| Protein slowmo homolog 2 | *slmo2* | 1.594 | 0.003 |
| E3 ubiquitin-protein ligase DTX3L | *dtx3l* | 1.594 | 0.001 |
| Protein NEDD1 | *nedd1* | 1.593 | 0.002 |
| Arrestin domain-containing protein 3 | *arrdc3* | 1.593 | 0.006 |
| GTPase Era, mitochondrial | *eral1* | 1.592 | 0.002 |
| Putative humanin peptide | *mt-rnr2* | 1.590 | 0.002 |
| UPF0105 protein C14orf124 homolog | *cn124* | 1.590 | 0.003 |
| GDP-mannose 4,6 dehydratase | *gmds* | 1.588 | 0.001 |
| 39S ribosomal protein L18a | *rpl18a* | 1.584 | 0.001 |
| Cytochrome c oxidase protein 20 homolog | *cox20* | 1.584 | 0.005 |
| 40S ribosomal protein S14 | *rps14* | 1.583 | 0.000 |
| Copper homeostasis protein cutC homolog | *cutc* | 1.583 | 0.002 |
| Decapping and exoribonuclease protein | *dxo* | 1.580 | 0.003 |
| Protein FAM193B | *fam193b* | 1.579 | 0.009 |
| Cyclin-D1-binding protein 1 homolog | *ccndbp1* | 1.579 | 0.010 |
| Serine/threonine-protein kinase RIO2 | *riok2* | 1.578 | 0.005 |
| High mobility group protein 20A | *hmg20a* | 1.575 | 0.001 |
| TAF7 RNA polymerase II, TATA box binding protein (TBP)-associated factor | *taf7* | 1.574 | 0.003 |
| DNA-directed RNA polymerases I, II, and III subunit RPABC2 | *polr2f* | 1.572 | 0.002 |
| Polyubiquitin-C | *ubc* | 1.571 | 0.008 |
| Guanine nucleotide exchange factor MSS4 | *rabif* | 1.569 | 0.001 |
| Nucleolar complex protein 2 homolog | *noc2l* | 1.567 | 0.008 |
| GRB2-associated-binding protein 1 | *?* | 1.566 | 0.002 |
| Putative methyltransferase UPF0383 | *eag_01969* | 1.565 | 0.002 |
| Fatty-acid amide hydrolase 2-B | *faah2b* | 1.563 | 0.005 |
| Hyperosmotic glycine rich protein | *?* | 1.561 | 0.005 |
| Vacuolar ATP synthase 16 kDa proteolipid subunit-like protein | *?* | 1.559 | 0.008 |
| N-alpha-acetyltransferase 10 | *naa10* | 1.558 | 0.009 |
| General receptor for phosphoinositides 1-associated scaffold protein | *grasp* | 1.553 | 0.006 |
| Chromodomain-helicase-DNA-binding protein 1 | *chd1* | 1.553 | 0.003 |
| Cytosolic Fe-S cluster assembly factor NARFL | *narfl* | 1.551 | 0.009 |
| Serine/threonine-protein kinase B-raf | *braf* | 1.550 | 0.001 |
| 1-acylglycerol-3-phosphate O-acyltransferase ABHD5 | *abhd5* | 1.550 | 0.005 |
| Nucleolysin TIA-1 isoform p40 | *tia1* | 1.546 | 0.009 |
| AP-5 complex subunit mu-1 | *ap5m1* | 1.546 | 0.004 |
| Metaxin-3 | *mtx3* | 1.545 | 0.002 |
| Solute carrier family 25 member 43 | *slc25a43* | 1.543 | 0.003 |
| Vesicle transport through interaction with t-SNAREs homolog 1B | *vti1b* | 1.540 | 0.003 |
| E3 ubiquitin-protein ligase BRE1A | *rnf20* | 1.539 | 0.004 |
| Folliculin | *flcn* | 1.538 | 0.009 |
| Syntaxin-12 | *stx12* | 1.537 | 0.009 |
| Cyclin-dependent kinases regulatory subunit 1 | *cks1b* | 1.537 | 0.002 |
| 39S ribosomal protein L12 | *rpl12* | 1.537 | 0.004 |
| Charged multivesicular body protein 4c | *chmp4c* | 1.536 | 0.006 |
| Sorting and assembly machinery component 50 homolog | *samm50* | 1.535 | 0.010 |
| 39S ribosomal protein L13a | *rpl13a* | 1.534 | 0.000 |
| Serine/threonine-protein kinase Nek4 | *nek4* | 1.533 | 0.001 |
| Diphthamide biosynthesis protein 1 | *dph1* | 1.533 | 0.001 |
| U8 snoRNA-decapping enzyme | *nudt16* | 1.531 | 0.005 |
| Mitochondrial inner membrane protease ATP23 homolog | *xrcc6bp1* | 1.527 | 0.007 |
| COMM domain-containing protein 5 | *commd5* | 1.523 | 0.004 |
| Peroxisomal multifunctional enzyme type 2 | *hsd17b4* | 1.520 | 0.007 |
| Cytochrome P450 2C33-like | *?* | 1.519 | 0.007 |
| Grainyhead-like protein 2 homolog | *grhl2* | 1.519 | 0.007 |
| GPN-loop GTPase 3 | *gpn3* | 1.517 | 0.005 |
| Lysosome-associated membrane glycoprotein 1 | *lamp1* | 1.516 | 0.004 |
| 40S ribosomal protein S15a | *rps15a* | 1.509 | 0.003 |
| 39S ribosomal protein L23 | *rpl23* | 1.508 | 0.000 |
| Protein TBRG4 | *tbrg4* | 1.508 | 0.009 |
| Ribosome biogenesis protein BMS1 homolog | *bms1* | 1.507 | 0.008 |
| Ribosome maturation protein SBDS | *sbds* | 1.507 | 0.007 |
| DNA-directed RNA polymerases I, II, and III subunit RPABC5 | *polr2l* | 1.506 | 0.001 |
| ATP-dependent helicase | *?* | 1.504 | 0.004 |
| Band 4.1-like protein 3 | *epb41l3* | 1.502 | 0.003 |
| Ras-related protein Rab-11B | *rab11b* | 1.501 | 0.002 |
| 1-acyl-sn-glycerol-3-phosphate acyltransferase epsilon | *agpat5* | -13.376 | 0.000 |
| Fatty acid-binding protein, heart | *fabp3* | -11.365 | 0.005 |
| Propionyl-CoA carboxylase alpha chain, mitochondrial | *pcca* | -10.257 | 0.000 |
| Histone chaperone asf1-b | *as1lb* | -7.635 | 0.000 |
| 1-phosphatidylinositol 3-phosphate 5-kinase | *pikfyve* | -7.044 | 0.000 |
| Glutamate receptor ionotropic, kainate 4 | *grik4* | -6.743 | 0.000 |
| MORN repeat-containing protein 3 | *morn3* | -6.441 | 0.008 |
| Prolactin | *prl* | -6.355 | 0.000 |
| Short/branched chain specific acyl-CoA dehydrogenase, mitochondrial | *acadsb* | -6.107 | 0.000 |
| Unhealthy ribosome biogenesis protein 2 homolog | *urb2* | -5.794 | 0.001 |
| Unknown | *?* | -5.716 | 0.006 |
| NADH dehydrogenase [ubiquinone] 1 alpha subcomplex subunit 3 | *ndufa3* | -5.610 | 0.006 |
| Meiotic recombination protein DMC1/LIM15 homolog | *dmc1* | -5.456 | 0.010 |
| Exostosin-1 | *ext1* | -5.396 | 0.001 |
| E3 ubiquitin-protein ligase Midline-1 | *mid1* | -5.327 | 0.000 |
| Unknown | *?* | -5.228 | 0.003 |
| Transgelin | *tagln* | -5.224 | 0.009 |
| Ras-related protein O-RAL | *?* | -5.208 | 0.003 |
| Transposable element Tc3 transposase | *tc3a* | -5.009 | 0.003 |
| Unknown | *?* | -4.984 | 0.000 |
| Nucleoside diphosphate kinase | *nme7* | -4.871 | 0.002 |
| Actin-related protein 2/3 complex subunit 1A | *arpc1a* | -4.853 | 0.010 |
| Potassium voltage-gated channel subfamily C member 3 | *kcnc3* | -4.820 | 0.000 |
| Integrin beta-1 | *itgb1* | -4.773 | 0.008 |
| WD repeat domain phosphoinositide-interacting protein 1 | *wipi1* | -4.687 | 0.005 |
| Butyrophilin Like Protein 4 | *btnl4* | -4.669 | 0.002 |
| GPI-linked NAD(P)(+)--arginine ADP-ribosyltransferase 1 | *art1* | -4.494 | 0.000 |
| Mesothelin | *msln* | -4.428 | 0.002 |
| Eukaryotic peptide chain release factor GTP-binding subunit ERF3A | *gspt1* | -4.427 | 0.000 |
| Tetratricopeptide repeat protein 25 | *ttc25* | -4.294 | 0.003 |
| Unknown | *?* | -4.252 | 0.002 |
| Ephrin type-A receptor 2 | *epha2* | -4.248 | 0.002 |
| Lipopolysaccharide-induced tumor necrosis factor-alpha factor | *litaf* | -4.207 | 0.004 |
| PDZ and LIM domain protein 3 | *pdlim3* | -4.152 | 0.001 |
| JunDLa | *jundla* | -4.144 | 0.003 |
| Junctional adhesion molecule C | *jam3* | -4.068 | 0.002 |
| Cytochrome P450 aromatase brain isoform | *cyp19a2* | -4.027 | 0.001 |
| Unknown | *?* | -3.907 | 0.004 |
| Sodium-coupled neutral amino acid transporter 4 | *slc38a* | -3.804 | 0.009 |
| Probable G-protein coupled receptor 139 | *gpr139* | -3.736 | 0.002 |
| Tropomyosin alpha-1 chain | *tpm1* | -3.691 | 0.002 |
| cAMP-dependent protein kinase type I-alpha regulatory subunit | *prkar1a* | -3.685 | 0.000 |
| Unknown | *?* | -3.641 | 0.000 |
| Methionine-R-sulfoxide reductase B3 | *msrb3* | -3.598 | 0.008 |
| Dual specificity protein phosphatase 7 | *dusp7* | -3.561 | 0.002 |
| Sodium/potassium-transporting ATPase subunit gamma | *fxyd2* | -3.529 | 0.006 |
| Gap junction alpha-1 protein | *gja1* | -3.504 | 0.002 |
| A-kinase anchor protein 14 | *akap14* | -3.452 | 0.009 |
| Vacuolar protein sorting-associated protein 4B | *vps4b* | -3.423 | 0.004 |
| Peptidyl-prolyl cis-trans isomerase FKBP14 | *fkbp14* | -3.402 | 0.001 |
| DNA methyltransferase 1-associated protein 1 | *dmap1* | -3.366 | 0.004 |
| Phospholemman | *fxyd1* | -3.293 | 0.009 |
| Vacuolar protein sorting-associated protein 37A | *vps37a* | -3.288 | 0.008 |
| Spon2b protein | *spon2b* | -3.226 | 0.006 |
| Importin-7 | *ipo7* | -3.208 | 0.001 |
| Prostaglandin E synthase | *ptges* | -3.200 | 0.001 |
| Ionotropic glutamate recetor subunit 3 alpha | *fglur3a* | -3.175 | 0.000 |
| Uncharacterized protein | *psmd8* | -3.165 | 0.000 |
| S-phase kinase-associated protein 1 | *skp1* | -3.120 | 0.001 |
| Serine incorporator 2 | *serinc2* | -3.077 | 0.006 |
| Tetraspanin-5 | *tspan5* | -3.064 | 0.002 |
| Peptidyl-prolyl cis-trans isomerase FKBP1B | *fkbp1b* | -3.050 | 0.001 |
| Tropomyoshin1-1 | *tpm1-1* | -2.973 | 0.009 |
| NAD(P)H-hydrate epimerase | *apoa1bp* | -2.968 | 0.009 |
| Cytosolic Fe-S cluster assembly factor NUBP1 | *nubp1* | -2.966 | 0.002 |
| Renin | *ren* | -2.945 | 0.000 |
| Potassium voltage-gated channel subfamily E member 1-like protein | *kcne1l* | -2.900 | 0.001 |
| Embigin | *emb* | -2.894 | 0.003 |
| Dynamin-2 | *dnm2* | -2.883 | 0.010 |
| tRNA (cytosine(38)-C(5))-methyltransferase | *trdmt1* | -2.879 | 0.000 |
| Butyrophilin subfamily 1 member A1 | *btn1a1* | -2.852 | 0.000 |
| Transaldolase | *taldo1* | -2.850 | 0.003 |
| CD63 antigen | *cd63* | -2.837 | 0.010 |
| E3 ubiquitin-protein ligase TRIP12 | *trip12* | -2.835 | 0.003 |
| Vitronectin | *vtn* | -2.777 | 0.001 |
| Craniofacial development protein 1 | *cfdp1* | -2.774 | 0.001 |
| Transcriptionalregulating factor 1 | *trerf1* | -2.749 | 0.004 |
| Heat shock 70 kDa protein 4 | *hspa4* | -2.732 | 0.002 |
| Four and a half LIM domains protein 1 | *fhl1* | -2.708 | 0.007 |
| Voltage-dependent anion-selective channel protein 2 | *vdac2* | -2.706 | 0.007 |
| Cytoplasmic dynein 1 heavy chain 1 | *dync1h1* | -2.697 | 0.004 |
| Steroid hormone receptor ERR2 | *esrrb* | -2.685 | 0.005 |
| Unknown | *?* | -2.646 | 0.004 |
| Doublesex- and mab-3-related transcription factor 1 | *dmrt1* | -2.643 | 0.003 |
| Lysophosphatidic acid receptor 6 | *lpar6* | -2.641 | 0.002 |
| Unknown | *?* | -2.598 | 0.003 |
| Beta-2 microglobuli | *b2m* | -2.585 | 0.002 |
| Phosphoglucomutase-like protein 5 | *pgm5* | -2.547 | 0.005 |
| Prostaglandin reductase 1 | *ptgr1* | -2.543 | 0.001 |
| Syndecan 4 | *sdc4* | -2.537 | 0.008 |
| Hydroxycarboxylic acid receptor 2 | *hcar2* | -2.527 | 0.005 |
| Myelin proteolipid protein | *plp1* | -2.512 | 0.001 |
| 26S proteasome non-ATPase regulatory subunit 8 | *psmd8* | -2.493 | 0.001 |
| DAZ-associated protein 2 | *dazap2* | -2.446 | 0.007 |
| Peripheral plasma membrane protein CASK | *cask* | -2.445 | 0.001 |
| Unknown | *?* | -2.422 | 0.004 |
| Proteasome inhibitor PI31 subunit | *psmf1* | -2.421 | 0.004 |
| Intraflagellar transport protein 52 homolog | *ift52* | -2.417 | 0.003 |
| EF-hand calcium-binding domain-containing protein 7 | *efcab7* | -2.408 | 0.002 |
| Prostaglandin E synthase | *ptges* | -2.390 | 0.007 |
| Plexin-C1 | *plxnc1* | -2.379 | 0.000 |
| Negative elongation factor E | *nelfe* | -2.365 | 0.002 |
| Uncharacterized protein C21orf59-like protein | *cgi_10004163* | -2.348 | 0.006 |
| Tumor necrosis factor receptor superfamily member 1A | *tnfrsf1a* | -2.337 | 0.007 |
| Plasma membrane calcium-transporting ATPase 1 | *atp2b1* | -2.325 | 0.003 |
| Unknown | *?* | -2.317 | 0.009 |
| NFKBIL1 | *nfkbil1* | -2.306 | 0.007 |
| Transcription factor p65 | *rela* | -2.298 | 0.001 |
| Clathrin light chain A | *clta* | -2.293 | 0.001 |
| Ligand-dependent nuclear receptor-interacting factor 1 | *lrif1* | -2.289 | 0.007 |
| Protein-arginine deiminasae type II-like | *?* | -2.283 | 0.000 |
| Collagen type IV alpha-3-binding protein | *col4a3bp* | -2.262 | 0.003 |
| Eukaryotic initiation factor 4A-II | *eif4a2* | -2.257 | 0.001 |
| DnaJ homolog subfamily A member 4 | *dnaja4* | -2.254 | 0.008 |
| Granulins | *grn* | -2.241 | 0.004 |
| Carboxypeptidase N catalytic chain | *?* | -2.237 | 0.000 |
| Caspase-9 | *casp9* | -2.231 | 0.002 |
| DnaJ homolog subfamily B member 1 | *dnajb1* | -2.228 | 0.009 |
| ? | *?* | -2.206 | 0.005 |
| Peflin | *pef1* | -2.198 | 0.002 |
| ? | *?* | -2.193 | 0.008 |
| Leucine-rich repeat-containing protein 40 | *lrrc40* | -2.183 | 0.000 |
| Sugar phosphate exchanger 2 | *slc37a* | -2.181 | 0.010 |
| Cytochrome P450 aromatase brain isoform | *cyp19a2* | -2.170 | 0.006 |
| Protein RER1 | *rer1* | -2.169 | 0.006 |
| ? | *?* | -2.167 | 0.003 |
| sorting nexin variation 2 | *snx2* | -2.165 | 0.005 |
| Vesicle-associated membrane protein 3 | *vamp3* | -2.160 | 0.006 |
| Gelsolin | *gsn* | -2.155 | 0.003 |
| ? | *?* | -2.153 | 0.001 |
| Ornithine decarboxylase | *odc1* | -2.150 | 0.005 |
| Suppressor of cytokine signaling 4 | *socs4* | -2.147 | 0.003 |
| EGF-containing fibulin-like extracellular matrix protein 1 | *efemp1* | -2.144 | 0.001 |
| Golgi SNAP receptor complex member 1 | *gosr1* | -2.137 | 0.003 |
| Heterogeneous nuclear ribonucleoprotein R | *hnrnpr* | -2.134 | 0.003 |
| LYR motif-containing protein 5 | *lyrm5* | -2.126 | 0.009 |
| RB1-inducible coiled-coil protein 1 | *rb1cc1* | -2.124 | 0.007 |
| Glucocorticoid receptor | *nr3c1* | -2.098 | 0.003 |
| ? | *?* | -2.097 | 0.006 |
| Tetraspanin-18 | *tspan18* | -2.081 | 0.001 |
| Krueppel-like factor 13 | *klf13* | -2.066 | 0.000 |
| Calcium/calmodulin-dependent protein kinase type 1 | *camk1* | -2.064 | 0.007 |
| Prostaglandin E synthase 3 | *ptges3* | -2.061 | 0.005 |
| N-acetyl lactosaminide beta-1,3-N-acetyl glucosaminyl transferase | *cpipj_cpij017866* | -2.049 | 0.007 |
| Chromodomain-helicase-DNA-binding protein 1-like | *chd1l* | -2.041 | 0.002 |
| FXYD5b | *?* | -2.035 | 0.008 |
| Ubiquitin carboxyl-terminal hydrolase 33 | *usp33* | -2.035 | 0.002 |
| Long-chain-fatty-acid--CoA ligase 1 | *acsl1* | -2.033 | 0.002 |
| Transmembrane protein 131 | *tmem131* | -2.028 | 0.005 |
| B-cell CLL/lymphoma 7 protein family member B | *bcl7b* | -2.014 | 0.004 |
| RING finger protein 24 | *rnf24* | -2.011 | 0.010 |
| LIM domain-containing protein ajuba | *ajuba* | -2.011 | 0.003 |
| FBP32 | *?* | -2.007 | 0.004 |
| Syntaxin-binding protein 1 | *stxbp1* | -2.002 | 0.000 |
| Ras-specific guanine nucleotide-releasing factor RalGPS2 | *ralgps2* | -1.985 | 0.003 |
| Platelet glycoprotein IX | *gp9* | -1.984 | 0.003 |
| ? | *?* | -1.982 | 0.000 |
| Uncharacterized protein C10orf12 | *c10orf12* | -1.975 | 0.006 |
| Aldehyde dehydrogenase ALDH2b | *aldh2b* | -1.964 | 0.004 |
| Von Hippel-Lindau disease tumor suppressor | *vhl* | -1.961 | 0.008 |
| MAPK/MAK/MRK overlapping kinase | *mok* | -1.957 | 0.004 |
| Proteasome subunit beta type-1 | *psmb1* | -1.956 | 0.009 |
| GA20768 | *dpse\ga20768* | -1.956 | 0.002 |
| Serine/arginine-rich splicing factor 11 | *srsf11* | -1.931 | 0.009 |
| orphan nuclear receptor DAX2 | *nr0b1b* | -1.921 | 0.008 |
| Ubiquitin-conjugating enzyme E2 variant 2 | *ube2v2* | -1.920 | 0.002 |
| Ubqln4-prov protein | *ubqln4-prov* | -1.919 | 0.003 |
| Bifunctional apoptosis regulator | *bfar* | -1.918 | 0.004 |
| High mobility group protein B2 | *hmgb2* | -1.917 | 0.003 |
| Interleukin-1 receptor-like 1 | *il1rl1* | -1.911 | 0.002 |
| Reverse transcriptase-like protein | *rtl* | -1.910 | 0.006 |
| E3 ubiquitin-protein ligase RNF13 | *rnf13* | -1.910 | 0.007 |
| Eukaryotic translation initiation factor 4E | *eif4e* | -1.909 | 0.004 |
| 40S ribosomal protein S17 | *rps17* | -1.905 | 0.005 |
| GTP-binding nuclear protein Ran | *ran* | -1.902 | 0.006 |
| FYVE, RhoGEF and PH domain-containing protein 6 | *fgd6* | -1.900 | 0.004 |
| Guanine nucleotide-binding protein G(I)/G(S)/G(O) subunit gamma-13 | *gng13* | -1.899 | 0.007 |
| Ret finger protein-like 1 | *rfpl1* | -1.883 | 0.007 |
| 11-beta-hydroxysteroid dehydrogenase-like protein | *hsd11bl* | -1.882 | 0.003 |
| Receptor-type tyrosine-protein phosphatase S | *ptprs* | -1.875 | 0.004 |
| Glycine-gated ion channel alpha3 subunit | *?* | -1.873 | 0.007 |
| Transmembrane protein 107 | *tmem107* | -1.861 | 0.005 |
| ? | *?* | -1.860 | 0.004 |
| Serine/threonine-protein phosphatase 2A [≈ High power LED current, peak 2.7 A] regulatory subunit B'' subunit alpha | *ppp2r3a* | -1.857 | 0.002 |
| Dual specificity protein kinase CLK2 | *clk2* | -1.849 | 0.003 |
| G-protein coupled receptor 56 | *gpr56* | -1.847 | 0.007 |
| Gamma-tubulin complex component 2 | *tubgcp2* | -1.845 | 0.010 |
| Guanine nucleotide-binding protein G(I)/G(S)/G(O) subunit gamma-12 | *gng12* | -1.843 | 0.009 |
| V-type proton ATPase subunit a, Golgi isoform | *stv1* | -1.835 | 0.000 |
| Transforming protein RhoA | *rhoa* | -1.820 | 0.008 |
| Ephrin-A1 | *efna1* | -1.819 | 0.003 |
| Retinal rod rhodopsin-sensitive cGMP 3',5'-cyclic phosphodiesterase subunit gamma | *pde6g* | -1.817 | 0.006 |
| Sulfotransferase family 1, cytosolic sulfotransferase 6 | *sult1st6* | -1.817 | 0.004 |
| Serine protease HTRA1 | *htra1* | -1.812 | 0.001 |
| Sister chromatid cohesion protein PDS5 homolog A | *pds5a* | -1.810 | 0.000 |
| Transcriptional regulator ATRX | *atrx* | -1.809 | 0.009 |
| LON peptidase N-terminal domain and RING finger protein 1 | *lonrf1* | -1.806 | 0.003 |
| Microtubule-associated protein RP/EB family member 1 | *mapre1* | -1.797 | 0.007 |
| COP9 signalosome complex subunit 5 | *cops5* | -1.791 | 0.005 |
| Aldehyde dehydrogenase, mitochondrial | *aldh2* | -1.790 | 0.009 |
| Protein max | *max* | -1.784 | 0.003 |
| Isovaleryl-CoA dehydrogenase, mitochondrial | *ivd* | -1.780 | 0.004 |
| ? | *?* | -1.778 | 0.001 |
| Fos-related antigen 2 | *fosl2* | -1.778 | 0.007 |
| UV excision repair protein RAD23 homolog B | *rad23b* | -1.777 | 0.004 |
| Neurobeachin-like protein 2 | *nbeal2* | -1.772 | 0.002 |
| AH receptor-interacting protein | *aip* | -1.768 | 0.000 |
| Tubulin-specific chaperone A | *tbca* | -1.768 | 0.003 |
| Methylmalonyl-CoA mutase, mitochondrial | *mut* | -1.751 | 0.003 |
| ATP-dependent (S)-NAD(P)H-hydrate dehydratase | *carkd* | -1.745 | 0.001 |
| Cytosolic carboxypeptidase 6 | *agbl4* | -1.743 | 0.003 |
| Apoptosis-associated speck-like protein containing a CARD | *pycard* | -1.740 | 0.002 |
| Tetratricopeptide repeat protein 9C | *ttc9c* | -1.736 | 0.004 |
| Casein kinase II subunit beta | *csnk2b* | -1.729 | 0.002 |
| Xylosyltransferase 2 | *xylt2* | -1.721 | 0.003 |
| E3 ubiquitin-protein ligase RNF31 | *rnf31* | -1.711 | 0.008 |
| Arylsulfatase A | *arsa* | -1.710 | 0.003 |
| 28S ribosomal protein S33, mitochondrial | *mrps33* | -1.709 | 0.001 |
| Si:ch211-125c5.1 protein | *spen* | -1.705 | 0.001 |
| Structural glycoprotein p40 | *p40* | -1.691 | 0.010 |
| Vascular endothelial growth factor receptor 2 | *kdr* | -1.690 | 0.006 |
| Gamma-adducin | *add3* | -1.688 | 0.007 |
| Sorting nexin-4 | *snx4* | -1.684 | 0.010 |
| Arylsulfatase B | *arsb* | -1.681 | 0.005 |
| RNA, 18S Ribosomal | [*rna18s1*](http://www.genecards.org/cgi-bin/carddisp.pl?gene=RNA18S1&search=18S+ribosomal+RNA) | -1.676 | 0.008 |
| Inositol 1,4,5-trisphosphate receptor-interacting protein | *itprip* | -1.670 | 0.003 |
| Syntaphilin | *snph* | -1.666 | 0.005 |
| Probable E3 ubiquitin-protein ligase HERC6 | *herc6* | -1.666 | 0.008 |
| BTB (POZ) domain containing 1 | *btbd1* | -1.665 | 0.005 |
| Cell cycle progression protein 1 | *ccpg1* | -1.663 | 0.009 |
| Glutaredoxin-3 | *glrx3* | -1.661 | 0.003 |
| KCNAB1 protein | *kcnab1* | -1.659 | 0.008 |
| Cystatin-SN | *cst1* | -1.652 | 0.000 |
| Pre-mRNA-splicing factor 18 | *prpf18* | -1.647 | 0.000 |
| Porimin | *tmem123* | -1.643 | 0.004 |
| Acetyl-CoA carboxylase 1 | *acaca* | -1.643 | 0.008 |
| ATP synthase subunit alpha, mitochondrial | *atp5a* | -1.628 | 0.004 |
| Protein DJ-1 | *park7* | -1.628 | 0.008 |
| Zinc finger protein-like 1 | *zfpl1* | -1.625 | 0.006 |
| Glycosyltransferase-like domain-containing protein 1 | *gtdc1* | -1.624 | 0.005 |
| Eukaryotic translation initiation factor 3 subunit D | *eif3d* | -1.619 | 0.010 |
| Endophilin-B1 | *sh3glb1* | -1.617 | 0.004 |
| DNA-directed RNA polymerases I, II, and III subunit RPABC5 | *polr2l* | -1.604 | 0.005 |
| LIM/homeobox protein Lhx1 | *lhx1* | -1.596 | 0.007 |
| Interferon regulatory factor 1 | *irf1* | -1.595 | 0.009 |
| Peptide YY-like | *pyy* | -1.594 | 0.002 |
| Protein archease | *zbtb8os* | -1.585 | 0.002 |
| Spermidine synthase | *srm* | -1.575 | 0.002 |
| ? | *?* | -1.571 | 0.002 |
| Ribosomal protein 63, mitochondrial | *mrp63* | -1.564 | 0.008 |
| Protein BEX2 | *bex2* | -1.558 | 0.003 |
| Mitogen-activated protein kinase kinase kinase 1 | *map3k1* | -1.552 | 0.003 |
| Gamma-aminobutyric-acid receptor rho-2B subunit | *?* | -1.549 | 0.004 |
| ? | *?* | -1.540 | 0.007 |
| Torsin-1A-interacting protein 1 | *tor1aip1* | -1.539 | 0.008 |
| MKIAA4125 protein | *bicd1* | -1.532 | 0.001 |
| ? | *?* | -1.524 | 0.007 |
| 39S ribosomal protein L34, mitochondrial | *mrpl34* | -1.524 | 0.001 |
| Kelch-like protein 26 | *klhl26* | -1.518 | 0.009 |
| Histone-lysine N-methyltransferase SETDB2 | *setdb2* | -1.512 | 0.008 |
| ? | *?* | -1.507 | 0.005 |
