# Supplementary material for: Effects of Changes in Food Supply at the Time of Sex Differentiation on the Gonadal Transcriptome of Juvenile Fish. Implications for Natural and Farmed Populations
Source: PLoS One. 2014 Oct 23;9(10):e111304. doi: 10.1371/journal.pone.0111304 (PMC4207807; doi:10.1371/journal.pone.0111304)
Supplement: Table S16 — Affected KEGG pathways in the FS vs. SS group comparison. (DOCX) [file pone.0111304.s020.docx]

Supplementary Table 16. KEGG pathways for the FS versus SS comparison

| Pathways | # Sequences | # Enzymes | up/ down |
| --- | --- | --- | --- |
| Alanine, aspartate and glutamate metabolism | 3 | 4 | up |
| Alpha-linolenic acid metabolism | 3 | 3 | up |
| Amino sugar and nucleotide sugar metabolism | 5 | 5 | up |
| Aminoacyl-tRNA biosynthesis | 5 | 5 | up |
| Aminobenzoate degradation | 1 | 1 | up |
| Arachidonic acid metabolism | 2 | 2 | down |
| Arginine and proline metabolism | 5 | 5 | up |
| Benzoate degradation | 1 | 1 | up |
| Betalain biosynthesis | 1 | 1 | up |
| Biosynthesis of unsaturated fatty acids | 2 | 2 | up |
| Butanoate metabolism | 2 | 2 | up |
| Caprolactam degradation | 1 | 1 | up |
| Chloroalkane and chloroalkene degradation | 1 | 1 | down |
| Citrate cycle (TCA cycle) | 2 | 3 | up |
| Cutin, suberine and wax biosynthesis | 1 | 1 | up |
| Drug metabolism-other enzymes | 5 | 4 | up |
| Ether lipid metabolism | 5 | 4 | up |
| Fatty acid biosynthesis | 2 | 2 | down |
| Fatty acid degradation | 4 | 4 | up |
| Fatty acid elongation | 3 | 2 | up |
| Fructose and mannose metabolism | 4 | 4 | up |
| Galactose metabolism | 3 | 3 | up |
| Geraniol degradation | 1 | 1 | up |
| Glucerophospholipid metabolism | 8 | 7 | up |
| Glutathione metabolism | 5 | 5 | up |
| Glycerolipid metabolism | 6 | 5 | up |
| Glycerophospholipid metabolism | 1 | 1 | down |
| Glycine, serine and threonine metabolism | 2 | 2 | up |
| Glycolysis/Gluconeogenesis | 2 | 3 | up |
| Glycosaminoglycan biosynthesis-chondro | 1 | 1 | down |
| Glycosaminoglycan biosynthesis-heparan | 2 | 3 | down |
| Glycosaminoglycan degradation | 1 | 1 | up |
| Glycosphingolipid biosynthesis-ganglio | 1 | 1 | up |
| Glycosphingolipid biosynthesis-lacto | 1 | 2 | up |
| Glycosylphosphatidylinositol (GPI)-anchor biosynthesis | 2 | 2 | up |
| Glyoxylate and dicarboxylate metabolism | 2 | 2 | down |
| Linoleic acid metabolism | 2 | 1 | up |
| Lysine degradation | 6 | 4 | up |
| Methane metabolism | 1 | 1 | up |
| Naphthalene degradation | 1 | 1 | down |
| Nicotinate and nicotinamide metabolism | 3 | 3 | up |
| Nitrogen metabolism | 1 | 1 | up |
| One carbon pool by folate | 1 | 1 | up |
| Other glycan degradation | 2 | 2 | up |
| Oxidative phosphorylation | 7 | 5 | up |
| Pentose phosphate pathway | 4 | 4 | up |
| Phenylalanine metabolism | 3 | 4 | up |
| Phenylalanine, tyrosine and tryptophan biosynthesis | 1 | 1 | up |
| Phenylpropanoid biosynthesis | 2 | 1 | up |
| Phosphatidylinositol signaling system | 2 | 2 | up |
| Phosphonate and phosphinate metabolism | 1 | 1 | up |
| Porphyrin and chlorophyll metabolism | 2 | 4 | up |
| Primary bile acid biosynthesis | 2 | 2 | up |
| Propanoate metabolism | 3 | 2 | down |
| Purine metabolism | 24 | 19 | up |
| Pyrimidine metabolism | 16 | 12 | up |
| Pyruvate metabolism | 4 | 3 | up |
| Riboflavin metabolism | 2 | 1 | up |
| Selenocompound metabolism | 2 | 1 | up |
| Sphingolipid metabolism | 5 | 5 | up |
| Starch and sucrose metabolism | 2 | 2 | up |
| Steroid biosynthesis | 1 | 1 | up |
| Streptomycin biosynthesis | 2 | 2 | up |
| Synthesis and degradation of ketone bodies | 1 | 1 | up |
| T cell receptor signaling pathway | 2 | 2 | up |
| Terpenoid backbone biosynthesis | 2 | 2 | up |
| Toluene degradation | 1 | 1 | up |
| Tropane, piperidine and pyridine alkaloid biosynthesis | 1 | 1 | up |
| Tryptophan metabolism | 3 | 3 | up |
| Tyrosine metabolism | 2 | 1 | up |
| Ubiquinone and other terpenoid-quinone | 1 | 1 | up |
