# Supplementary material for: Effects of Changes in Food Supply at the Time of Sex Differentiation on the Gonadal Transcriptome of Juvenile Fish. Implications for Natural and Farmed Populations
Source: PLoS One. 2014 Oct 23;9(10):e111304. doi: 10.1371/journal.pone.0111304 (PMC4207807; doi:10.1371/journal.pone.0111304)
Supplement: Table S17 — Two-tails Fisher's exact test with Multiple Testing Correction for FDR results for the FS vs. SS group comparison. (DOCX) [file pone.0111304.s021.docx]

Supplementary Table 17. Fisher’s Exact Test with Multiple Correction for FDR for FS versus SS comparison

| GO Term | Name | Type | FDR | single test p-Value | # in test group | # in reference group | Over/ |
| --- | --- | --- | --- | --- | --- | --- | --- |
|  |  |  |  |  |  |  | Under |
| [GO:0005840](FisherInfo:GO:0005840) | Ribosome | CC | 6,50E-06 | 2,60E-09 | 73 | 245 | over |
| [GO:0003735](FisherInfo:GO:0003735) | structural constituent of ribosome | MF | 4,90E-05 | 4,90E-08 | 46 | 130 | over |
| [GO:0006412](FisherInfo:GO:0006412) | Translation | BP | 1,00E-03 | 1,70E-06 | 78 | 324 | over |
| [GO:0005739](FisherInfo:GO:0005739) | Mitochondrion | CC | 1,20E-03 | 2,10E-06 | 152 | 779 | over |
| [GO:0000028](FisherInfo:GO:0000028) | ribosomal small subunit assembly | BP | 1,20E-03 | 2,20E-06 | 6 | 0 | over |
| [GO:0002682](FisherInfo:GO:0002682) | regulation of immune system process | BP | 1,60E-02 | 4,50E-05 | 6 | 184 | under |
| [GO:0005730](FisherInfo:GO:0005730) | Nucleolus | CC | 5,00E-02 | 1,70E-04 | 83 | 406 | over |
